# If these aren’t genuine species, what are? An analysis of heterotypic synonyms contributes to 19 new names and 20 new combinations in *Magnolia* (Magnoliaceae). Part 2: New combinations

**DOI:** 10.3897/phytokeys.277.195893

**Published:** 2026-07-10

**Authors:** Christopher B. Callaghan, Siak K. Png

**Affiliations:** 1 Australian Bicentennial Arboretum, P.O. Box 88, Penshurst, NSW 2222, Australia Australian Bicentennial Arboretum Penshurst Australia

**Keywords:** Conservation, endangered species, Magnolioideae, reinstated species, unsubstantiated synonymy

## Abstract

In 2026, it is almost universally recognised that the family Magnoliaceae includes two subfamilies, Liriodendroideae and Magnolioideae, each subfamily consisting of one genus, *Liriodendron* L., and *Magnolia* L., respectively. In recognition of this, the authors in the first part of this research monograph reinstated 19 taxa from unsubstantiated synonymy and then transferred these taxa to *Magnolia* with proposed new replacement names, in accordance with Article 53.1 of the current 2024 ICN Madrid Code. These new names were required because their transferal to *Magnolia* without a change of name would otherwise have created a later illegitimate homonym to an existing validly published name of a living or fossil taxon.

In this second part of the research monograph, a further 20 taxa from the former segregate genera of *Manglietia* and *Michelia* are reinstated, where necessary, from misconceived synonymy and transferred to *Magnolia* as new combinations. Subsequent to their valid naming and description as new species or varieties, the majority of these taxa, most of which were discovered in the last decades of the twentieth century due to their small natural populations and distributions, have been reduced as unsubstantiated heterotypic synonyms under previously named, closely related taxa of generally wide-spread occurrence. Unfortunately, this synonymising has often been by a few influential, but unfortunately misguided and ill-informed botanical researchers, with evidently dire consequences for the survival of numerous of these genuine species and varieties in their native habitats. Frequently these later-named taxa were synonymised under those that their original naming author(s), in compliance with the requirements for naming new taxa specified in the then current International Code of Botanical Nomenclature (ICBN), had taken into consideration in their comparative diagnosis which accompanied the formal published description of their new species or variety. These taxa are now restored because, based on extensive comparative data compiled by the present authors, it has been determined that they are the unique independent species or varieties as they were originally named and described by their respective authors.

These new combinations in *Magnolia* are in respect of the following predominately occurring eastern and south-eastern Asian taxa:

*Manglietia tenuipes*, Michelia balansae var. appressipubescens, *M. brevipes*, *M. chingii*, *M. chongjiangensis*, M. floribunda var. lanea, *M. fulgens*, *M. glaberrima*, *M. hedyosperma*, *M. kerrii*, *M. linyaoensis*, *M. longistamina*, *M. polyneura* and *M. wardii*. Also, since most Chinese Magnoliaceae authorities continue to recognise *Manglietia calcarea*, *M. forrestii* and *M. hainanensis* as authentic species and not as varieties of *Manglietia fordiana*, plus *Michelia calcicola* and *M. skinneriana* as genuine independent species and not as varieties of *M. fulva* and *M. figo* respectively, each of these five taxa are reinstated and transferred to *Magnolia*. Finally, as a result of its distinctive features, Michelia macclurei var. sublanea has been elevated to species status and transferred to *Magnolia* as *M. sublanea*.

As in the first part of this research monograph, some notes and recommendations on the *in situ* and *ex situ* conservation of each of the above taxa are presented. It has again been determined that many of these taxa have never been evaluated in their native habitats, because in past decades they have been reduced to unsubstantiated synonyms, resulting in them being frequently hidden under the names of more common and widely distributed species. From the present research over many years, it is evident that a number of these reductions have adversely impacted the survival prospects of numerous threatened and endangered *Magnolia* taxa, since they have no longer been reported from their previously recorded localities or elsewhere in the wild, at least under their original names, in decades.

## Table of contents

Introduction 79

Materials and methods 80

Repositories 81

Results 81

Discussion 82

Final conclusions 82

Taxonomy, conservation status and recommendations 82

Part 2: New combinations in *Magnolia* 83

Magnolia balansae var. appressipubescens (Law) C.B. Callaghan & S.K. Png, comb. nov. 83

*Magnolia brevipes* (Y.K. Li & X.M. Wang) C.B. Callaghan & S.K. Png, comb. nov. 84

*Magnolia calcarea* (X.H. Song) C.B. Callaghan & S.K. Png, comb. nov. 87

*Magnolia calcicola* (C.Y. Wu ex Y.H Liu & R.F. Wu) C.B. Callaghan & S.K. Png, comb. nov. 92

*Magnolia chingii* (W.C. Cheng) C.B. Callaghan & S.K. Png, comb. nov. 96

*Magnolia congjiangensis* (Y.K. Li & X.M. Wang) C.B. Callaghan & S.K. Png, comb. nov. 98

Magnolia floribunda var. lanea (Y.K. Sima) C.B. Callaghan & S.K. Png, comb. nov. 101

*Magnolia forrestii* (W.W. Sm. ex Dandy) C.B. Callaghan & S.K. Png, comb. nov. 102

*Magnolia fulgens* (Dandy) C.B. Callaghan & S.K. Png, comb. nov. 104

*Magnolia glaberrima* (Hung T. Chang) C.B. Callaghan & S.K. Png, comb. nov. 108

*Magnolia hainanensis* (Dandy) C.B. Callaghan & S.K. Png, comb. nov. 110

*Magnolia hedyosperma* (Law) C.B. Callaghan & S.K. Png, comb. nov. 113

*Magnolia kerrii* (Craib) C.B. Callaghan & S.K. Png, comb. nov. 116

*Magnolia linyaoensis* (D.C. Zhang & S.B. Zhou) C.B. Callaghan & S.K. Png, comb. nov. 118

*Magnolia longistamina* (Law) C.B. Callaghan & S.K. Png, comb. nov. 119

*Magnolia polyneura* (C.Y. Wu ex Law & Y.F. Wu) C.B. Callaghan & S.K. Png, comb. nov. 123

*Magnolia skinneriana* (Dunn) C.B. Callaghan & S.K. Png, comb. nov. 128

*Magnolia sublanea* (Dandy) C.B. Callaghan & S.K. Png, comb. et stat. nov. 129

*Magnolia tenuipes* (Dandy) C.B. Callaghan & S.K. Png, comb. nov. 131

*Magnolia wardii* (Dandy) C.B. Callaghan & S.K. Png, comb. nov. 135

Acknowledgements 137

References 138

Appendix 1 - Institutional herbarium acronyms 148

Additional information 151

## Introduction

**Figures 1, 2.**
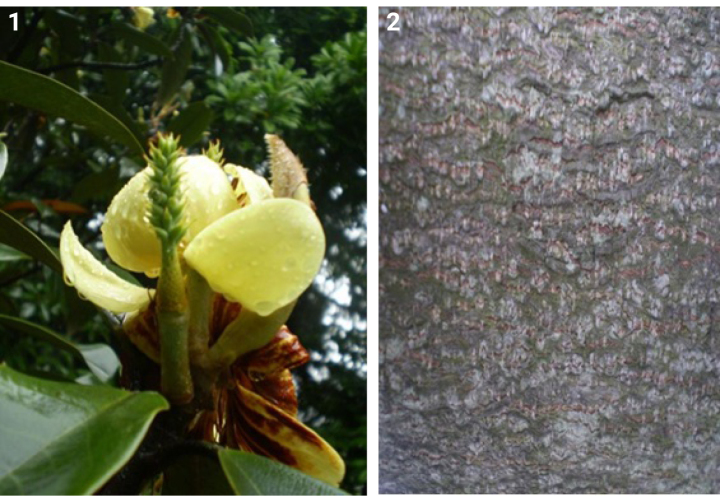
**1**. *Magnolia calcicola* (*Michelia calcicola*). Flower with gynoecium in front. Photo taken by the authors at Kunming Botanical Garden, Heilongtan, Kunming, Yunnan, 17 April 2017. © Callaghan & Png (ABA); **2**. *Magnolia fulgens* (*Michelia fulgens*). Silver-grey bark. Photo taken by the authors at the arboretum of Guizhou Academy of Forestry, Guiyang, Guizhou. 12 April 2017. © Callaghan & Png (ABA).

As noted in the preliminary introduction included at the commencement of the first part of this monograph, the present authors came to conclude that numerous Magnoliaceae taxa occurring in China that are recognised as species or varieties in Magnolias of China (Liu et al. 2004) and in the treatment of Magnoliaceae in its predecessor Flora Republicae Popularis Sinicae (Law 1996), particularly in the genera *Manglietia* and *Michelia*, had prior to these two Chinese publications been sunk into synonymy with earlier-named species of these segregate genera. This had occurred predominately in Notes on Magnoliaceae III: The Magnoliaceae of China (Chen and Nooteboom 1993), and most of these were subsequently listed as accepted synonyms under Magnoliaceae authored by Xia, Liu and Nooteboom in Volume 7 of Flora of China, published in December 2008. (Note: the middle author, the leading Chinese Magnoliaceae authority Liu Yu-hu, a.k.a. Law Yu-wu, had died four and a half years earlier in May 2004!). Interestingly, in Magnolias of China, the references at page 380 that include both Chinese and international publications relating to Magnoliaceae, have ignored Chen and Nooteboom’s 1993 publication, indicating that the Chinese authors of the 2004 publication apparently did not accept Nooteboom’s treatment of Chinese Magnoliaceae, such as synonymising *Michelia calcicola* under *M. ingrata* and *M. fulgens* under *M. foveolata* (Figs 1, 2).

The Magnoliaceae of China had been finalised in the absence of the ‘lead author’ Chen Bao-liang who, disillusioned after an apparent falling out with Nooteboom, had returned home to China with his research notes in May 1991 (more than two and a half years before publication of The Magnoliaceae of China in the quarterly published journal Annals of the Missouri Botanical Garden in November 1993!), where the resultant stress of his involvement with Nooteboom may have contributed to his death from cancer four months later in September 1991 at only 47 years of age. Chen’s name was subsequently utilised to give credence to Nooteboom’s treatment of Chinese Magnoliaceae, a strategy used elsewhere with Magnoliaceae. See more information at Callaghan and Png (2026a).

The realisation that many of these synonyms are not accepted, but are recognised as independent taxa by numerous Chinese botanists and plant taxonomists, including Deng et al. (2015), Li J.J. (2021), Li et al. (2020), Qin et al. (2017), Wang et al. (2020) and Yang et al. (2016), as are referenced where appropriate in the paper, paved the way for more than a decade of research by the present authors to determine whether these synonymised Chinese taxa, plus a number of others from adjacent countries, are justified as heterotypic synonyms of earlier-named taxa, or are in fact the independent, genuine species or varieties as they were originally named and described.

## Materials and methods

The materials utilised in this study, plus the methods followed to determine whether numerous previously synonymised taxa in various segregate genera of Magnoliaceae are, in fact, the genuine new species or varieties originally named by their respective author(s), are outlined in the Materials and Methods section of Part 1 of this paper (Callaghan and Png 2026a). This initial paper dealt with those taxa whose substantiation and reinstatement required new replacement names when transferred to *Magnolia*. This was to avoid the creation of a later illegitimate homonym to an existing validly published name of a living or fossil taxon, in compliance with the rules and recommendations of the 2024 International Code of Nomenclature for algae, fungi and plants (ICN), known as The Madrid Code (Turland et al. 2025). In this second part of the paper, a further 20 taxa from the former segregate genera of *Manglietia* and *Michelia* are transferred to *Magnolia* as new combinations, the majority having also been reinstated from unsubstantiated synonymy, as evidenced from their comparative morphological features derived from the literature and compiled in their respective comparison tables. These new combinations are made in accordance with ICN Article 41 and Recommendation 41A in respect of new combinations (Turland et al. 2025).

### Repositories

Approximately 154 images of type specimens or other specimen images of the relevant taxa deposited at the following 29 herbaria have been sighted: A, BM, E, GFS, GZAC, HGAS, HITBC, HK, HSBL, IBK, IBSC, K, KUN, LBG, LE, LU, NAS, NF (HNIF), NHN/L, NY, P, PE, PH, SYS, SZG, TCD, US, WUK, YAF. Their images are either posted to the websites of the above herbaria located in various countries, to the Chinese Virtual Herbarium (CVH) website http://www.cvh.ac.cn, to JSTOR Global Plants http://plants.jstor.org, or have been emailed to the authors. These are indicated by an exclamation mark (!) after the herbarium acronyms or the barcodes of the individual specimens, with the names of the herbaria that are represented by the above acronyms, plus a number of herbaria in which relevant unsighted specimens are held, being compiled in a table located after the references.

## Results

From the present authors’ research since publication of our initial *Magnolia* paper (Callaghan and Png 2013), it has been determined that numerous validly published, legitimate taxa in various segregate genera of subfamily Magnolioideae, have in past decades been relegated, without justification, to unsubstantiated synonymy under previously named taxa and subsequently listed as such by a number of authors in various publications and on internet sites. This, together with the present widespread and near universal acceptance of subfamily Magnolioideae to consist of the single genus *Magnolia*, resulted in the reinstatement and transfer of twelve of these formerly synonymised taxa to *Magnolia* in the authors’ previously published paper transferring 26 taxa from former segregate genera to *Magnolia* (Callaghan and Png 2020). Incredibly, despite the overwhelming evidence presented in this previous paper, these twelve reinstated species are still listed, after six years, in synonymy on various online botanical databases! It needs to be said here that there has been a concerted on going campaign since 2020 to prevent publication of our previously submitted monograph that sought to reinstate numerous unsubstantiated synonymised taxa and ensure our research never sees the light of day, since those responsible consider conservation of reputations is more important than conservation of obviously threatened and endangered species.

Despite this, an additional 13 new combinations are now made to transfer to *Magnolia* the following formerly misconceived synonymised taxa that are herein confirmed as genuine independent taxa and therefore reinstated: *Manglietia tenuipes* Dandy, Michelia balansae var. appressipubescens Law, *M. brevipes* Y.K. Li & X.M. Wang, *M. chingii* W.C. Cheng, *M. chongjiangensis* Y.K. Li & X.M. Wang, *M. fulgens* Dandy, *M. glaberrima* Hung T. Chang, *M. hedyosperma* Law, *M. kerrii* Craib, *M. linyaoensis* D.C. Zhang & S.B. Zhou, *M. longistamina* Law, *M. polyneura* C.Y. Wu ex Law & Y.F. Wu and *M. wardii* Dandy.

Also, numerous Chinese *Magnolia* authorities have recognised *Manglietia calcarea* X.H. Song, *M. forrestii* W.W. Sm. ex Dandy and *M. hainanensis* Dandy as species, not as varieties of *M. fordiana* Oliv., and also *Michelia calcicola* C.Y. Wu ex Law & Y.F. Wu and *M. skinneriana* Dunn as species, not as varieties of *M. fulva* Hung T. Chang & B.L. Chen and *M. figo* (Lour.) Spreng., respectively. Their species status is confirmed in this paper, and each is transferred to *Magnolia*. Similarly, Magnolia macclurei var. sublanea Dandy is determined to be an authentic species and transferred to *Magnolia* as *M. sublanea*. Finally, Michelia floribunda var. lanea is herein transferred to *Magnolia*.

## Discussion

When previously published and accepted Magnoliaceae taxa are synonymised without proper substantiation by a comprehensive comparison with other closely related taxa, as has been determined to have occurred in the past, it has apparent negative consequences for the conservation and survival of these ill-conceived synonymised taxa, especially those that had small or widely dispersed and fragmented populations, which was the reason for their relatively late discovery in past decades.

The synonymised taxa researched in the following pages were found to have substantially more morphological differences than is usually noted and frequently tabulated by taxonomic authors in their required comparative diagnosis accompanying their descriptions of new plant species or varieties at their time of publication. Even allowing for possible regional variability, the comparative morphological and phenological features etc., compiled in the various tables for the taxa dealt with in the following pages reveal there to be an average of **22 differences** between each of these synonymised taxa and those they have been placed under, thus supporting these taxa as genuine species or varieties and consequently resulting in their reinstatement and transferral to *Magnolia* by the authors.

## Final conclusions

Now that they are once again recognised and reinstated as the independent species or varieties as they were previously determined in past decades by their naming authors and accepted for publication following peer review, it is imperative to determine their present *in situ* and *ex situ* conservation status at the earliest opportunity and to take the appropriate action to prevent their potential extinction, if this hasn’t unfortunately already been the fate of more than a few of these taxa buried under unsubstantiated synonymy for decades, as is apparent in respect of two species in our other paper (Callaghan and Png 2026b).

There are two choices now facing the botanical community – preservation or decimation of these mostly little-known, under-researched and now reinstated species!

## Taxonomy, conservation status and recommendations

Class Magnoliopsida Brongn.

Order Magnoliales Juss. ex Bercht. & J. Presl.

Family Magnoliaceae Juss.

Subfamily Magnolioideae Law

Genus *Magnolia* L.

### Part 2: New combinations in *Magnolia*

#### Magnolia balansae var. appressipubescens

Taxon classificationPlantaeMagnolialesMagnoliaceae

(Law) C.B. Callaghan & S.K. Png comb. nov.

9A4ABE0E-90C6-58F5-926B-9A2AFF1C27EF

urn:lsid:ipni.org:names:77382802-1

Michelia balansae var. appressipubescens Law, Bulletin of Botanical Research (Harbin) 5(3): 124 (Law 1985). **[Basionym]**.
Michelia balansae (A. DC.) Dandy, in Annals of Missouri Botanical Garden 80(4): 1079 (Chen and Nooteboom 1993), Flora Yunnanica. Tomus 6 (Spermatophyta): 47 (Wu and Chen 2006) and Flora of China Vol. 7: 89 (Xia et al. 2008), each p.p. quoad syn. Michelia balansae var. appressipubescens Law.
Magnolia balansae Aug. DC., online at World Checklist of Magnoliaceae (Govaerts 2003), p.p. quoad syn. Michelia balansae var. appressipubescens Law.
Michelia balansae var. appressipubescens Law, in Vouchered Flora of Southeast Yunnan, Vol. 1: 49 (Sima and Lu 2009), p.p. excl. syns. *Michelia masticate Dandy and Magnolia masticata* (Dandy) Figlar.

##### Present Chinese names.

细毛含笑 meaning “fine-haired michelia”, referring to various parts that are covered with appressed fine hairs, or the little-known Chinese name as was originally published: 细毛苦梓含笑 meaning “fine-haired bitter michelia”.

##### Proposed Chinese name.

细毛木兰 meaning “fine-haired magnolia”.

##### Type.

China. **Hainan Province** • Ding’an County, Mochong Shan, Jungkap, Jingan, in woods, 8 m tree, 30 Apr. 1932, *S.P. Ko* (*Gao Xi-peng*) *52279*. holotype: IBSC 0003280 n.v. isotypes: A 00039041! NY 00320710! IBK n.v.

holotype (IBSC) and isotype (IBK): specimen images not uploaded to CVH at 18 Mar. 2026.

**Paratypes**. China. **Hainan Province** • Bao-ting County, alt. ca. 120 m, 18 Aug. 1935, fr, *F.C. How (How Foon-chew*) *73480*. IBK 00000433! • Jingzhong,14 Dec. 1956, *Deng Liang 3703*. KUN 41799 n.v. – **Guangdong Province** • Mulan Park, June 1974, *Law Yu-wu 6016*, *6037*IBSC 0053010 n.v. IBSC 0053011 n.v. – **Guizhou Province** • Xing-yi, 29 July 1923, *P.C. Chung (Tsoong Pu-chin*) *1572*. KUN 42663 n.v. – **Yunnan Province** • Dawei Shan, Jinzhuping, alt. 3600 m, Apr. 1940, *H. Wang* (*Wang Xiao*), *S.P. Ko* (*Gao Xi-peng*) *& S.K. Lau* (*Liu Xin-qi*) *100349*. IBSC 0053086 n.v. • Pingbian Miao Autonomous County, alt. 1000 m, 16 Mar. 1954, *Mao Pin-yi 3426*. KUN 41783 n.v. WUK 0208207 n.v.

holotype (IBSC), paratypes (IBSC, KUN, WUK): specimen images not uploaded to CVH at 18 Mar. 2026.

Specimen images below accessed 20 Mar. 2019. IBK re-accessed with new URL 20 Oct. 2022:

isotype (A ex NY): http://kiki.huh.harvard.edu/databases/image.php?id=304816

isotype (NY): http://sweetgum.nybg.org/science/vh/specimen_details.php?irn=413547

paratype (IBK): https://www.cvh.ac.cn/spms/detail.php?id=c0077bf5

##### Note 1.

Michelia balansae var. appressipubescens Law whose buds, young twigs, petioles, undersides of the slightly larger leaves and peduncles are covered with appressed fine hairs as opposed to those of *Michelia balansae* (A. DC.) Dandy being described as densely brown tomentose (Liu et al. 2004: 220), is treated as a synonym of the latter in Chen and Nooteboom (1993), because “a continuous variation of the indumentum can be seen within the species”. While this synonym is also listed in Xia et al. (2008), the variety, which also matures its fruit a month earlier than the species, was retained in Law (1996: 168), Liu et al. (2004: 222), Sima and Lu (2009), Sima (2011: 242) and Yang et al. (2016: 233), and is consequently reinstated and transferred to *Magnolia*.

##### Note 2.

Research for a future paper has found Michelia balansae var. appressipubescens recorded in the literature as occurring in China at two nature reserves and cultivated at two botanical gardens and one forestry research institute.

##### Recommendation.

*Magnolia balansae*, with Michelia balansae subsp. appressipubescens [sic] included as a synonym, was assessed on 31 August 2012 for the IUCN Red List of Threatened Species (IUCN 2026) as “Data Deficient with a decreasing current population trend”. An assessment of Magnolia balansae var. appressipubescens independent of *Magnolia balansae* needs to be undertaken to determine its IUCN Red List classification.

#### Magnolia brevipes

Taxon classificationPlantaeMagnolialesMagnoliaceae

(Y.K. Li & X.M. Wang) C.B. Callaghan & S.K. Png comb. nov.

5F50D8C7-2708-5FA9-8D30-11986EFC40FC

urn:lsid:ipni.org:names:77382803-1

Michelia brevipes Y.K. Li & X.M. Wang, Acta Phytotaxonomica Sinica 25(5): 408, fig. 1 (Li and Wang 1987). **[Basionym]**.
Michelia figo (Lour.) Spreng., in Annals of Missouri Botanical Garden 80(4): 1084 (Chen and Nooteboom 1993), p.p. quoad syn. Michelia brevipes Y.K. Li & X.M. Wang.
Michelia figo (Lour.) Spreng. var. figo, in World Checklist and Bibliography of Magnoliaceae: 56 (Frodin and Govaerts 1996), p.p. quoad syn. Michelia brevipes Y.K. Li & X.M. Wang.
Michelia crassipes Law, in Flora of China Vol. 7: 87 (Xia et al. 2008), p.p. quoad syn. Michelia brevipes Y.K. Li & X.M. Wang.
Michelia yunnanensis Franch. ex Finet & Gagnep., in A Taxonomic Revision of the Magnolia ceae from China: 236 (Sima 2011) and Magnoliaceae Plants of Guizhou: 169 (Deng et al. 2015), both p.p. quoad syn. Michelia brevipes Y.K. Li & X.M. Wang.

##### Present Chinese name.

短梗含笑 meaning “short-stalked michelia” (viz. the fruit peduncle).

##### Proposed Chinese name.

短梗水果木兰 meaning “short-stalked fruit magnolia”.

##### Type.

China. **Guizhou Province** • Anlong County, Longtou Dashan, alt. 1710 m, ca. 24 July 1981, *Dang Cheng-zhong 913*. holotype: HGAS n.v. isotype: IBSC 0054642 n.v.

**Paratypes. ibid**. • alt. 1710 m, shrub 3.5 m, 24 July 1981, fr, *Dang Cheng-zhong 919*. HGAS 010745! • alt. 1714 m, ca. 23 July 1981, fr, *Dang Cheng-zhong 886*. HGAS 010593!

holotype (HGAS): specimen image not uploaded to CVH at 6 Nov. 2022 (see note 1 below).

isotype (IBSC): specimen image not uploaded to CVH at 18 Mar. 2026.

Specimen images below accessed 20 Mar. 2019; found again with new URLs under *Michelia crassipes*.

paratype (HGAS): https://www.cvh.ac.cn/spms/detail.php?id=e4687e5d [accessed 6 Nov. 2022].

paratype (HGAS): https://www.cvh.ac.cn/spms/detail.php?id=e4683cd0 [accessed 28 Dec. 2022].

##### Note 1.

The holotype specimen for *Michelia brevipes* at HGAS could not be found (Chen Xiang, pers. comm., July 2019).

##### Note 2.

From their accompanying diagnosis, it is evident that Li and Wang considered their newly described species *Michelia brevipes* as distinct from *M. figo* under which it was later treated as a synonym in Chen and Nooteboom (1993) “on the basis of its description and published figure”. It is also recorded as a synonym of *M. crassipes* and of *M. yunnanensis* by the authors previously noted. However, the present authors consider that *Michelia brevipes* from Guizhou can be easily distinguished from the more widespread occurring *M. crassipes* and the cultivated *M. figo* by the differentiating features compiled in Tables 1, 2, and from *M. yunnanensis* by the differentiating features compiled in Table 3.

**Table 1.** The **17 presently known** differentiating features between *Michelia brevipes* Y.K. Li & X.M. Wang and *Michelia crassipes* Law.

**Plant feature**	***Michelia brevipes***	***Michelia crassipes***
ultimate height	3.5 m	5 m
indumentum of buds	appressed ferrugineus-pubescent	brown tomentose
indumentum of young twigs	appressed ferrugineus-pubescent	brown tomentose
leaf shape	narrowly elliptic-obovate or elliptic-obovate	narrowly obovate or narrowly elliptic
**leaf dimensions**	**3.2–9 × 2.2–3.2 cm**	**7–13 × 2.5–3.5(–4^2^) cm**
leaf apex	abruptly acute	acuminate or acute
leaf indumentum below	appressed ferrugineus-pubescent	glabrous surface with pilose veins
**petiole length and stipule connectivity**	**5–10 mm, with a 1 mm stipular scar**	**2–4 mm, with fully adnate stipules (no scar)**
petiole indumentum	rusty-red pubescence	brown tomentose
**gynoecium**	**overtopping stamens**	**hidden within stamens**
**gynophore in fruit**	**18–24 mm long**	**1–2 mm long**
**fruit peduncle length**	**5–6 mm**	**10–20 mm**
fruit peduncle indumentum	dense rusty-red pubescence	reddish-brown or yellowish-brown tomentose**^2^**
**fruit aggregate length**	**ca. 8 cm**	**2.5–5 cm**
mature carpel number	few, as most aborted	more than 10**^1^**
mature carpel shape	ovoid	compressed ovoid or compressed globose**^2^**
**fruiting period**	**July [paratype HGAS]**	**August–September^2^**

Note: The listed features of *Michelia brevipes* are cited from Li and Wang (1987: 408); those of *Michelia crassipes* are from Law (1985: 121), supplemented by Law (1996: 163)**^1^** and Liu et al. (2004: 240)**^2^**.

**Table 2.** The **20 presently known** differentiating features between *Michelia brevipes* Y.K. Li & X.M. Wang and *Michelia figo* (Lour.) Spreng.

**Plant feature**	***Michelia brevipes***	***Michelia figo***
ultimate height	3.5 m	5 m
indumentum of buds	appressed ferrugineus-pubescent	densely yellowish-brown tomentose**^4^**
indumentum of young twigs	appressed ferrugineus-pubescent	densely yellowish-brown tomentose**^4^**
leaf shape	narrowly elliptic-obovate or elliptic-obovate	narrowly elliptic or obovate-elliptic
leaf dimensions	3.2–9 × 2.2–3.2 cm	4–10 × 1.8–4.5 cm
leaf apex	abruptly acute	obtusely short-acute
leaf indumentum abaxially	appressed ferrugineus-pubescent	residual brown appressed pilose hairs along midribs
**petiole length**	**5–10 mm**	**3–5 mm**
petiole indumentum	rusty-red pubescence	densely brown pubescent**^4^**
**stipule connectivity**	**1 mm stipular scar**	**fully adnate stipules**
**flower bud bract number**	**3**	**1^6^**
**flower peduncle length**	**ca. 3 mm**	**12.7 mm^3^**
**gynophore length in fruit**	**18–24 mm**	**3–4 mm**
**fruit peduncle length**	**5–6 mm**	**to 10 mm^5^**
fruit peduncle indumentum	dense rusty-red pubescence	densely yellowish-brown tomentose
**fruit aggregate length**	**ca. 8 cm**	**2–3.5 cm**
**mature carpel number**	**few, as most aborted**	**1–15^1^**
mature carpel shape	ovoid.	ovoid or globose
mature carpel dimensions	ca. 15 mm long	7–12 × 7–9 mm**^1^**
fruiting period	July [paratype HGAS]	August–September (July–August**^2^**) [a cultivated taxon, so where it is grown in China determines fruiting time]

Note: The listed features of *Michelia brevipes* are cited from Li and Wang (1987: 408); with those of *Michelia figo* from Liu et al. (2004: 250), supplemented by Chen and Nooteboom (1993: 1084)**^1^**, Law (1996: 165)**^2^**, Bean (1973: 738)**^3^**, Krüssmann (1985: 307)**^4^**, Spencer (1997: 23)**^5^** and Spongberg (1998: 134)**^6^**.

**Table 3.** The **17 presently known** differentiating features between *Michelia brevipes* Y.K. Li & X.M. Wang and *Michelia yunnanensis* Franch. ex Finet et Gagnep.

**Plant feature**	***Michelia brevipes***	***Michelia yunnanensis***
**life form**	**shrub to 3.5 m**	**small tree to 12 m^1^ (10+ m at Australian Bicentennial Arboretum)**
indumentum of young twigs	rusty-red appressed pubescent	densely dark red appressed pilose**^4^**
leaf shape	narrowly elliptic-obovate or elliptic-obovate	obovate or narrowly obovate-elliptic**^4^**
leaf dimensions	3.2–9 × 2.2–3.2 cm	4–10 × 1.5–3.5 cm**^4^**
leaf apex shape	abruptly acute	obtuse or short-acute**^4^**
leaf indumentum abaxially	appressed ferrugineus-pubescent	densely dark red appressed pilose when young**^4^** residually appressed pilose
**petiole length**	**5–10 mm**	**4–5 mm^3^**
petiole indumentum	ferrugineus-pubescent	densely dark red appressed pilose**^4^**
**stipular scar length**	**ca. 1 mm**	**exceeding 2/3 of petiole length^4^**
**flower bud length**	**1.2–1.4 cm**	**2–3 cm^2^**
**tepal number**	**6**	**6–12(–17)^4^**
**gynophore length**	**18–24 mm**	**7–10 mm^2^**
**fruit aggregate length**	**ca. 8 cm**	**1–4.5 cm^2^**
mature carpels shape	ovoid	compressed globose
**mature carpels size**	**ca. 15 mm long**	**7–8 mm diameter**
fruiting period	July [paratype HGAS]	August–September**^4^**
**distribution**	**Longtou Dashan, Anlong County, southwestern Guizhou Province**	**central and southern Yunnan Province^4^**

Note: The listed features of *Michelia brevipes* are from Li and Wang (1987: 408); those of *Michelia yunnanensis* are from Finet and Gagnepain (1906a: 43) supplemented by Anon (2022)**^1^**, Chen and Nooteboom (1993: 1086)**^2^**, Law (1996: 163)**^3^** and Liu et al. (2004: 232)**^4^**.

##### Note 3.

From the comparisons compiled in the three tables, these are evidently four distinct species, plus *Michelia brevipes* also does not key out with the original validating descriptions for *M. skinneriana* Dunn and *M. amoena* Q.F. Zheng & M.M. Lin, nor match the glabrous, elliptic-leaved *Magnolia fuscata* Andrews (Andrews 1802: plate 229), all with whom it shares synonymy under *Michelia figo* in Chen and Nooteboom (1993). As an obviously genuine species, *Michelia brevipes* is reinstated and transferred to *Magnolia*.

##### Note 4.

Since *Michelia crassipes* was found in crossing experiments to be reproductively incompatible with *M. yunnanensis* (Wang et al. 2003), it is probable that *M. brevipes* would likewise be incompatible, so experiments should be conducted to determine if this is the case.

##### Note 5.

While *M. brevipes* has been found protected in two nature reserves, no record of this species in cultivation has been found in a search of the literature for a subsequent paper.

##### Conservation status.

*Michelia brevipes* Y.K. Li & X.M. Wang is not noted on the IUCN Red List of Threatened Species (IUCN 2026), but was previously recorded as a rare Guizhou endemic 25 years ago (Zou 2001: 48).

##### Recommendation.

An evaluation of the IUCN conservation status of *Magnolia brevipes*, independent of the other species under which *Michelia brevipes* has to date been subsumed in synonymy, should be undertaken at the earliest opportunity!

#### Magnolia calcarea

Taxon classificationPlantaeMagnolialesMagnoliaceae

(X.H. Song) C.B. Callaghan & S.K. Png comb. nov.

4D04BC61-21FF-5BAF-BB7F-84FA753ECBBF

urn:lsid:ipni.org:names:77382804-1

**Figures 3, 4.**
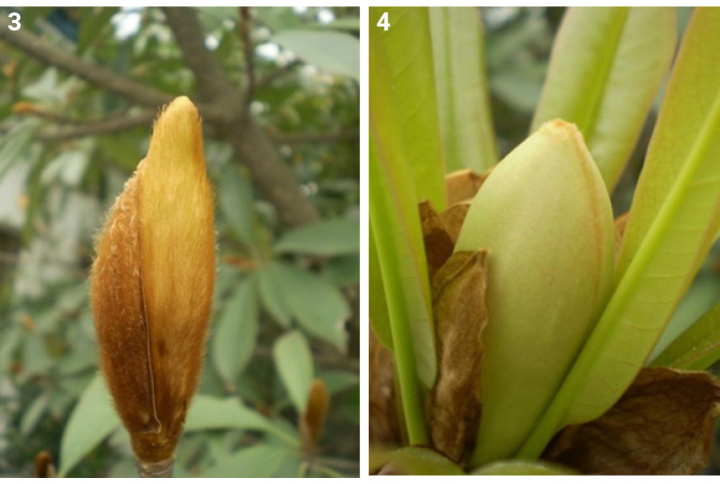
*Magnolia calcarea* (*Manglietia calcarea*). Expanding new shoot bud and flower bud. Photos taken by the authors at the Arboretum of Guizhou Academy of Forestry, Guiyang, 12 April 2017. © Callaghan & Png (ABA).

Tables 4, 5, Figs 3, 4

Manglietia calcarea X.H. Song, Journal of Nanjing Institute of Forestry 1984(4): 46, fig. 1 (Song 1984). **[Basionym]**.
Manglietia fordiana Oliv. var. calcarea (X.H. Song) B.L. Chen & Noot., in Annals of Missouri Botanical Garden 80(4): 1040 (Chen and Nooteboom 1993), p.p. excl. specimen *J. Cavalerie 2263*—World Checklist and Bibliography of Magnoliaceae: 51 (Frodin and Govaerts 1996).
Magnolia fordiana (Oliv.) Hu var. calcarea (X.H. Song) V.S. Kumar, in Kew Bulletin 61(2): 184. (Kumar 2006).
Manglietia aromatica Dandy var. calcarea (X.H. Song) Sima & S.G. Lu, in Journal of West China Forestry Science 43(1): 98 (Sima et al. 2014).

##### Present Chinese name.

石山木莲 meaning “stone hill manglietia” (refers to the terrain).

##### Proposed Chinese name.

荔波县石山木兰 meaning “Libo County stone hill magnolia”.

##### Type.

China. **Guizhou Province** • Libo County, Mogan, alt. 670 m, 30 Apr. 1983, fl, *Song Xiang-hou & Gao Feng 640*. holotype: NF (HNIF)! isotypes: IBSC n.v.

**Paratypes. Libo County** • alt. 790 m, no date, *Song Xiang-hou 1211*. NF (HNIF) n.v. • **Libo County**, Dongting village, alt. 750 m, 11 Aug. 1982, *Song Xiang-hou & Gao Guang-wen 298*. IBSC 0116488 n.v. NF (HNIF) n.v. • **Dushan County**, Shuiyao (Sweiyao), alt. 640 m, 3 Apr. 1983, *Song Xiang-hou 1344*. NF (HNIF) n.v.

holotype image below accessed 20 Mar. 2019:

holotype (NF): http://www.docin.com/p-1050989203.html (Sima 2011: 310, photo 2–33).

isotypes (IBSC): Specimen images not uploaded to CVH at 6 Nov. 2022.

paratypes (IBSC) and (NF): specimen images not uploaded to CVH at 18 Mar. 2026.

##### Additional material.

Specimen images below accessed 17 Nov. 2022:

**Southeastern Guizhou** • Pinfa (= Pingfa), 13 June 1907, *Pierre Julien Cavalerie 3182*. P 00204446! P00204447!

(P): https://science.mnhn.fr/institution/mnhn/collection/p/item/p00204446?listIndex=64&listCount=283

(P): https://science.mnhn.fr/institution/mnhn/collection/p/item/p00204447?listIndex=65&listCount=283

superscript numbers at barcodes indicate the corresponding URL in the subsequent list:

**Guizhou** • Libo County, Maolan, 2 (6*) May 1984, *Lan Kai-min 840395*. GFS* 0011581!^1^GZAC 0016222!^2^GZAC 0016224!^3^GZAC 0016275!^4^. **ibid** • roadside, at base of mountain rock, alt. 640 m, 4 May 1984, *Lan Kai-min 840430*. GFS 0011580!^5^GZAC 0016225!^6^GZAC 0016227!^7^GZAC 0016228!^8^GZAC 0016229!^9^GZAC 0016276!^10^GZAC 0018069!^11^.

Superscript numbers against URLs match those at the appropriate barcodes listed previously.

(GFS): https://www.cvh.ac.cn/spms/detail.php?id=f8a476d0**^1^**

(GZAC): https://www.cvh.ac.cn/spms/detail.php?id=ee0be4fc^**2**^

GZAC): https://www.cvh.ac.cn/spms/detail.php?id=ee0be63d^**3**^

(GZAC): https://www.cvh.ac.cn/spms/detail.php?id=ee0c066c**^4^**

(GFS): https://www.cvh.ac.cn/spms/detail.php?id=f8a47634^**5**^

(GZAC): https://www.cvh.ac.cn/spms/detail.php?id=ee0be6dd**^6^**

(GZAC): https://www.cvh.ac.cn/spms/detail.php?id=ee0be81e^**7**^

(GZAC): https://www.cvh.ac.cn/spms/detail.php?id=ee0be8c3^**8**^

(GZAC): https://www.cvh.ac.cn/spms/detail.php?id=ee0be968**^9^**

(GZAC): https://www.cvh.ac.cn/spms/detail.php?id=ee0c070c**^10^**

(GZAC): https://www.cvh.ac.cn/spms/detail.php?id=ee104bf3**^11^**

##### Note 1.

*Manglietia calcarea* X.H. Song was made a variety of *M. fordiana* Oliv. in Chen & Nooteboom, wherein specimen *J. Cavalerie & J.J. Fortunat 2263* actually represents the type of *Michelia cavaleriei* Finet & Gagnep. from Guizhou, although one of the two isotype specimens at Kew herbarium was determined by both Dandy and subsequently by B.L. Chen in May 1990 as *Manglietia insignis*. Chen returned a month later and re-identified the same specimen as *Michelia cavaleriei*(?). There are two other labels attached to the specimen sheet at Kew incorrectly identifying this specimen as *Michelia cavaleriei*, or more plausible, either these labels or the specimen, have, at some time, been transposed. http://apps.kew.org/herbcat/getImage.do?imageBarcode=K000681457 [acc: 20 Mar. 2019]

The above Kew specimen is apparently one of the two specimens listed under M. fordiana var. calcarea in Chen and Nooteboom (1993: 1040).

##### Note 2.

*Manglietia calcarea* was made a variety of *M. aromatica* Dandy based on specimens studied at various herbaria (Sima et al. 2014: 98). However, from the differentiating features compiled in Tables 4, 5, the present authors believe *M. calcarea* to be sufficiently distinguished from both *M. aromatica* and *M. fordiana* to maintain its independent species status. Accordingly, the present authors follow the recognition of *Manglietia calcarea* as a distinct species in Flora of China (Xia et al. 2008: 59), Sima (2011: 97), Deng and Yang (2015: 45) and Yang et al. (2016: 147), and it is therefore reinstated and transferred to *Magnolia*.

##### Note 3.

Besides the differences compiled in Table 4, the distribution of *Manglietia calcarea* and *M. aromatica* are geographically isolated, with *M. calcarea* occurring from 640–820 m only in southern Guizhou, whereas *M. aromatica* occurs at higher altitudes of between 900–1400 m in six counties of Guizhou (Yang and Fang 2002: 22) and in adjacent SE Yunnan, as well as 850–1600 m in SW Guangxi and additionally the karst region of Huanjiang County, northern Guangxi (Peng 2013). *M. aromatica* is not recorded for northern Guangxi in Flora of China, as is also the case for central-southern Yunnan where it occurs somewhere between 2200–2380 m on the eastern flank of Ailao Mountain within the 46666 ha Honghe Nature Reserve in Yuanjiang County (Li 2008: 40). The more widespread *M. fordiana* compared in Table 5 occurs between 500–1300 m and is found in the previously mentioned provinces plus numerous others (Grimshaw and Bayton 2009: 490).

**Table 4.** The **26 presently known** differentiating features between *Manglietia calcarea* X.H. Song and *Manglietia aromatica* Dandy.

**Plant feature**	***Manglietia calcarea***	***Manglietia aromatica***
**life form**	**medium-sized tree to 17 m high^2^**	**very large tree to 42 m high^6^**
**trunk diameter**	**to 35 cm (to 58 cm^2^)**	**to 140 cm^9^ (to 197 cm^6^)**
leaf shape	obovate-elliptic or obovate-lanceolate	oblanceolate-oblong to oblanceolate**^9^**
leaf dimensions	17–20 × 6–8 cm. See Fig. 9, page 136	15–19 × 6–7 cm**^6,8^**
leaf apex	rounded with tapering point	short acuminate or subacuminate
leaf base	narrowly decurrent	cuneate, occasionally slightly unequal
lateral leaf vein pairs	14–17	12–16**^10^**
**petiole length**	**2.8–3.8 cm**	**1.5–2.5 cm^8^**
**stipular scars**	**4–5 mm**	**4–12 mm^10^**
**stipule indumentum**	**densely pubescent^3^**	**glabrous^8^**
flower peduncle	5–8(–20) × 5–6 mm**^1^**	ca. 12 × 4–5 mm**^5^**
**tepal number**	**9**	**11–12**
**tepal colour**	**white, lilac lower half outside**	**pale green**
outer 3 tepals shape	obovate-elliptic, apex rounded	narrowly ovate-oblong**^9^**
**outer 3 tepals dimensions**	**ca. 6.2 × 2.5 cm**	**7–11 × 3.5–5 cm^6^**
stamen length	ca. 12 mm	15–18 mm**^7^**
gynoecium shape	elliptic-ovoid	ovoid (ovoid-globose**^7^)**
**immature carpels number**	**12–16 (rarely more)**	**29–39^5^**
**ovule number**	**ca. 7^1^**	**3–4^10^**
**fruit peduncle dimensions**	**10–15 mm (ca. 9–11 × 6 mm^1^)**	**13–19 × 6–12 mm^5^**
fruit aggregate shape	ovoid-globose	subglobose or ovoid-globose
**fruit aggregate dimensions**	**ca. 6 cm long × 5 cm diameter**	**7–8 cm diameter^9^**
seed shape	oblong	broadly ellipsoid
**seed dimensions**	**ca. 8–10 × 5–6 mm**	**10–12 × 7–8 mm^5^**
flowering period	April–May**^4^** (expanding flower-bud on April 12 at Fig. 4. page 91)	May–June**^9^**
fruiting period	August–September**^4^**	September–October**^9^**

Note: The listed features of *Manglietia calcarea* are cited from Song (1984: 46), supplemented by Chen and Nooteboom (1993: 1040)**^1^**, Deng and Yang (2015: 45)**^2^**, Liu (2007)**^3^** and Sima (2011: 97)**^4^**. Those of *Manglietia aromatica* are from Dandy (1931: 231), supplemented by Chen and Nooteboom (1993: 1034)**^5^**, Deng and Yang (2015: 42)**^6^**, Fu and Jin (1992: 430)**^7^**, Law (1996: 94)**^8^**, Liu et al. (2004: 124)**^9^**, and Tiêṕ (1980: 536 or 567)**^10^**.

**Table 5.** The **25 presently known** differentiating features between *Manglietia calcarea* X.H. Song and *Manglietia fordiana* Oliv.

**Plant feature**	***Manglietia calcarea***	***Manglietia fordiana***
**life form**	**medium-sized tree to 17 m high^2^**	**medium-sized tree to 20 m high^7^ (to 22 m^8, 11^ or 25 m^9^)**
trunk diameter	**to 35 cm**	**to 45 cm^9^**
**twig indumentum**	**glabrous at all stages^1^**	**rufous pilose, later glabrous^7^**
**leaf texture**	**papery or thinly leathery in patches**	**leathery^7^**
leaf shape	obovate-elliptic or obovate-lanceolate	narrowly obovate, narrowly elliptic-obovate or oblanceolate**^7^**
leaf dimensions	to 20 × 8 cm	to 20 × 6 cm**^5^** (to 20 × 8 cm**^10^**)
leaf apex	rounded with tapering point	short-acute with obtuse mucro**^7^**
leaf base	narrowly decurrent	cuneate, decurrent along petioles**^7^**
**lateral leaf veins**	**14–17 pairs**	**8–12 pairs^6^**
petiole length	2.8–3.8 cm	1–3 cm^6^
stipular scars	4–5 mm	3–4 mm (2–6 mm**^10^**)
flower peduncle	5–8(–20) × 5–6 mm**^1^**	6–11 × 6–10 mm**^6^**
tepal colour	white, lilac lower half outside	entirely pure white
tepal shape (outer 3)	obovate-elliptic, apex rounded	oblong-elliptic
tepal dimensions	ca. 6.2 × 2.5 cm (outer 3)	6–7 × 3–4 cm**^7^** (outer 3)
stamen length	ca. 12 mm	9–12 mm**^4^**
gynoecium shape	elliptic-ovoid	ovoid to subglobose**^4^**
**immature carpels**	**12–16 (rarely more)**	**23–30^6^ (25–30^10^)**
**ovule number**	**ca. 7^1^**	**8–10^6^ (8–12^10^)**
fruit aggregate shape	ovoid-globose	ovoid
**fruit aggregate size**	**ca. 6 cm long × 5 cm diameter**	**2–5 cm long^7^**
seed shape	oblong.	ovoid or broadly ovoid
seed dimensions	ca. 8–10 × 5–6 mm	6–9 × 5–8 mm^10^
flowering period	April–May**^3^** (expanding flower-bud on April 12 at Fig. 4, page 91)	May**^7^**
**fruiting period**	**August–September^3^**	**October^7^**

Note: The listed features of *Manglietia calcarea* are cited from Song (1984: 46), supplemented by Chen and Nooteboom (1993: 1040)**^1^**, Deng and Yang (2015: 45)**^2^** and Sima (2011: 97)**^3^**. Those of *M. fordiana* are from Oliver (1891: Pl. 1953), supplemented by Chen and Nooteboom (1993: 1039)**^4^**, Dandy (1928c: 127)**^5^**, Law (1996: 105)**^6^**, Liu et al. (2004: 138)**^7^**, Peng (2008: 84)**^8^**, Sutton (2022)^9^, Tiêṕ (1980: 538)**^10^** and Yang et al. (2016: 161)**^11^**

##### Note 4.

Recent genomic research sequencing the complete or nearly complete plastomes of four *Manglietia* species determined the chloroplast (cp) genome sizes of 160,074 bp for *M. fordiana* and 157,093 bp for M. calcarea*, and that they were located in different clades (Li et al. 2024). This provides additional evidence for their independence. [*Note: There is a possibility that two previously published genomic sizes for *M. calcarea* that are listed in Table 1 of this 2024 paper that are closer to the genomic size for *M. fordiana*, and even identical to one of the two *M. aromatica* accessions listed, were derived from misidentified or mislabelled specimen material. These have been frequently encountered in herbarium collections by the present authors, often due to confusion resulting from unsubstantiated synonymy!]. Another recently published research paper provided a phylogenetic insight into the segregate genus *Manglietia* consisting of 31 species in four highly supported clades, as illustrated in two phylogenetic trees (fig. 11, Luo et al. 2025). This reveals that while both *M. fordiana* and *M. calcarea* are located in Clade 1 of the two phylogenetic trees, they are on sub-branches of different branches and therefore not closely related.

##### Note 5.

According to the literature searched by the present authors in Australia and overseas for a future paper, *Manglietia calcarea* is recorded only for the 21100 ha Maolan National Nature Reserve in Guizhou Province, for which *M. aromatica* is also recorded, with both of these species being found to have good application prospects for cultivation in gardens (Zhang et al. 2022). *M. calcarea* is noted in *ex situ* conservation at four botanical gardens.

##### Conservation status.

*Manglietia calcarea* X. H. Song was noted in 2018 as “not yet assessed” on the IUCN Red List of Threatened Species. Four years later, it could no longer be located there under either its own name or as a variety or synonym of *Magnolia fordiana* (IUCN 2022), as is also the case now (IUCN 2026). However, it had previously been assessed by Chinese authorities as Vulnerable: VU (Ministry of Environmental Protection & Chinese Academy of Sciences 2014) and subsequently under IUCN Red List criteria as VU D2 (Qin et al. 2017: 725). *Manglietia calcarea* possibly justifies an endangered classification, having been previously recorded as Endangered 28 years ago (Zou 2001: 48; Huang and Zou 2002: 172) and again a decade later (Linghu et al. 2012), as had also occurred for the sporadic but more widely distributed *Manglietia aromatica* which was classified as Endangered 34 years ago (Jin et al. 1992: 430) and is presently classified as Endangered C2a(i) on the IUCN Red List of Threatened Species (IUCN 2026).

##### Recommendation.

An IUCN assessment of the current conservation status in the wild of *Magnolia calcarea* (syn. *Manglietia calcarea*) needs to be undertaken at the earliest opportunity! This should include not only the Maolan National Nature Reserve and adjacent regions of Libo County, Guizhou, but inquiries could also be made regarding an apparent outlying occurrence at Pingfa in Guiding County where *J. Cavalerie 3182*, collected 13 June 1907 and originally identified as *Manglietia fordiana* (Léveillé 1914–1915: 269), was subsequently determined by Chen Bao-liang in 1990 as M. fordiana var. calcarea, under which name it is included in Chen and Nooteboom (1993: 1040).

#### Magnolia calcicola

Taxon classificationPlantaeMagnolialesMagnoliaceae

(C.Y. Wu ex Y.H Liu & R.F. Wu) C.B.Callaghan & S.K.Png comb. nov.

0C7176A9-BD4C-50C8-AF04-F5F1B8A163AE

urn:lsid:ipni.org:names:77382807-1

**Figure 5.**
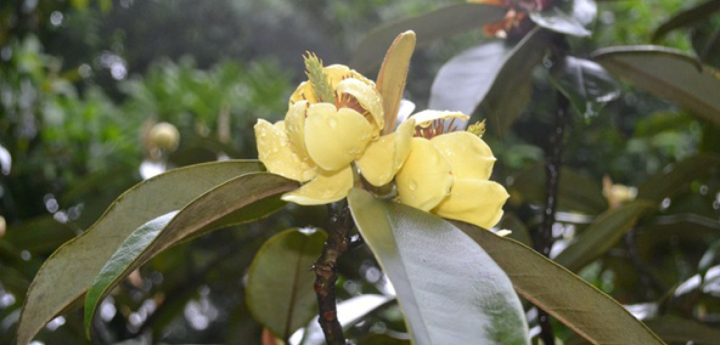
*Magnolia calcicola* (*Michelia calcicola*) showing its yellow flowers – photo taken by the authors at Kunming Botanical Garden, Yunnan on 17 April 2017. © Callaghan & Png (ABA).

Tables 6, 7, Figs 1, 5

Michelia calcicola C.Y. Wu ex Law & Y.F. Wu, Acta Botanica Yunnanica 10(3): 339, fig. 5: 9–16 (Liu & Wu [Law and Wu 1988]). **[Basionym]**.
Michelia ingrata B.L. Chen & S.C. Yang, in Annals of Missouri Botanical Garden 80(4): 1067 (Chen and Nooteboom 1993) and World Checklist and Bibliography of Magnoliaceae: 57 (Frodin and Govaerts 1996), both p.p. quoad syn. Michelia calcicola C.Y. Wu ex Law & Y.F. Wu.
Michelia fulva Hung T. Chang & B.L. Chen, in Acta Botanica Yunnanica 19(2): 137 (Li 1997) and Proceedings of the Sixth National Scientific Conference on the Ecology and Biological Resources, Hanoi, Vietnam: 134 (Nguyễn et al. 2015), both p.p. quoad syn. Michelia calcicola C.Y. Wu ex Law & Y.F. Wu—Flora of China Vol. 7: 81 (Xia et al. 2008), p.p. quoad syns. Michelia calcicola C.Y. Wu ex Law & Y.F. Wu and Michelia fulva var. calcicola (C.Y. Wu ex Law & Y.F. Wu) Sima & Hong Yu.
Magnolia fulva (Hung T. Chang & B.L. Chen) Figlar, online at World Checklist of Magnoliaceae (Govaerts 2003), p.p. quoad syn. Michelia calcicola C.Y. Wu ex Law & Y.F. Wu.
Michelia fulva var. calcicola (C.Y. Wu ex Law & Y.F. Wu) Sima & Hong Yu, in Seed Plants of Honghe Region in SE Yunnan, China: 55 (Shui 2003), Vouchered Flora of Southeast Yunnan, Vol. 1: 57 (Sima and Lu 2009), A Taxonomic Revision of the Magnoliaceae from China: 186 (Sima 2011) and Magnoliaceae Plants of Guizhou: 118 (Deng and Yang 2015), each p.p. quoad syn. Michelia calcicola C.Y. Wu ex Law & Y.F. Wu.

##### Present Chinese names.

石灰含笑 or 灰岩含笑 meaning “lime michelia or limestone michelia”, and also 钙土含笑 meaning “calcareous soil michelia”.

##### Proposed Chinese name.

灰岩木兰 meaning “limestone magnolia”.

##### Type.

China. **Yunnan Province** • Guangnan County, alt. 1500 m, on rock hill, 11 Mar. 1940, fl, *Wang Chi-wu, Zhang Ying-bo, Liu Ying 87716*. holotype: KUN 41631! Isotypes: IBSC 0053097! PE 00103787!

**Paratypes**. China. **Yunnan Province** • Malipo County, Tien-chang, alt. 1200 m, in wooded valley, occasional, 8 m tree, 20 Feb. 1940, *Wang Chi-wu 87033*. IBSC 0053094 n.v. IBSC 0053095 n.v. KUN 41642 n.v. PE 00103784! WUK 0274842 n.v. **ibid**. • alt. 1800–2000 m, 4.5 m tree with shrubby habit, rare, in open thickets on rock, 3 Nov. 1947, *Feng Kuo-mei 12844*. KUN 41632 n.v. PE 00934131! PE 00934132**! Xichou County** • Shi Shan, mountain-top forest, *C.A. Wu* (*Wu Quan-an*) *8019*. KUN? n.v. – **Guangxi Zhuang Autonomous Region** • Longjin (Longzhou) County, Jingang Shan, alt. 590 m, Jan. 1965, *Chen Shao-qing* (*S.H. Chun*) *13315*. IBSC 0053093 n.v.

**Table 6.** The **29 presently known** differentiating features between *Michelia calcicola* C.Y. Wu ex Law & Y.F. Wu *and Michelia fulva* Hung T. Chang & B.L. Chen.

**Plant feature**	***Michelia calcicola***	***Michelia fulva***
**life form**	**small-sized tree to 8 m (paratype)**	**medium-sized tree to ca. 15 m (to 16 m^2^)**
**colour of twigs**	**dark brown**	**greyish-white^2^**
twig indumentum	initially pale yellow tomentose, then glabrous	fulvo tomentose (coppery or rufous tomentose**^2^**)
bud indumentum	yellowish-brown tomentose**^1^**	gold-flecked velutinus
leaf texture	leathery.	thickly leathery
leaf shape	oblong or oblong-ovate	elliptic or ovate-elliptic
**leaf dimensions**	**13–18 × 4.5–7 cm**	**ca. 18–23.5 × 8–10 cm**
leaf apex	acuminate or acute	obtuse or subacute
leaf base	cuneate or rounded	rounded or broadly obtuse
leaf indumentum or not adaxially	appressed villose when young, then glabrous	glabrous.
young leaf abaxial indumentum	appressed yellow-brown villose when young, then glabrous	coppery turning grey tomentellous, then glabrescent**^2^**
**lateral leaf vein pairs**	**13–17**	**9–11**
stipules to petiole	fully parted	joined for approx. lower quarter
**petiole length**	**1.5–3 cm**	**3.2–4.5 cm**
flower peduncle indumentum	densely yellow long tomentose	yellowish-brown velutinous
**tepal number**	**9**	**13 (13–17 ^2^)**
tepal colour	yellow (see Figs 1, p. 80 and 5, p.95)	: red (white, fading to yellow**^2^**)
**tepal dimensions**	**4–4.5 × 1–1.5 cm**	**external tepals 4.7–5.3 × 2.6–3 cm internal tepals 4.3–4.7 × 1.6–2 cm**
**stamen length**	**20–24 mm**	**33–38 mm**
**anther length**	**15–17 mm**	**25–28 mm**
**gynoecium length**	**ca. 2 cm, exserted past androecium**	**ca. 2.4 cm, incl. within androecium**
immature carpels	ovate	narrowly ovoid
**immature carpels**	**glabrous**.	**densely villose**
mature carpels shape	oblong.	ovoid or broadly ovate
**mature carpels size**	**15–17 x 10 mm**	**ca. 10–15 × 9–10 mm**
flowering period	March**^1^** (April, Fig. 1 p.80; Fig. 5, p. 95) [China]	April [China]**^2^**
fruiting period	November [China]**^1^**	Sept.–Oct. [China**]^2^** Oct.–Nov. [Vietnam]**^3^**
altitude	590–1500 m	1690–1950 m [China]**^2^** 1100–1600 m [Vietnam]**^4^**
distribution.	SE Yunnan, Guangxi**^1^**	Maguan, SE Yunnan**^2^** Vietnam**^4^**

Note: The listed features of *Michelia calcicola* are from Liu[Law] and Wu (1988: 339), and Liu et al. (2004: 226)**^1^**. Those of *Michelia fulva* are from Chang and Chen in Chen (1987: 87), supplemented by Liu et al. (2004: 264)**^2^**, Vũ and Xia (2010)**^3^** and the Vietnam Plant Database (2022)^4^.

**Table 7.** The **25 presently known** differentiating features between *Michelia calcicola* C.Y.Wu ex Law & Y.F. Wu and *Michelia ingrata* B.L. Chen & S.C. Yang.

**Plant feature**	***Michelia calcicola***.	***Michelia ingrata***.
**life form**	**small-sized tree to 8 m high**	**medium-sized tree to 16 m high**
colour of twigs	dark brown	pale brown**^2^**
indumentum of twigs	initially pale yellow tomentose, then glabrous	densely pale brown villose in first year, then glabrescent
leaf texture	leathery.	thickly leathery
leaf shape	oblong or oblong-ovate	elliptic or obovate-elliptic
**leaf dimensions**	**13–18 × 4.5–7 cm**	**19–24 × 7.7–9.4 cm**
leaf apex	acuminate or acute	short acute (mucronate**^2^**)
leaf base	cuneate or rounded	broadly cuneate
leaf indumentum adaxial	appressed villose initially, then glabrous	glabrous.
young leaf indumentum abaxial	appressed yellow-brown villose when young, then glabrous	glabrous or pale brown villose on midribs and margins**^2^**
**lateral leaf vein pairs**	**13–17**	**9–13**
petiole length	1.5–3 cm	2.8–4.2 cm
**flower peduncle length**	**ca. 1 cm**	**3–4 cm**
flower peduncle indument.	densely yellow long tomentose	densely pale brown villose**^2^**
**tepal number**	**9**	**11–12**
tepal colour	yellow^1^ (see Figs 1, p.80 and 5, p.95)	pale yellow (golden**^2^**)
tepal dimensions	4–4.5 × 1–1.5 cm	outer 3: ca. 5.7 × 3.5 cm inner 8–9: 4–4.9 × 1.9–2.1 cm
stamen length	20–24 mm	17–22 mm
**flower fragrance**	**pleasant**.	**unpleasant, as alluded by the name**
gynoecium indumentum	near glabrous: see gynoecium Fig. 1* p.80	tomentose.
gynoecium / androecium	exserted well beyond androecium*	exserted slightly from androecium
immature carpels shape	ovate.	ovoid**^2^**
**fruiting period**	**November^1^**	**September–October^2^**
altitude.	590–1500 m^1^	ca. 1600 m^2^
distribution.	SE Yunnan, Guangxi**^1^**	SE Yunnan**^2^**

Note: The listed features of *Michelia calcicola* are from Liu[Law] and Wu (1988: 339), supplemented by Liu et al. (2004: 226)**^1^**. Those of *Michelia ingrata* are from Chen and Yang (1988: 95), who record the fruit as then unknown, supplemented by Liu et al. (2004: 276)**^2^**.

Specimen image below accessed 28 Jan. 2023

(KUN): https://www.cvh.ac.cn/spms/detail.php?id=7b6d96ed

Specimen image below accessed 20 Mar. 2019:

isotype (IBSC): http://www.docin.com/p-1050989203.html (Sima 2011: 316, photo 2–57).

paratypes (KUN, WUK): specimen images not uploaded to CVH at 18 Mar. 2026.

Specimen images below accessed 20 Mar. 2019; re-accessed with new URLs 22 Oct. 2022:

isotype (PE): https://www.cvh.ac.cn/spms/detail.php?id=01e6acaf

paratype (PE): https://www.cvh.ac.cn/spms/detail.php?id=01e6ae5a

paratype (PE): https://www.cvh.ac.cn/spms/detail.php?id=f00fd342

paratype (PE): https://www.cvh.ac.cn/spms/detail.php?id=f00fd3cb

##### Additional material.

China. **Yunnan Province** • Guangnan County, Mu-nei, alt. 1550 m, rock hill, ca. 4.6 m high, abundant, 13 Mar. 1940, *Wang Chi-wu & Liu Ying 87811*. PE 00103786!* PE 00934099! [*misapplied label at top L-H corner has T.T. Yu as collector]. • Malipo Co., Hwang-jin-in, alt. 1200–1600 m, tree 18.2 m, in mixed forests on rock mount, 14 Nov. 1947, *Feng Kuo-mei 13274*. PE 00103785!

Specimen images below accessed 30 Nov. 2022:

(PE): https://www.cvh.ac.cn/spms/detail.php?id=01e6adca

(PE): https://www.cvh.ac.cn/spms/detail.php?id=f00fe53e

(PE): https://www.cvh.ac.cn/spms/detail.php?id=01e6ad3a

**Yunnan Province** • Maguan County, alt. 1800 m, May 1982, *Liu Yu-hu & Zhou Ren-Zhang 7029*. IBSC 0053096 n.v. • 9 Sept. 1986, *Chen Bao-liang 86S-047*. SYS sys00050882 n.v. • 1500 m, Oct. 1986, *Chen Bao-liang 86S-539*. SYS sys00050880 n.v. • 2050 m, Nov. 1986, *Chen Bao-liang* s.n. SYS sys00050881 n.v. Specimen images not uploaded to CVH at 18 Mar. 2026.

##### Note 1.

There is an apparent error in Flora of China Vol. 7 (Xia et al. 2008: 81), where a synonym of *Michelia fulva* Hung T. Chang & B.L. Chen is listed as M. fulva var. calcicola (C.Y. Wu ex Y. H. [sic] Law & Y.F. Wu) Sima & Hong Yu. As the preceding taxon in the list of synonyms is *Magnolia fulva*, the logical conclusion is that the synonym in question represents Magnolia fulva var. calcicola, as it is, in fact, listed in the Index to Scientific Names (Xia et al. 2008: 488). A check of the Magnoliaceae of China dissertation (Sima 2011: 186), reveals it in fact to be Michelia fulva var. calcicola (C.Y. Wu ex Law & Y.F. Wu) Sima & Hong Yu (from Shui 2003: 55). However, as shown in Tables 6, 7, *M. calcicola* is sufficiently distinct from *M. fulva* and also *M. ingrata* B.L. Chen & S.C. Yang under which it was placed in synonymy in Chen and Nooteboom (1993), to maintain its species status. Sima (2011: 326), illustrates the contrasting difference between the indumentum of the undersides of the leaves of M. fulva var. calcicola at plate 3–3G and that of the leaves of *M. fulva* at plate 3–3F. All things considered, *M. calcicola* is a well-defined species, as is recognised in Law (1996: 175), Liu et al. (2004: 226), Xing et al. (2009: 202) and Yang et al. (2016: 235), and it is therefore reinstated and transferred to *Magnolia*.

##### Note 2.

A search of the literature for a future paper has found *Michelia calcicola* recorded for two nature reserves and in *ex situ* conservation at three botanical gardens. These include South China Botanical Garden, Guangzhou, Guangdong Province, in which *Michelia calcicola*, *M. fulva* and *M. ingrata* are recognised as separate species growing in the Magnolia Garden.

##### Conservation status.

*Magnolia ingrata* (B.L. Chen & S.C. Yang) Figlar, syn. *Michelia ingrata* under which *M. calcicola* was originally placed in synonymy, was assessed by the China Expert Workshop as Endangered EN B1ab(i,ii,iii)+B2ab(i,ii,iii), as recorded in The Red List of Magnoliaceae (Cicuzza et al. 2007: 26). While *Michelia calcicola* does not appear on the current IUCN Red List of Threatened Species (IUCN 2026), it was previously noted there in 2018 as “not yet assessed”. Vietnamese researchers (Nguyễn et al. 2015: 134) record that *Michelia fulva* (including *M. calcicola* as a synonym) occurs in at least two natural locations in China and four in Vietnam and recommend that this species should be classified as Endangered EN B2ab (i–iii, v). Subsequently, Chinese authorities utilising IUCN criteria determined *Michelia fulva* as Endangered, EN A2c; D (Qin et al. 2017: 725). Therefore, *Michelia calcicola*, once again independent from *Michelia ingrata* and *Michelia fulva*, would be expected to be at an equal or higher level of endangerment, and in fact *M. calcicola* had been assessed more than a decade earlier by Chinese authorities utilising IUCN Red List criteria as Endangered, EN A2c (Wang and Xie 2004: 328).

##### Recommendation.

In view of the above assessment made 22 years ago, an IUCN evaluation of the current conservation status in the wild of *Magnolia calcicola* (syn. *Michelia calcicola*) disengaged from *Magnolia fulva* (syn: *Michelia fulva*) and independent of *Magnolia ingrata* (syn. *Michelia ingrata*) needs to be undertaken at the earliest possible opportunity!

#### Magnolia chingii

Taxon classificationPlantaeMagnolialesMagnoliaceae

(W.C. Cheng) C.B. Callaghan & S.K. Png comb. nov.

F133D25F-B2AB-5375-96B1-EED3DE9D26CA

urn:lsid:ipni.org:names:77382805-1

Table 8

Michelia chingii W.C. Cheng, Contributions from the Biological Laboratory of the Science Society of China, Botanical Series 10(2): 110–111 (Chien et al. 1936). **[Basionym]**.
Michelia maudiae Dunn, in Annals of Missouri Botanical Garden 80(4): 1072 (Chen and Nooteboom 1993), World Checklist and Bibliography of Magnoliaceae: 58 (Frodin and Govaerts 1996), Flora of China Vol. 7: 83 (Xia et al. 2008), A Taxonomic Revision of the Magnoliaceae from China: 221 (Sima 2011) and Magnoliaceae Plants of Guizhou: 157 (Deng and Yang (2015), each p.p. quoad syn. Michelia chingii W.C. Cheng.
Magnolia maudiae (Dunn) Figlar, online at World Checklist of Magnoliaceae (Govaerts 2003), and in New Trees – Recent Introductions to Cultivation: 499 (Grimshaw and Bayton 2009), both p.p. quoad syn. Michelia chingii W.C. Cheng.

##### Present Chinese name.

秦仁昌含笑 meaning “Ching Ren-chang’s michelia”.

**Table 8.** The **14 presently known** differentiating features between *Michelia chingii* W.C. Cheng and *Michelia maudiae* Dunn.

**Plant feature**	***Michelia chingii***	***Michelia maudiae***
**tree height**	**common in woods, to max. 15 m**	**to 31 m^2^**
**branchlet colour**	**yellow-brown**	**green becoming grey, initially covered with white powder**
leaf shape	oblong-lanceolate, oblong or oblanceolate	oblong-elliptic, rarely ovate-elliptic**^4^**
leaf dimensions	16–19 × 5.7–7.7 cm	7–18 × 3.5–8.5 cm**^4^**
leaf apex	obtuse, short-acuminate or obtuse-acute	abruptly short-acuminate
leaf texture	thinly leathery	leathery.
**lateral leaf veins**	**ca. 12–14 pairs**	**7–12 pairs**
**leaf abaxially**	**glaucescent, covered in minute blunt hairs**	**greyish-green, glabrous, covered in white powder^3^**
**tepal number**	**apparently up to 12^1^ (see note 2 of text)**	**9^2,3,4,7^**
**ovules in carpel**	**ca. 6**	**6–14^1^**
**fruit peduncle**	**1.8–2.4 cm long**	**4 cm long^5^**
**fruit aggregates**	**6.5–8 cm long**	**7–15 cm long^2,3,4,7^**
**mature carpels**	**sessile (non-stalked)**	**stalked (fig. 7^4^)**
fruiting period	August (–September?)	September–October**^2, 3^**–November**^6^**

Note: The distinguishing features of *Michelia chingii* are from Cheng in Chien et al. (1936: 110), supplemented by Chen and Nooteboom (1993: 1072)^1^ with those of *Michelia maudiae* from Dunn (1908: 353), supplemented by Chen and Nooteboom (1993: 1072)^1^, Deng and Yang (2015: 157)^2^, Law (1996: 179)^3^, Liu et al. (2004: 290)^4^, Lee (1935: 487)^5^, Yang et al. (2016: 297)^6^ and Xing et al. (2009: 209)^7^.

##### Proposed Chinese name.

秦仁昌木兰 meaning “Ching Ren-chang’s magnolia”.

##### Type.

China. **Chekiang (= Zhejiang) Province** • Lungtsuan (= Longquan), region of King Yuan (= Qingyuan), Mao Shan, alt. 1158 m (common tree above 610 m), in woods of mostly evergreens, tree 13.7 m, 24 Aug. 1924, fr, *Ching Ren-chang 2452*. holotype: PE 00934130! isotypes: A 00039042! E 00035678! K n.v. US 00104084!

Specimen image below accessed 1 Apr. 2019; re-accessed with new URL 22 Oct. 2022:

holotype (PE ex HSBL): https://www.cvh.ac.cn/spms/detail.php?id=f00fd454

Specimen images below accessed 1 Apr. 2019:

isotype (A): https://kiki.huh.harvard.edu/databases/specimen_search.php?mode=details&id=1038

isotype (E. ex US): http://data.rbge.org.uk/herb/E00035678

isotype (US): https://collections.nmnh.si.edu/search/botany/search.php?action=11&irn=10119110

##### Note 1.

*Michelia chingii* is recorded as a synonym of Magnolia (Michelia) maudiae by the above-listed authors, but differs sufficiently from the latter species in its morphological characteristics, as compiled in Table 8, to justify its species status and is therefore reinstated and transferred to *Magnolia*.

##### Note 2.

While the flowers of *Michelia chingii* were unavailable for W.C. Cheng to describe, it would appear that the number of tepals for this species is up to 12. This is because the flowers of *Michelia maudiae* bear 9 tepals as referenced in Flora of China, Magnolias of China and by others, yet it is recorded with 9–12 tepals in Chen and Nooteboom (1993: 1072). Since the only taxon listed in synonymy with *M. maudiae* by the last authors is *M. chingii*, it is probable that the higher number of tepals cited has been derived by one of these authors counting the scars left by the fallen tepals of a *M. chingii* specimen.

##### Conservation status.

*Michelia chingii* is noted on the IUCN Red List of Threatened Species as a synonym of *M. maudiae* which is recorded as of “Least Concern” (IUCN 2026).

##### Recommendation.

In view of the confirmation of *Michelia chingii* as an independent species, its conservation status should be assessed for the IUCN Red List at the earliest opportunity! A starting point would be the forests above 610 m on Mao Shan in Qingyuan County of southwestern Zhejiang, perhaps in the region of the 1,333 ha Baishanzu Nature Reserve before its amalgamation with the 4,667 ha Fengyang Shan Nature Reserve in Longquan County to its north. Mao Shan is where its collector R.C. Ching recorded this species, subsequently named after him, as a common tree to 15 metres in 1924. The last known collection of *Michelia chingii* in southern Zhejiang Province was 68 years ago in 1958, so hopefully it still survives, at least on Mao Shan and wasn’t completely exterminated during either the Great Leap Forward of 1958–1962 or the following Cultural Revolution of 1966–1976. The later period resulted in large tracts of the adjacent Fujian province less than 30 km away being completely denuded of timber trees as a result of six years of illegal logging (Primack 1988). Since Primack mentions that this wholesale destruction of forests occurred to a greater or lesser extent throughout China during these tumultuous years and one of the present authors has noted how it almost drove the “living fossil” *Cathaya argyrophylla* Chun & Kuang to the brink of extinction (Callaghan 2012), there is the possibility that this illegal logging has resulted in the demise of *Michelia chingii* occurring in the forests on the slopes of Mao Shan between 610 and about 1160 metres. These forests would have been more easily accessible than the remote, difficult to access *Cathaya* forests, the reason for the endangered Cathay silver fir’s relatively late discoveries in S.E. Sichuan in 1938 and Guangxi in 1955. If the *Michelia chingii* forests on Mao Shan became a victim of the Cultural Revolution, was this mountain later replanted by forestry workers with the more widely occurring *M. maudiae*?

#### Magnolia congjiangensis

Taxon classificationPlantaeMagnolialesMagnoliaceae

(Y.K. Li & X.M. Wang) C.B. Callaghan & S.K. Png comb. nov.

3A1C1AFD-3A11-5267-8F59-65F7937063E5

urn:lsid:ipni.org:names:77382806-1

Table 9

Michelia chongjiangensis Y.K. Li & X.M. Wang, Guizhou Science 1983(2): 18–19, fig. 22 (Li and Wang 1983). [see Note 5 regarding an orthographic error in the spelling of the basionym]. **[Basionym]**.
Michelia leveilleana Dandy, in World Checklist and Bibliography of Magnoliaceae: 58 (Frodin and Govaerts 1996) and Flora of China Vol. 7: 89 (Xia et al. 2008), both p.p. quoad syn. Michelia chongjiangensis Y.K. Li & X.M. Wang.
Magnolia leveilleana (Dandy) Figlar, online at World Checklist of Magnoliaceae (Govaerts 2003), p.p. quoad syn. Michelia chongjiangensis Y.K. Li & X.M. Wang.

##### Present Chinese name.

从江含笑 meaning “Congjiang michelia”, with the correct spelling for the county after which it is named.

**Table 9.** The **19 presently known** differentiating features between *Michelia chongjiangensis* Y.K. Li & X.M. Wang and *Michelia leveilleana* Dandy (ex *Michelia cavaleriei* H. Lév.).

**Plant feature**	***Michelia chongjiangensis***	***Michelia leveilleana***
**life form**	**large-sized tree to 25 m high^1^**	**medium-sized tree to 15 m high^3^**
**trunk diameter**	**78 cm^1^**	**30 cm^3^**
indumentum of buds	appressed ferrugineous-sericeous pubescence	long persistent rufo-aureo (rufous tomentose**^2^**)
indumentum of twigs	glabrous	appressed tomentellous when young**^2^**
leaf shape	oblong-elliptic or ovate-elliptic	obovate as for the type specimen
**leaf dimensions**	**to 10 (–12.5) × 5 (–6.3) cm**	**to 15 × 5 cm**
leaf apex	acute or abruptly acute	acuminate or acute**^2^**
leaf base	rounded or sometimes obtuse	obtuse, sometimes unequal**^2^**
lateral leaf vein pairs	7–12	9–15**^2^**
leaf indumentum abaxially	sparsely ferrugineous-sericeous farinose pubescence, rarely partly glabrous	yellowish-brown tomentellous (young), glabrous at maturity
**petiole length**	**2–4 cm**	**to 2.4 cm [type specimens at P and K]**
**petiole indumentum**	**glabrous**.	**appressed pubescent (ultimately glabrescent^2^)**
**tepal number**	**9–10**	**6 (–7)**
**tepal dimensions**	**3.5–3.8 × 1.4–1.8 cm^1^**	**outer 2–3: 3.0–3.5 × 0.7–1.5 cm^2^**
**stamen length**	**0.8–1.0 cm^1^**	**1.2–1.5 cm^2^**
fruit aggregate length	3–9 cm	7–9 cm**^2^**
**fruit peduncle length**	**0.6*–*1.6 cm^1^**	**3 cm^2^**
**flowering period**	**March^1^**	**April [*Julien Cavalerie 3045*: (P)]**
region of occurrence	southeastern Guizhou, N. Guangxi	western central Guizhou

Note: The listed features of *Michelia chongjiangensis* are from Li and Wang (1983: 18), supplemented by Deng and Yang (2015: 155)**^1^**. Those of *Michelia leveilleana* are from Léveillé (1911: 459, as *Michelia cavaleriei* Lév.) [renamed as *M. leveilleana*Dandy (1927: 263)], Chen and Nooteboom (1993: 1081)**^2^** and Deng and Yang (2015: 178)^3^.

##### Proposed Chinese name.

从江木兰 meaning “Congjiang magnolia”.

##### Type.

China. **Guizhou Province** • Congjiang County, Taiyang Shan, in woods, alt. 1300 m, 1981, *Li Yong-kang 9294*. holotype: HGAS n.v. **ibid**. • 1981; *Huang De-fu 1078*. HGAS n.v.

**Paratypes**. Superscript numbers at barcodes indicate the corresponding URL in the subsequent list:

China. **Guangxi Zhuang Autonomous Region, Damiaoshan County** • Luodong town, Jiuwan Shan region, dense forests, fertile soils, mountains and valleys, alt. 1100 m, 8 m tree, 9 June 1958, fr, *Chen Shao-qing 14374*. HITBC 001704!^2^IBK 00000496!^4^IBSC 0054163!^25^KUN 41652 n.v., PE 00126135!^15^SZ n.v. WUK 0211815!^20^**ibid**. • dense forests, barren soils, mountains and hilltops, alt. 900–1150 m, 8 m tree, 17 June 1958, fr, *Chen Shao-qing 14514*. IBK 00000495!^5^IBSC 0054165 n.v. KUN 41666 n.v. NAS 00320667!^11^PE 00126126!^16^SZ n.v. WUK 0213174!^21^. **ibid**. • dense forests, mountains and hilltops, alt. 1600 m, 7 m tree, 27 Aug. 1958, fr, *Chen Shao-qing 15323*. IBK 00000498!]^6^IBSC 0054164 n.v. KUN 41674 n.v. NAS 00320668!^12^PE 00126124!^17^WUK 0211328!^22^. **ibid**. • dense forest, loamy and wet soils, hilltops, alt. 700–1000 m, 15 m tree, 17 Aug. 1958, fr, *Chen Shao-qing 15778*. HITBC 001683!^3^IBK 00000497!^7^IBSC n.v. KUN 41654 n.v. LBG 64645!^10^NAS 00320665!^13^PE 00126133!^18^SZ n.v. WUK 0212796!^23^. **ibid**. • dense forests, fertile and wet soils, mountains and hilltops, alt. 900–1200 m, 15 m tree, 17 Aug. 1958, fr, *Chen Shao-qing 16289*. IBK 00000492!^8^KUN 41656 n.v. NAS 00320666!^14^PE 00126125!^19^WUK 0211699!^24^ • **Lingui District**, dense mountain-top forest, 7 m tree, 30 June, 1952, fr, *Liang Chou-feng 30495*. GAC 0005561!^1^IBK 00000493!^9^.

holotype (HGAS): specimen image not uploaded to CVH website at 18 Mar. 2026 (see note 1 below)

Specimen images below accessed 20 Mar. 2019; re-accessed with new URLs 22 Oct. 2022:

Superscript numbers against URLs match the appropriate barcodes listed previously.

paratype (GAC): https://www.cvh.ac.cn/spms/detail.php?id=2170f245**^1^**

paratype (HITBC): https://www.cvh.ac.cn/spms/detail.php?id=e95455d9^**2**^

paratype (HITBC): https://www.cvh.ac.cn/spms/detail.php?id=e954499b**^3^**

paratype (IBK): https://www.cvh.ac.cn/spms/detail.php?id=c007a28b^**4**^

paratype (IBK): https://www.cvh.ac.cn/spms/detail.php?id=c007a1ef**^5^**

paratype (IBK): https://www.cvh.ac.cn/spms/detail.php?id=c007a3c2**^6^**

paratype (IBK): https://www.cvh.ac.cn/spms/detail.php?id=c007a327^**7**^

paratype (IBK): https://www.cvh.ac.cn/spms/detail.php?id=c007a019**^8^**

paratype (IBK): https://www.cvh.ac.cn/spms/detail.php?id=c007a0b6^**9**^

paratype (LBG): https://www.cvh.ac.cn/spms/detail.php?id=d995b0bc^**10**^

paratype (NAS): https://www.cvh.ac.cn/spms/detail.php?id=db5663aa**^11^**

paratype (NAS): https://www.cvh.ac.cn/spms/detail.php?id=db60643a**^12^**

paratype (NAS): https://www.cvh.ac.cn/spms/detail.php?id=dbf06e00^**13**^

paratype (NAS): https://www.cvh.ac.cn/spms/detail.php?id=dc0c6a42^**14**^

paratype (PE): https://www.cvh.ac.cn/spms/detail.php?id=00742929^**15**^

paratype (PE): https://www.cvh.ac.cn/spms/detail.php?id=00742d84^**16**^

paratype (PE): https://www.cvh.ac.cn/spms/detail.php?id=00742e97**^17^**

paratype (PE): https://www.cvh.ac.cn/spms/detail.php?id=479b3783**^18^**

paratype (PE): https://www.cvh.ac.cn/spms/detail.php?id=00742e0e**^19^**

paratype (WUK): https://www.cvh.ac.cn/spms/detail.php?id=bfa3efdc^**20**^

paratype (WUK): https://www.cvh.ac.cn/spms/detail.php?id=bfa3f079^**21**^

paratype (WUK): https://www.cvh.ac.cn/spms/detail.php?id=bfa3f424^**22**^

paratype (WUK): https://www.cvh.ac.cn/spms/detail.php?id=bfa3f2ec**^23^**

paratype (WUK): https://www.cvh.ac.cn/spms/detail.php?id=bfa3ee96^**24**^

Specimen image below accessed 20 Mar. 2019.

paratype (IBSC): http://www.docin.com/p-1050989203.html^**25**^ (Sima 2011: 316, photo 2–60).

Specimen images below not uploaded to CVH at 18 Nov. 2022.

IBSC 0054164; IBSC 0054165; IBSC 0054166; IBSC 0054167; KUN 41652; KUN 41666; KUN 41674; KUN 41654; KUN 41656; SZ 00164464; SZ 00164453; SZ 00164452.

##### Additional material.

(posted to CVH as *Michelia chongkiangensis*):

China. **Guizhou Province** • alt. 1400 m, 7 m tree, 8 Aug. 1959, fr, *Qiannan Team 03385*. HGAS 010738! • **Yueliang Shan**, alt. 1300 m, 15 m tree, 6 Oct. 1981, fr, *Huang De-fu 1490*. HGAS 010727**! ibid** • alt. 1420 m, mountain-top, middle of forest,14 m tree, 6 Oct. 1981, fr, *Huang De-fu 1640*. HGAS 010726! • alt. 1610 m, 12 m tree, 14 Sept. 1982, fr, *Yan Jia-mo 1174*. HGAS 010739!

Specimen images below accessed 7 Jan. 2023:

(HGAS): https://www.cvh.ac.cn/spms/detail.php?id=e4687b4b

(HGAS): https://www.cvh.ac.cn/spms/detail.php?id=e46875b4

(HGAS): https://www.cvh.ac.cn/spms/detail.php?id=e4687518

(HGAS): https://www.cvh.ac.cn/spms/detail.php?id=e4687be8

##### Note 1.

The holotype specimen for *Michelia chongjiangensis* at HGAS could not be found (Chen Xiang, pers. comm., July 2019).

##### Note 2.

*Michelia chongjiangensis* Y.K. Li & X.M. Wang is noted as a “dubious species = *Michelia ?leveilleana* Dandy” in Chen and Nooteboom (1993: 1088) under which it is then listed as a synonym in Frodin and Govaerts (1996) and in Flora of China, wherein *M. leveilleana*, described more than a century ago from central western Guizhou, is described as a tree to 15 m tall. However, *M. chongjiangensis* is recorded as a tree of 20 m or more height in a natural community in Jiaya village, Guanghui town, Congjiang County, southeastern Guizhou (Yang X.Y. et al. 2016) and to 25 m in Deng and Yang (2015: 155).

##### Note 3.

*Michelia chongjiangensis* has been accepted as an independent species by Sima in his Ph.D. dissertation on the Chinese Magnoliaceae (Sima 2011: 220, figs 4–42) and subsequently in Deng and Yang (2015: 155). In Sima’s classification system, *Michelia chongjiangensis* is placed in *Michelia* series *Metamichelia* of subsection *Michelia*, whereas *Michelia leveilleana* is placed in *Michelia* series *Dichlamys* of subsection *Micheliopsis* (Sima 2011: 204; 240). Also, Sima illustrates the contrasting difference between the indumentum of the undersurfaces of the 6–12.5 cm long leaves of *M. chongjiangensis* (Sima 2011: 325; plate 3–2H) and that of the 7.5–10.5 cm long leaves of *M. leveilleana* (Sima 2011: 326; plate 3–3I).

##### Note 4.

The length of the petioles of the leaves of the seven various type specimens of *Michelia leveilleana* (*J. Cavalerie 3045*, May 1908) at E, K, BM and P has been observed to be around the 1.7–2.2(–2.4) cm mark, whereas Li and Wang (1983) record the petiole length for *M. chongjiangensis* as 2–4 cm. Photographic images of four specimens that are deposited at HGAS that were sent to the present authors by Prof. Chen Xiang in 2013, show the noticeably long petioles of most leaves of these specimens approaching the 4 cm mark, appreciably above the length of the petioles of *M. leveilleana*.

##### Note 5.

A compilation of all the known distinguishing features of these two species is shown in Table 9, confirming the independent species status of *Michelia chongjiangensis*, which is consequently reinstated and transferred to *Magnolia* as *Magnolia congjiangensis*. The change in the spelling of the name is due to an orthographical error in the original epithet which should be ‘congjiangensis’, for Congjiang County, Guizhou, after which this species is named [ICN Art. 60.1 – “the original spelling of a name or epithet is to be retained, except for the correction of typographical or orthographical errors” (Turland et al. 2025)].

##### Note 6.

A number of the specimens of *Michelia chongjiangensis* listed previously that are posted to the Chinese Virtual Herbarium (CVH) website have been identified by various researchers as either *M. macclurei* Dandy or M. macclurei var. sublanea Dandy. However, neither Magnolias of China (Liu et al. 2004) nor the later and equally comprehensive Magnoliaceae Plants of Guizhou (Deng and Yang 2015) recognize these two taxa for Guizhou, where the type specimen of *Michelia chongjiangensis* was collected.

##### Note 7.

During research for a future paper, *Michelia chongjiangensis* has been found to occur in five nature reserves, but it is not known to be anywhere in *ex situ* cultivation.

##### Conservation status.

*Magnolia leveilleana* (Dandy) Figlar (Figlar 2000), including *Michelia chongjiangensis* as a synonym, was assessed by Rivers and Wheeler on 1 August 2012 for the IUCN Red List of Threatened Species as Data Deficient with the notation that “it may be vulnerable to extinction as it has a small and restricted distribution”, but that further information is needed to make a full assessment (IUCN 2026).

##### Recommendation.

Due to the relatively small presently known distribution of *Magnolia congjiangensis* on Taiyang Shan in Guizhou and Yueling Shan to its northwest, plus some scattered occurrences in adjacent northern Guangxi, an IUCN evaluation of the current status in the wild of *Magnolia congjiangensis* (syn. *Michelia chongjiangensis*), needs to be undertaken as soon as possible! Also, the actual present *ex situ* conservation status of *Magnolia leveilleana* (syn. *Michelia leveilleana*), minus the attached synonyms, needs urgent assessment to see how close it is to the IUCN conclusion that it may be facing extinction, when one recalls the observation in Chen and Nooteboom (1993) cited previously in Part 1 under *Magnolia pilosa* (*Michelia longipetiolata*), regarding *Michelia leveilleana*, that “after its publication by Dandy” (in 1927), “this species was never mentioned again in the literature”.

#### Magnolia floribunda var. lanea

Taxon classificationPlantaeMagnolialesMagnoliaceae

(Y.K. Sima) C.B. Callaghan & S.K. Png comb. nov.

879E05AD-5C2D-5999-8E5B-767027EE7E43

urn:lsid:ipni.org:names:77382992-1

Michelia floribunda var. lanea Y.K. Sima, Seed Plants of Mount Xilong, the Highest Mountain in South Yunnan, China (Shui et al. 2016: 130). **[Basionym]**.

##### Present Chinese name.

棉毛多花含笑 meaning “cotton wool-flowered michelia”.

##### Proposed Chinese name.

棉毛多花木兰 meaning “cotton wool-flowered magnolia”.

##### Type.

China. **Yunnan Province** • Honghe Prefecture, Jinping Miao, Yao, and Dai Autonomous County, Fenshuiling National Nature Reserve, Xilong Shan, April 2004, *Sima Y.K.2004040*. holotype: YAF n.v.

**Paratype**. **ibid**. • 2 March 2006, *Sima Y.K.06387*YAF n.v.

Specimen images of this variety are not uploaded to CVH at 27 Jan. 2026.

##### Note.

The little-known Michelia floribunda var. lanea is here transferred to *Magnolia*. It is a small tree to 8 m height, with the undersides of the leaves covered in dense woolly hairs, one of the features that distinguishes this variety from *Michelia floribunda* Finet. & Gagnep., which reaches to 20 m height (Deng et al. 2015: 136 incl. 3 photos, 137 incl. 4 photos).

##### Conservation status.

While Michelia floribunda var. lanea occurs on Xilong Shan located within the 420,260 ha Fenshuiling National Nature Reserve in southern Yunnan, a search of the literature has shed little light on its current conservation status.

#### Magnolia forrestii

Taxon classificationPlantaeMagnolialesMagnoliaceae

(W.W. Sm. ex Dandy) C.B. Callaghan & S.K. Png comb. nov.

ED0C543E-958C-58E1-80C8-D6A793EABF24

urn:lsid:ipni.org:names:77383010-1

Table 10

Manglietia forrestii W.W. Sm. ex Dandy, Notes from the Royal Botanic Garden, Edinburgh 16(77): 126, pl. 226 (Dandy 1928c). **[Basionym]**.
Manglietia fordiana var. forrestii (W.W. Sm. ex Dandy) B.L. Chen & Noot., in Annals of Missouri Botanical Garden 80(4): 1040 (Chen and Nooteboom 1993), World Checklist and Bibliography of Magnoliaceae: 51 (Frodin and Govaerts 1996) and online at World Checklist of Magnoliaceae (Govaerts 2003), each p.p. excl. syn. Manglietia globosa Hung T. Chang.
Magnolia fordiana (Oliv.) Hu var. forrestii (W.W. Sm. ex Dandy) V.S. Kumar, Kew Bulletin 61(2): 184 (Kumar 2006).
Manglietia fordiana Oliv., in Vouchered Flora of Southeast Yunnan, Vol. 1: 23 (Sima and Lu 2009) and *A* Taxonomic Revision of the Magnoliaceae from China: 88 (Sima 2011), both p.p. quoad syn. Manglietia forrestii W.W. Sm. ex Dandy.

##### Present Chinese names.

滇桂木莲 meaning “Yunnan and Guangxi manglietia” and also 锈枝木莲 meaning “rusty-branched manglietia”.

**Table 10.** The **23 presently known** differentiating features between *Manglietia forrestii* W.W. Sm. ex Dandy and *Manglietia fordiana* Oliv.

**Plant feature**	***Manglietia forrestii***	***Manglietia fordiana***
life form	large tree to 25 m**^5^** (to 30 m**^1^**)	medium tree to 20 m**^11^** (to 22 m**^12^**, 25m^13^)
trunk diameter	to 30 cm^5^ (to 150 cm^1^)	to 45 cm^13^
bud indumentum	pubescent.	initially pilose, then glabrous**^11^**
twig indumentum	reddish appressed glossy sericeous**^7^**	initially rufous pilose, later glabrous**^11^**
leaf shape	elongate-obovate or oblanceolate-oblong or oblanceolate	narrowly obovate, narrowly elliptic-obovate or oblanceolate**^11^**
**leaf dimensions**	**usually to ca. 27 × 10.5 cm (15.6–25.5 × 5.6–10 cm^6^)**	**to 20 × 6 cm^9^ (to 20 × 8 cm^12^)**
leaf apex	acuminate or abruptly acute**^5^** (caudate-acuminate**^3^**)	short-acute with obtuse mucro**^11^**
leaf base	cuneate or obtuse	cuneate, decurrent along petioles**^11^**
leaf indumentum abaxially	initially pubescent, becoming near glabrous	sparsely rufous pilose**^11^**
**lateral leaf vein pairs**	**14–20 (12–16^2^)**	**8–12^10^**
**petiole indumentum**	**rufous appressed glossy sericeous, especially near base**	**initially pubescent, then glabrous**
tepal number	9 (–10)**^1,2,5^**	9**^10^**
**stamen-bearing receptacle length**	**0.5–1.9 cm^6^**	**1.9–2.54 cm^8^**
fruit aggregates shape	ovoid or ovoid-globose	ovoid
fruit aggregates lengths	4–6 cm**^5^**	to 3.5 cm**^9^**(2–5 cm**^11^**)
carpel numbers	immature: 25–34	immature: 25–30
**carpel dimensions**	**immature: 4–4.5 × 3.5–4 mm**	**immature: 3–4 × 2.5–3 mm**
ovules per carpel	9–12**^7^**	8–12**^14^**
**mature carpels length**	**3.4–6.2 cm**	**3–4.5 cm^14^**
**mature carpel surface**	**smooth.^7^**	**tuberculate**.
**seed colour**	**black ^1,2^**	**red^10^**
**altitude range**	**1100–2900 m^5^**	**to 1200 m^10^(400–2000 m)^11^**
**distribution**.	**SW Guangxi, S and W Yunnan^5^, plus Guizhou provinces^4^, China. northern Vietnam^6^**	**Fujian, Guangdong, Hong Kong, Guangxi, Guizhou, Hunan, Jiangxi, Yunnan provinces, China^11^**

Note: *Manglietia forrestii* details are from W.W. Smith MS in Dandy (1928c: 126), plus Deng and Yang (2015: 35)**^1^**, Law (1996: 103)**^2^**, Lee (1935)**^3^**, Li et al. (2022)^4^, Liu et al. (2004: 140)**^5^**, Vũ and Xia (2011)^6^ and Tiêṕ (1980: 541)**^7^**. *M. fordiana* are derived from Oliver (1891)**^8^**, plus Dandy (1928c: 127)**^9^**, Law (1996: 105)**^10^**, Liu et al. (2004: 138)**^11^**, Peng (2008: 84)^12^, Sutton (2022)^13^ and Tiêṕ (1980: 538)**^14^**.

##### Proposed Chinese name.

滇桂木兰 meaning “Yunnan and Guangxi magnolia”.

##### Type.

China. **Mid-western Yunnan** • hills 3 days south of Tengyueh (=Tengchong), 24°25'N, 98°33'E. alt. 2133–2438 m, tree 9–15 m, in mixed forests on the hills, June 1925, fl, fr, *George Forrest 26705A*. holotype: E 00117070! isotypes: A 00039034! K 000681417! NY 00320702! PE 00934117! ex E (Forrest mentions this tree as “very close to No. 26705” which was later determined as *Manglietia hookeri* Cubitt & W.W. Sm.).

**Paratypes**. China. **Mid-western Yunnan** • hills NW of Tengyueh, 25°30'N, 98°30'E, alt. 1800–2100 m, tree 15–18 m, in mixed forests, June 1925, fl, *George Forrest 26694*. E 00117069 n.v. IBSC 0000642 n.v. PE 00934118! (ex E). **ibid**. • 25°20'N, 98°15'E, alt. 2100 m, tree 9–15 m, widely branched in deciduous forests, Oct. 1925, *George Forrest 27300*. E 00117068 n.v. SYS 00051642 n.v. **Southern Yunnan** • Szemao, alt. 1500 m, tree of 8 m, in forests, fl, *Augustine Henry 11988*. LE 01013052! LE 01013053!

Specimen images below accessed 20 Mar. 2019:

holotype (E): http://data.rbge.org.uk/herb/E00117070

isotype (A): http://kiki.huh.harvard.edu/databases/image.php?id=304810

isotype (IBSC): http://www.docin.com/p-1050989203.html (Sima 2011: 310, photo 2–35).

isotype (K): http://apps.kew.org/herbcat/getImage.do?imageBarcode=K000681417

isotype (NY): http://sweetgum.nybg.org/science/vh/specimen_details.php?irn=667267

Specimen images below accessed 20 Mar. 2019; re-accessed with new URLs 17 Oct. 2022:

isotype (PE ex E) https://www.cvh.ac.cn/spms/detail.php?id=f00fdae9

paratype (PE ex E) https://www.cvh.ac.cn/spms/detail.php?id=f00fdb72

Specimen images below accessed 20 Mar. 2019:

paratype (LE): https://en.herbariumle.ru/?t=occ&id=23638

paratype (LE): https://en.herbariumle.ru/?t=occ&id=23639

##### Additional material.

China. **Western Yunnan** • locality not noted, 1931, *George Forrest 29747*. PE 00103444! Specimen image accessed 6 Feb. 2026: (PE ex E): https://www.cvh.ac.cn/spms/detail.php?id=08af691f

##### Note 1.

*Manglietia forrestii* was made a variety of *M. fordiana* Oliv. in Chen and Nooteboom (1993) and noted to differ from *M. fordiana* on account of its larger leaves and numerous parts being brown pubescent. It was subsequently made a synonym of *M. fordiana* by the authors listed previously. However, *M. forrestii* is recognised as a distinct species in Tiêṕ (1980: 541), Law (1996: 103), Liu et al. (2004: 140), Xia et al. (2008: 59), Xing et al. (2009: 194), Deng and Yang (2015: 35) and Yang et al. (2016: 163). The present authors are in agreement with the retention of *M. forrestii* as an authentic species, due to its numerous distinguishing characteristics when compared with *M. fordiana*, as shown in Table 10.

##### Note 2.

In a pre-publication in 2010 and two studies published in 2011, *Manglietia forrestii* was confirmed as a distinct, independent species on the basis of DNA evidence (Li et al. 2010: 193–194; Xiao et al. 2011a: 489, 2011b: 2178), and the view that *M. forrestii* was a variant of *M. fordiana* was not supported (ibid. p. 2182). A later published study of the genetic diversity of 48 wild species of Magnoliaceae in Yunnan Province utilising ISSR molecular markers showed *Manglietia forrestii* not closely related to *Manglietia fordiana* on the accompanying UPGMA cluster map (Wang et al. 2020: 281, fig. 2). In view of the overwhelming evidence that *Manglietia forrestii* is a genuine species, it is reinstated and transferred to *Magnolia*.

##### Note 3.

Previously recorded as occurring in southern and western Yunnan plus south-western Guangxi (Liu et al. 2004), *Manglietia forrestii* was subsequently recorded for Vietnam (Vũ and Xia 2010) and has been more recently recorded for Guizhou Province under its antiquated name M. fordiana var. forrestii (Li et al. 2022).

##### Note 4.

According to the literature searched by the present authors in Australia and overseas for a subsequent paper, *Manglietia forrestii* is recorded in China for 14 nature reserves, as well as being recorded as occurring in *ex situ* conservation at two forest research institutions and eight botanical gardens or arboreta, including South China Botanical Garden, Guangzhou, Guangdong, where *M. fordiana* also occurs.

##### Conservation status.

Magnolia fordiana var. forrestii was noted as “Vulnerable B1 +2c” on the IUCN Red List of Threatened Species (IUCN 2026), based on a January 1998 assessment by W. Sun and hence this would have been its *in situ* conservation status 28 years ago. Since then, *Manglietia forrestii* has been assessed by Chinese authorities using IUCN criteria as VU A2c (Wang and Xie 2004: 327) and subsequently as VU A2acd (Qin et al. 2017: 725). In the intervening years, Magnolia fordiana var. forrestii was recorded as Near Threatened in The Red List of Magnoliaceae (Cicuzza et al. 2007: 17).

##### Recommendation.

Due to the uncertainty of the conservation status of Magnolia fordiana var. forrestii (*Manglietia forrestii*), as is evident above, an IUCN reassessment of the current status in the wild of *Magnolia forrestii*, which is unquestionably a genuine species, should be undertaken at the earliest opportunity!

#### Magnolia fulgens

Taxon classificationPlantaeMagnolialesMagnoliaceae

(Dandy) C.B. Callaghan & S.K. Png comb. nov.

174AB081-306C-546B-9FB9-BAC00723B4B9

urn:lsid:ipni.org:names:77383028-1

Table 11, Fig. 2

Michelia fulgens Dandy, The Journal of Botany: British and Foreign 68(7): 210–211 (Dandy 1930). **[Basionym]**.
Michelia foveolata Merr. ex Dandy, in Annals of Missouri Botanical Garden 80(4): 1066 (Chen and Nooteboom 1993), p.p. quoad syn. Michelia fulgens Dandy plus specimens *F.A. McClure 712*; *768*—World Checklist and Bibliography of Magnoliaceae: 57 (Frodin and Govaerts 1996), Acta Botanica Yunnanica 19(2): 134 (Li 1997), Flora Yunnanica. Tomus 6 (Spermatophyta): 53 (Wu and Chen 2006), Flora of China Vol. 7: 86 (Xia et al. 2008), Vouchered Flora of Southeast Yunnan, *Vol. 1*: 55 (Sima and Lu 2009), A Taxonomic Revision of the Magnoliaceae from China: 216 (Sima 2011) and Magnoliaceae Plants of Guizhou: 152 (Deng and Yang 2015), each p.p. quoad syn. Michelia fulgens Dandy.
Magnolia foveolata (Merr. ex Dandy) Figlar, in Yunnan Forestry Science and Technology 2001(2): 32 (Sima 2001), and online at World Checklist of Magnoliaceae (Govaerts 2003), both p.p. quoad syn. Michelia fulgens Dandy.

##### Present Chinese names.

亮叶含笑 and 亮葉含笑, both meaning “bright-leaved michelia”.

##### Proposed Chinese name.

亮叶木兰 meaning “bright-leaved magnolia”.

##### Type.

Vietnam – **Annam** (= central Vietnam) • Tourane Province (now Đà Nẵng Municipality), near Tourane (now Đà Nẵng), Ba-na, alt. 1400 m, in forest, tree 12 to 15 m, 12 July 1923, *Eugène Poilane 7092*. holotype: P 00205338! isotypes: BM 000574774! K 000681511! P 00205339! P00205340!

**Paratypes**. Vietnam – **Tourane Province** (now Đà Nẵng Municipality) • near Tourane (now Đà Nẵng), Mount Ba-na, near summit, trail below hotel and Japanese garden, 17 Aug. 1927, *J. & M.S. Clemens 4281*. P n.v. UC n.v. China. **Hainan Province** • Lingao County, Hung-mo-tung district, Hung-mo Shan, near Fan-ta village, wooded mountainside, tree 7 m, 5^th^ Hainan Expedition, 21 Aug. 1929, fr, *F.A. McClure 712*. B 100294232! BM n.v. IBSC 0006064 n.v. NAS 00070753! NY n.v. PE 00028342! PE 00028343! SYS sys00051022 n.v. **ibid**. • Hung-mo Shan, south-west of Fan-ta village, alt. 1500 m, on forested mountainside, tree 25 m, 5^th^ Hainan Expedition, 22–25 Aug. 1929, fr, *Floyd A. McClure & Hom Fung 768*. BM n.v. IBSC 0006056 n.v. NAS 00070754! PE 00934133! SYS sys00095544!

Vietnamese specimen images below accessed 20 Mar. 2019:

holotype (P): http://coldb.mnhn.fr/catalognumber/mnhn/p/p00205338

isotype (P: sheet 1): http://coldb.mnhn.fr/catalognumber/mnhn/p/p00205339

isotype (P: sheet 2): http://coldb.mnhn.fr/catalognumber/mnhn/p/p00205340

isotype (BM): http://data.nhm.ac.uk/object/a7e9dff9-9585-468b-bb6f-9b0e40ad84b4

isotype (K): http://apps.kew.org/herbcat/getImage.do?imageBarcode=K000681511

Chinese (Hainan) specimen image below accessed 20 Mar. 2019:

paratype (B): http://ww2.bgbm.org/Herbarium/specimen.cfm?Barcode=B100294232

Chinese (Hainan) specimen images below accessed 20 Mar. 2019; re-accessed with new URLs 22 Oct. 2022:

paratype (NAS): https://www.cvh.ac.cn/spms/detail.php?id=da7e2eb4

paratype (NAS): https://www.cvh.ac.cn/spms/detail.php?id=da7e2f50

paratype (PE, ex-LU, sheet 1): https://www.cvh.ac.cn/spms/detail.php?id=0751aef6

paratype (PE, ex-LU, sheet 2): https://www.cvh.ac.cn/spms/detail.php?id=08739e02

paratype (PE): https://www.cvh.ac.cn/spms/detail.php?id=f00fd2b8

paratype (SYS): https://www.cvh.ac.cn/spms/detail.php?id=e8dfa289

##### Note 1.

While Dandy viewed *Michelia fulgens* as distinct from *M. foveolata*, it is listed as a synonym of that species in Chen and Nooteboom (1993) and then by the above-noted authors, including Sima (2011), who had previously listed it as a synonym under *Magnolia foveolata* (Merr. ex Dandy) Figlar (Sima 2001). However, *Michelia fulgens* retains its species status in Law (1996: 183), Liu et al. (2004: 262), Xing et al. (2009: 206) and Yang et al. (2016: 276). The present authors consider that *Michelia fulgens* can be easily distinguished from *M. foveolata* by the differentiating morphological features compiled in Table 11.

**Table 11.** The **25 presently known** differentiating features between *Michelia fulgens* Dandy and *Michelia foveolata* Merr. ex Dandy.

**Plant feature**	***Michelia fulgens***	***Michelia foveolata***.
**life form**	**medium-sized tree to 25 m high^2^**	**large-sized tree to 30 m high^5,6^ (to 45 m^3^)**
**trunk diameter**	**to 50 cm**	**to 80 cm**
bark colour	silver-grey (see Fig. 2 at page 80)	pale grey or dark grey^5^
indumentum of buds	densely silver-grey or rufous appressed tomentellous**^2^**	densely rufous tomentellous**^5^**
indumentum of young twigs	densely silver-grey or rufous appressed tomentellous**^2^**	densely rufous tomentellous**^5^**
leaf texture	leathery**^2^**	thickly leathery**^5^**
leaf shape	lanceolate, narrowly ovate or narrowly elliptic-ovate**^2^**	oblong-elliptic, elliptic ovate or broadly lanceolate**^5^**
**leaf dimensions**	**10–20 × 3.5–6.5 cm^2^**	**17–23 × 6–11 cm^4,6^**
leaf apex	acuminate or acute	acuminate or short-acuminate**^5^**
leaf base	cuneate or obtuse, subsymmetrical, occasionally slightly oblique	broadly cuneate, obtuse or subcordate, frequently somewhat oblique**^5^**
leaf indumentum adaxially	initially rufous tomentose, later glabrescent**^2^**	initially coppery pubescent, chiefly near midrib, later glabrescent
leaf indumentum abaxially	silver-grey or rufous appressed tomentellous^2^	coppery-red tomentellous**^5^**
**lateral leaf vein pairs**	**14–20^1^**	**16–26^4^**
**petiole length**	**to 3.5 cm (2–4 cm^1^)**	**to 2.5 cm (1.5–3 cm^4^)**
petiole indumentum	densely silver-grey or rufous appressed tomentellous**^2^**	densely rufous tomentose**^5^**
flower bud bracts	3–5**^1^**	3–4**^4^**
outer tepals shape	elliptic or obovate-elliptic	broadly obovate
**outer tepal size**	**ca. 3 × 1.5 cm^2^**	**6–7 cm long^5^**
**stamen length**	**1.2–1.4 cm^1^**	**2.5–3 cm^4^**
**anther length**	**1.1–1.2 cm^1^**	**1.5–2 cm^4^**
**filament length**	**1–2 mm^1^**	**7–10 mm^4^**
**gynophore length**	**13–15 mm^1^**	**17–20 mm^4^**
ovule number	10 or more**^1^**	12 or more (ca. 8**^4^**)
**fruit aggregate length**	**7–10 cm^2^**	**ca. 8–14 cm (7–20 cm^4,5^)**
mature carpel length	to ca. 2 cm	to ca. 2.5 cm**^4^**

Note: The listed features of *Michelia fulgens* are from Dandy (1930: 210), supplemented by Law (1996: 183)^1^ and Liu et al. (2004: 262)^2^; with those of Michelia foveolata from Dandy (1928b: 360), supplemented by Chen and Nooteboom (1993: 1066)^3^, Law (1996: 181)^4^, Liu et al. (2004: 256)^5^ and Xing et al. (2009: 205)^6^.

##### Note 2.

*Michelia fulgens* with its 9–12 tepalled flowers also does not key out with the original validating descriptions for the 12-tepalled *M. longistyla* Law & Y.F. Wu, for the 17-tepalled *M. oblongifolia* Hung T. Chang & B.L. Chen, or for *M. aenea* Dandy with 6 tepals, all with whom it shares synonymy under *M. foveolata* Merr. ex Dandy in Flora of China.

##### Note 3.

Research published five years ago in which a UPGMA cluster analysis supported the division of Magnoliaceae into the monogeneric subfamilies Magnolioideae and Liriodendroideae, includes a UPGMA clustering diagram of genomic relationships between 135 species of Magnoliaceae (Li 2021: 30, fig. 3.6). This reveals the closest relative to *Michelia fulgens* to be M. foveolata var. xiangnanensis (authors: C.L. Peng & L.H. Yan), with *M. foveolata* a distant relative on the opposite side of the genome map. A previously published study of the genetic diversity of 48 wild species of Magnoliaceae in Yunnan Province, utilising ISSR molecular markers, showed *Michelia fulgens* closest relationship with *Michelia aenea* (铜色含笑) on the accompanying UPGMA cluster map. However, *M. aenea* is erroneously translated from the Chinese as *Michelia foveolata* which, in fact, appears on the opposite side of the cluster map to *M. fulgens* and *M. aenea* (Wang et al. 2020: 281, fig. 2).

##### Note 4.

In addition to the above, an earlier study utilising AFLP and ISSR markers to determine the genetic relationship of some Magnoliaceae evergreen plants occurring in China found that there was a large genetic diversity among the 21 taxa analysed, including between *M. fulgens* and *M. foveolata*, and that they were completely distinguishable as independent species (Li 2013). Since there is a general consensus on the independence of M*ichelia fulgens*, this species is reinstated and transferred to *Magnolia*.

##### Note 5.

*Michelia fulgens* is recorded in *ex situ* conservation at Zhongshan Arboretum in Guangdong Province from test seedlings planted in March 2006 that were received from Fairy Lake Botanical Garden in Shenzhen, also in Guangdong Province. (Xiu et al. 2013). The occurrence of *Michelia fulgens* in China at about another 20 botanical gardens, arboreta plus college and university campuses (five of which also cultivate *M. foveolata*!) and at 22 nature reserves, has been noted from a search of the literature by the present authors. Also, three reports mention the wild occurrence of *Michelia fulgens* at specified locations of 500 m, 700–1000 m and 760–1040 m altitude, which, if it’s correctly identified, are well below that of 1300–1700 m recorded for this species in Magnolias of China. In central Vietnam, Bach Ma National Park near Hue and the 38210 ha Ba Na-Nui Chua Nature Reserve near Da Nang (Anon 2004), now encompass locations where *M. fulgens* was collected in the first half of last century, so it possibly still survives in this region in unsubstantiated synonymy as *Magnolia foveolata*.

##### Conservation status.

*Magnolia foveolata*, including *Michelia fulgens* and other taxa as synonyms, was assessed for the IUCN Red List of Threatened Species by S. Khela on 25 September 2012 as of Least Concern “as it has a wide distribution in China” but that “more up to date information is needed and its distribution and status in Vietnam is unknown” (IUCN 2026).

##### Recommendation.

While *Michelia fulgens* can be previously seen to be adequately protected in *ex situ* conservation in China and equally so *in situ* within nature reserves in China, its IUCN conservation status as *Magnolia fulgens* independent of *M. foveolata* and the other synonymised species at the IUCN site should be assessed as soon as practical, including any wild populations remaining in Vietnam, particularly in the Ba-na region from where it was first collected and which preceded collections later made in China.

#### Magnolia glaberrima

Taxon classificationPlantaeMagnolialesMagnoliaceae

(Hung T. Chang) C.B. Callaghan & S.K. Png comb. nov.

0DC8E614-8D59-5F63-8A15-17CC6551C145

urn:lsid:ipni.org:names:77383104-1

Table 12

Michelia glaberrima Hung T. Chang, Acta Scientiarum Naturalium Universitatis Sunyatseni 1961(1): 56 (Chang 1961). **[Basionym]**.
Michelia chapensis Dandy, in Annals of Missouri Botanical Garden: 1079 (Chen and Nooteboom 1993), World Checklist and Bibliography of Magnoliaceae: 55 (Frodin and Govaerts 1996), Flora Yunnanica. Tomus 6 (Spermatophyta): 48 (Wu and Chen 2006), Flora of China Vol. 7: 88 (Xia et al. 2008), Vouchered Flora of Southeast Yunnan, Vol. 1: 51 (Sima and Lu 2009), A Taxonomic Revision of the Magnoliaceae from China: 248 (Sima 2011) and Magnoliaceae Plants of Guizhou: 180 (Deng and Yang 2015), each p.p. quoad syn. Michelia glaberrima Hung T. Chang.
Magnolia chapensis (Dandy) Sima, in Yunnan Forestry Science and Technology 2001(2): 29 (Sima 2001), and online at World Checklist of Magnoliaceae (Govaerts 2003), both p.p. quoad syn. Michelia glaberrima Hung T. Chang.

##### Present Chinese name.

秃含笑 meaning “bald michelia”.

##### Proposed Chinese name.

中国秃木兰 meaning “Chinese bald magnolia”.

##### Type.

China. **Kwangtung (= Guangdong) Province** • Hwei-ji (= Huaiji) County, Waitsap District, Dang Shan, near Shiliu-bao village, 10 Sept. 1933, *Tsang Wai-tak 22753*. holotype (incorrectly labelled for Guangxi): SYS sys00050932 n.v. **ibid**. • Tong Shan, along Kwangtung border, Waitsap District, near Sap-luk Po village, fairly common, silt, sandy soils, thickets, 2.74 m shrub, 10 Sept. 1933, fr, *Tsang Wai-tak 22753*. isotypes: A 00936592! IBSC 0053160 n.v. LE 01013056!

Specimen images below accessed 20 Mar. 2019:

isotype (A ex-LU): https://kiki.huh.harvard.edu/databases/specimen_search.php?mode=details&id=1299275

isotype (LE): https://en.herbariumle.ru/?t=occ&id=23642&rid=image_0052677

##### Note 1.

*Michelia glaberrima* was noted as “essentially the same” as *M. chapensis* Dandy in Chen and Nooteboom (1993) and consequently reduced as a synonym of that species, as it has since been treated by the authors recorded in the above synonymy. However, *M. glaberrima* may be differentiated from *M. chapensis* by the morphological features listed in Table 12. Also, the distribution of the two species is markedly different, *Michelia glaberrima* occurring only at the two known localities in Huaiji County, Guangdong, while *M. chapensis* is recorded as more widely distributed in S Jiangxi, N & E Guangxi, W Hunan, N Guangdong and also in Vietnam, although this is inflated somewhat by the inclusion of the numerous synonyms mentioned in Note 2. *Michelia glaberrima* is evidently an independent species, and is discounted as a synonym of both *Michelia skinneriana* and *M. figo* in Chang’s diagnosis distinguishing it from these species due to being “easily recognised by its absolutely glabrous branches and leaves, and longer petioles”.

**Table 12.** The **17 presently known** differentiating features between *Michelia glaberrima* Hung T. Chang and *Michelia chapensis* Dandy.

**Plant feature**	***Michelia glaberrima***	***Michelia chapensis***
**life form**	**2–3 m shrub, with many congested terminal twigs**	**large-sized tree to 30 m height^1,2^**
leaf shape	oblong.	obovate, narrowly obovate or oblong-obovate**^2^**
**leaf dimensions**	**7–10 × 3–4 c**m	**6.5–15 × 3.5–6.5 cm^2^**
leaf apex	sub-acute or obtuse-acute	abruptly narrow short-acuminate or short acuminate with obtuse mucro
leaf texture	leathery.	thinly leathery**^2^**
**lateral leaf vein pairs**	**8–10**	**10–15**
**reticulate leaf veins**	**prominent both surfaces**	**prominent only below**
**petiole length**	**0.8–1.5 cm**	**1.5–2.5 cm^1^**
gynophore length	10 mm	ca. 7 mm**^1^**
gynophore indumentum	puberulus.	densely appressed puberulent**^2^**
**fruit peduncle length**	**3–5 mm**	**ca. 20 mm^1^**
**fruit aggregate length**	**3–4 cm**	**ca. 10 cm**
mature carpels shape	compressed ellipsoid	ellipsoid or ovoid**^2^**
**mature carpels size**	**1.6 × 1 cm**	**1–1.5 × 1 cm^1^**
seed shape	sub-ovoid	ovoid or oblong ovoid**^1^**
seed dimensions	6 mm long	10 × 6 mm**^1^**
region of distribution in Guangdong Province	western Guangdong near Guangxi border	northern Guangdong^2^

Note: The listed features of *Michelia glaberrima* are from Chang (1961: 56), who records the flowers as unseen. Those of *Michelia chapensis* are from Dandy (1929b: 223), supplemented by Law (1996: 170)^1^, Liu et al. (2004: 232)^2^ and Yang et al. (2016: 244)^3^.

##### Note 2.

The 3 metre tall *Michelia glaberrima* also does not key out with the original descriptions for the 26 m tall *M. brachyandra* B.L. Chen & S.C. Yang from Yunnan with leaves to 12 × 5.3 cm, the 23 m tall *M. chartacea* from Yunnan with 10.5–28 cm long aggregate fruits, the Vietnamese occurring *M. constricta* Dandy with papery-textured leaves to 11 × 5.5 cm, the 25 m tall *M. microcarpa* B.L. Chen & S.C. Yang from Yunnan with leaves to 13.7 × 4.2 cm, or the 10 m tall *M. tsoi* Dandy from Guangdong with leaves to 12 × 5.5 cm, all with whom it shares synonymy under *M. chapensis* Dandy in Flora of China. In view of the substantiation of *Michelia glaberrima* as a genuine species, it is resurrected and transferred to *Magnolia*.

##### Note 3.

As far as can be ascertained, there were no further collections of *Michelia glaberrima* made in the 60 years from its initial collection in 1933 through to its being named in 1961 and subsequently subsumed as a synonym of *M. chapensis* in 1993, or any collections made since then. This, despite the fact that it occurs (or occurred?) only a few hours away from the world’s premier site of Magnoliaceae conservation at South China Botanical Garden founded in 1929. Perhaps *M. glaberrima* could not be relocated because while the type specimens were collected in Guangdong close to the Guangxi border, their labels are captioned the 3^rd^ Kwangsi Expedition, possibly leading to the assumption they were collected in Guangxi.

##### Conservation status and recommendation.

The conservation status of *Michelia chapensis* was noted as Rare in the 1997 IUCN Red List of Threatened Plants (Walter and Gillett 1998: 388), and was last assessed in 2014 as Least Concern (IUCN 2026). Being a synonym of that species for the past 33 years has resulted in there being no known independent assessments by Chinese authorities or the IUCN of how *Michelia glaberrima* (now *Magnolia glaberrima*) has fared in the wild during this period of time, or whether it even still exists in its two recorded localities in China’s most populous province, a situation which needs to be rectified at the earliest opportunity! The residents of the two villages mentioned for the type specimens that were collected on Tong Shan and Dang Shan mountains may hold the clue to its fate.

#### Magnolia hainanensis

Taxon classificationPlantaeMagnolialesMagnoliaceae

(Dandy) C.B. Callaghan & S.K. Png comb. nov.

2D7E73CD-C409-5F9B-B53C-926903F4A995

urn:lsid:ipni.org:names:77383114-1

Table 13, Figs 6, 7

Manglietia hainanensis Dandy, The Journal of Botany: British and Foreign 68(7): 204 (Dandy 1930). **[Basionym]**.
Manglietia fordiana Oliv., in Annals of Missouri Botanical Garden: 1039 (Chen and Nooteboom 1993: 1039), Flora Yunnanica. Tomus 6 (Spermatophyta): 12 (Wu and Chen 2006) and Vouchered Flora of Southeast Yunnan, *Vol. 1*: 23 (Sima and Lu 2009), each p.p. quoad syn. Manglietia hainanensis Dandy—A Taxonomic Revision of the Magnoliaceae from China: 88 (Sima 2011), p.p. quoad syns. Manglietia hainanensis Dandy, M. fordiana var. hainanensis (Dandy) N.H. Xia, Magnolia fordiana var. hainanensis (Dandy) Noot.—Magnoliaceae Plants of Guizhou: 32 (Deng and Yang 2015), p.p. quoad syns. Manglietia hainanensis Dandy, Manglietia fordiana var. hainanensis (Dandy) N.H. Xia and Magnolia fordiana var. hainanensis (Dandy) Noot.
Manglietia fordiana var. fordiana , in Annals of Missouri Botanical Garden 80(4): 1039 (Chen and Nooteboom 1993), p.p. quoad specimen *F.A. McClure 9652*, and online at World Checklist of Magnoliaceae (Govaerts 2003), both p.p. quoad syn. Manglietia hainanensis Dandy.
Manglietia fordiana var. hainanensis (Dandy) N.H. Xia, in Flora of China Vol. 7: 58 (Xia et al. 2008), p.p. excl. syn. Manglietia albistaminea Law et al.
Magnolia fordiana var. hainanensis (Dandy) Noot., in Flora of China Vol. 7: 49 (Xia et al. 2008).

##### Present Chinese name.

海南木莲 meaning “Hainan manglietia”.

**Figures 6, 7.**
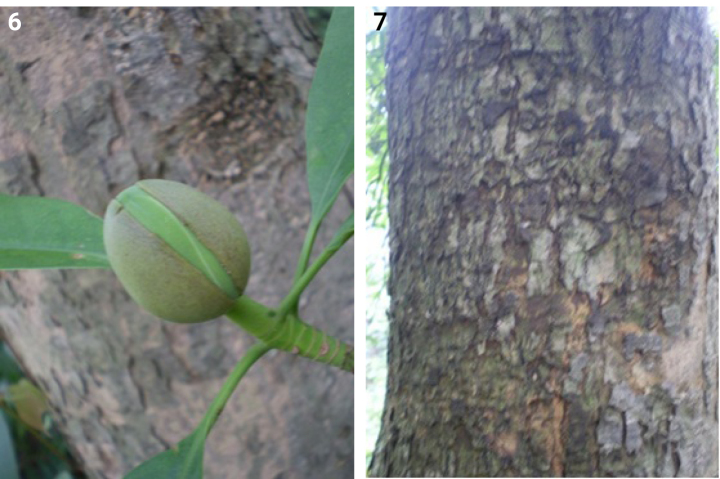
*Magnolia hainanensis* (synonym *Manglietia hainanensis*). Expanding flower bud showing green lower surface of outer tepals within expanding bracts (left) and bark close-up (right). Photos taken by the authors at South China Botanical Garden, Guangzhou, 21 April 2017. © Callaghan & Png (ABA).

##### Proposed Chinese name.

海南木兰 meaning “Hainan magnolia”.

##### Vietnamese names.

Mỡ hải nam meaning “Hainan manglietia” and Mỡ Ba Vì meaning “Ba Vi manglietia” after its apparent occurrence in the Ba Vi Mountains west of Hanoi.

##### Type.

China. **Hainan Province** • Hung-mo-tung District, Hung-mo Shan, 19°12'1.82"N, 109°44'29.72"E, east of Fan-ta village, in mid. of mountain and ravines; tree 10 m, 8 Aug. 1929, fr, *Tsang Wai-tak & Fung Hom 656*. holotype: BM 000946042! isotypes: A 00039035! BM 000571245! BM 000574407! IBSC 0006074 n.v. K 000681421 MO n.v. NY 00320706! SYS sys00095529!

**Paratypes. ibid**. • B 100294233! NAS 00070746! US 00432539! • Fan-ya to Yiktsok-mau, tree 8 m, 17 May 1922, *Floyd Alonzo McClure 9652*. SYS sys00095530!

Specimen images below accessed 20 Mar. 2019; reaccessed 22 Oct. 2022 with new URLs for SYS, NAS:

holotype (BM): http://data.nhm.ac.uk/object/3dfa59ba-509a-4db7-97b6-e7d573c0d885

isotype (A): http://kiki.huh.harvard.edu/databases/image.php?id=304811

isotype (BM): http://data.nhm.ac.uk/object/d7f0ac10-0268-44ee-a39a-ff809e2d9190

isotype (BM): http://data.nhm.ac.uk/object/2df90242-4fa2-41b3-8a8d-a03a5e004d93

isotype (K): http://apps.kew.org/herbcat/getImage.do?imageBarcode=K000681421

isotype (NY): http://sweetgum.nybg.org/science/vh/specimen_details.php?irn=415964

isotype (SYS ex-LU): https://www.cvh.ac.cn/spms/detail.php?id=e8b7e794

paratype (B): http://ww2.bgbm.org/Herbarium/specimen.cfm?Barcode=B100294233

paratype (NAS): https://www.cvh.ac.cn/spms/detail.php?id=da7e2a64

paratype (US ex-LU): https://collections.nmnh.si.edu/media/?i=10093838

paratype (SYS ex-CCC): https://www.cvh.ac.cn/spms/detail.php?id=e8b1dc5a

##### Note 1.

An identification label dated 18 May 1990 is affixed to the isotype specimen at Kew confirming its identity as *Manglietia hainanensis* Dandy and another dated 15 April 1991 has its determination as *M. fordiana* Oliv., with both being signed by Chen Bao-liang. *M. hainanensis* is subsequently noted as conspecific with *M. fordiana* in Chen and Nooteboom (1993), because “it differs from the latter only in the length of the pedicle” (not pedicel, Chen and Nooteboom 1993: 999) “and the texture of the leaves” (Dandy mentioned two other differences). *Manglietia hainanensis* is also placed in synonymy under *M. fordiana* by Sima and Lu (2009) and in Sima (2011), having regained its species status in the intervening Flora Reipublicae Popularis Sinicae in 1996 and Magnolias of China in 2004 but then made a variety of *M. fordiana* in Flora of China in 2008. The present authors have found *Manglietia hainanensis* to be differentiated from *M. fordiana* by their features compiled in Table 13.

**Table 13.** The **26 presently known** differentiating features between *Manglietia hainanensis* Dandy and *Manglietia fordiana* Oliv.

**Plant feature**	***Manglietia hainanensis***	***Manglietia fordiana***
**life form**	**large-sized tree to 30 m high^2,5^**	**medium-large sized tree to 20 m^8^ (to 22 m^9, 12^, to 25 m^11^)**
**trunk diameter**	**to 100 cm^2^**	**to 45 cm^11^**
bud indumentum	appressed pilose**^2^**	pilose**^8^**
young twigs indumentum	appressed pilose**^2^**	pilose**^8^**
**leaf dimensions**	**10–20 × 3–7 cm^2,5^**	**8–17 × 2.5–5.5 cm^8,13^**
leaf texture	thinly leathery**^2^**	leathery**^8^**
leaf shape	obovate, narrowly obovate or narrowly elliptic-obovate**^2^**	narrowly obovate, narrowly elliptic-obovate or oblanceolate**^8^**
leaf apex	acute or acuminate**^2^**	short-acute with obtuse mucro**^8^**
leaf indumentum abaxially	sparsely appressed pilose**^2^**	sparsely pilose**^8^**
**lateral leaf vein pairs**	**12–16^1^**	**8–12^7^**
**petiole length**	**3–4 (–4.5) cm^1^**	**1–3 cm^7^**
**flower peduncle length**	**8–40 mm^1^**	**6–11 mm^7^**
outer 3 tepals shape	obovate.	oblong-elliptic
**outer 3 tepals dimensions**	**5–6 × 2.8–3.2 cm**	**6–7 × 3–4 cm**
**outer 3 tepals colour**	**white above, green below^2^** (see Fig. 6)	**both surfaces pure white^8^**
**stamen-bearing receptacle length**	**0.6–1.5 cm^4^**	**1.9–2.54 cm (Plate 1953, Oliver 1891)**
pollen shape	oval**^3^**	oblong**^10^**
**gynoecium colour**	**green^1^**	**yellow^1^**
**gynoecium length**	**1.5–2 cm^1^**	**ca. 1.5 cm^7^**
immature carpels	18–32**^1^**, no beak	23–30**^7^**, beaked at apex
ovules each carpel	10 or more (8–16**^4^**)	8–10**^7^** (8–12**^11^**)
fruit aggregate shape	ovoid or ellipsoid-ovoid	ovoid.
**fruit aggregate length**	**5–6 cm^2^**	**to 3.5 cm^6^ (2–5 cm^8^)**
flowering period	April–May	May
fruiting period	September–October**^2^**	October**^8^**
distribution.	China: Hainan northern Vietnam	Hong Kong, Guangdong, Fujian, Jiangxi, Hunan, Guangxi, Guizhou, Yunnan**^8^**

Note: The listed features of *Manglietia hainanensis* are from Dandy (1930: 204), supplemented by Law (1996: 105)**^1^**, Liu et al. (2004: 150)**^2^**, Sang et al. (2016)**^3^**, Tiêṕ (1980: 539; 567)**^4^** and Xing et al. (2009: 195)**^5^**. Those of *Manglietia fordiana* are from Oliver (1891), plus Dandy (1928c: 127)**^6^**, Law (1996: 105)**^7^**, Liu et al. (2004: 138)**^8^**, Peng (2008: 84)**^9^**, Sang et al. (2016)**^10^**, Sutton (2022)^11^, Tiêṕ (1980: 538; 567)**^12^**, Xing et al. (2009: 193)^13^ and Yang et al. (2016: 161)**^14^**.

##### Note 2.

In a study utilising SEM (scanning electron microscopy), the pollen morphology of *Manglietia hainanensis* was observed as oval, nearly circular at both ends, nearly circular in polar view, while the pollen of *M. fordiana* was noted as oblong, pointed at both ends and short oval in polar view (Sang et al. 2016). In a phylogenetic tree of 27 Magnoliaceae species which was constructed based on matK and trnH sequences (Liu et al. 2015: 4, fig. 1), *M. hainanensis* (as Magnolia fordiana var. hainanensis) is shown to be distantly related to *Manglietia fordiana* (as *Magnolia fordiana*). Previously, in a research paper published in 2011, *Manglietia hainanensis* was shown to be a distinct, independent species from *M. fordiana* on the basis of DNA evidence (Xiao et al. 2011a: 492, fig. 2). This, plus the features compiled in Table 13, leads the present authors to follow Law (1996: 105), Liu et al. (2004: 150), Xing et al. (2009: 195) and Yang et al. (2016: 177) in recognising *Manglietia hainanensis* as a genuine species which is reinstated and transferred to *Magnolia*.

##### Note 3.

According to the literature searched by the present authors in Australia and overseas for a future article, *Manglietia hainanensis* is recorded for six nature reserves in China and for two nature reserves and one national park in Vietnam. *M. hainanensis* is noted as occurring in *ex situ* conservation in China at three forest research institutions (at one as M. fordiana var. hainanensis), plus 11 botanical gardens or arboreta (at two as M. fordiana var. hainanensis), including South China Botanical Garden, Guangzhou, Guangdong, and Nanyue Arboretum, on the south peak of Heng Shan, Hunan, both in which *M. hainanensis* and *M. fordiana* occur.

##### Conservation status.

*Manglietia hainanensis* does not appear at the current IUCN Red List of Threatened Species [IUCN 2026], but was previously noted there in 2019 as “not yet assessed”. *Manglietia hainanensis* was assessed by Chinese authorities using IUCN criteria as VU A2c (Wang and Xie 2004: 327). It was also recorded as a fully endangered endemic species on Hainan Island (Lian et al. 2013: 34) and in China it is listed as a Level 3 National Protected Plant due to the small number of existing populations, poor reproductive capacity of its seeds and human activities (Wei et al. 2010: 91), with these authors also subsequently utilizing ISSR technique to study the genetic diversity among four *ex situ* populations of *M. hainanensis* cultivated in the subtropical localities of Xinning and Changsha, Hunan that were introduced from the tropical locality of Ledong, Hainan. (Wei et al. 2013). While apparently referencing the previously noted 2004 vulnerable assessment, another Chinese author records the alarming accelerating decline of the natural forest coverage on Hainan Island from 25.5% in 1956 to a mere 4% percent in 1999 (Yan 2006: i), with serious damage to the island’s forest resources being warned of as far back as 1982 (Tang 1982: 122). Despite large investments in reafforestation since at least 1999, it has been reported that the natural forests in Hainan are being mostly replaced by non-native monocultural plantations, including encroachment into protected areas in this key tropical biodiversity hotspot (Zhai et al. 2014: 611). From a Vietnamese perspective, *Manglietia hainanensis* was assessed for that country in 2005 as Endangered, EN D, again utilising IUCN criteria (Chien et al. 2006: 41).

##### Recommendation.

Because of the bleak picture painted above, the present IUCN *in situ* conservation status of *Magnolia hainanensis* should be determined without undue delay!

#### Magnolia hedyosperma

Taxon classificationPlantaeMagnolialesMagnoliaceae

(Law) C.B. Callaghan & S.K. Png comb. nov.

09C1D480-A499-5B4C-8B48-FB270775876D

urn:lsid:ipni.org:names:77383135-1

Table 14

Michelia hedyosperma Law, Bulletin of Botanical Research 5(3): 123–124, 129: fig. 3 (Law 1985). **[Basionym]**.
Michelia hypolampra Dandy, in Annals of Missouri Botanical Garden 80(4): 1076 (Chen and Nooteboom 1993), p.p. quoad syn. Michelia hedyosperma Law plus specimen *Y.H. Li 4262*—World Checklist and Bibliography of Magnoliaceae: 57 (Frodin and Govaerts 1996), p.p. quoad syn. Michelia hedyosperma Law.
Magnolia hypolampra (Dandy) Figlar, online at World Checklist of Magnoliaceae (Govaerts 2003), p.p. quoad syn. Michelia hedyosperma Law.
Michelia gioi (A. Chev.) Sima & Hong Yu, in Flora Yunnanica. Tomus 6 (Spermatophyta): 50 (Wu and Chen 2006), Flora of China Vol. 7: 89 (Xia et al. 2008), Vouchered Flora of Southeast Yunnan, Vol. 1: 58 (Sima and Lu 2009) and A Taxonomic Revision of the Magnoliaceae from China: 208 (Sima 2011), each p.p. quoad syn. Michelia hedyosperma Law.

##### Present Chinese names.

香子含笑 or 香籽含笑 both meaning “fragrant seed michelia”.

##### Proposed Chinese name.

香子木兰 meaning “fragrant seed magnolia”.

##### Type.

China. **Guangxi Zhuang Autonomous Region** • Longzhou County, Daqing Shan, date (?), *Chia Liang-chih & Feng Xue-lin 6054*. holotype: IBSC 0053839 n.v.

**Paratypes**. China. **Hainan Province** • East Bawang, northern slope, Highway No.2, Jan. 1976, *Forestry Bureau 463*. IBSC 0053834 n.v. – **Yunnan Province** • Xishuang-banna Prefecture, 21°59'N, 101°12'E, alt. 960 m, dense forest in damp valley, 32 m tree, 29 May 1962, *Li Yan-hui 4262*. IBK 00000591! HITBC 001666! HITBC 001667! HITBC 001668! KUN 41820 n.v.

holotype (IBSC) and paratypes (IBSC, KUN): specimen images not uploaded to CVH at 18 Mar. 2026.

Specimen images below accessed 20 Mar. 2019; re-accessed with new URLs 22 Oct. 2022:

paratype (IBK): https://www.cvh.ac.cn/spms/detail.php?id=c007dcbd

paratype (HITBC): https://www.cvh.ac.cn/spms/detail.php?id=e9543695

paratype (HITBC): https://www.cvh.ac.cn/spms/detail.php?id=e95442de

paratype (HITBC): https://www.cvh.ac.cn/spms/detail.php?id=e95441a1

##### Additional specimen.

China. **Guangxi Zhuang Autonomous Region** • Longzhou County, alt. 600 m, tree 25 m, 26 Sept. 1973, fr, *Vegetation Survey Team 055*. PE 00934086! Specimen image below accessed 2 Dec. 2022:

(PE): https://www.cvh.ac.cn/spms/detail.php?id=f00fec56

##### Note 1.

*Michelia hedyosperma* Law is noted as conspecific with *M. hypolampra* Dandy in Chen and Nooteboom (1993) on the basis of Law’s description and figure and then listed as a synonym of *M. gioi* (A. Chev.) Sima & Hong Yu. The IAPT Nomenclature Committee for Vascular Plants, on the information provided by R. Govaerts, voted 15 to 2, with one abstention, to recommend that *Talauma gioi* A. Chev. not be considered a validly published name because “the descriptive material does not constitute a description or diagnosis” (Govaerts and Nooteboom 2010). This recommendation was approved in its 11^th^ report by the General Committee in 2011 and ratified at the International Botanical Congress in Melbourne, Australia, that same year.

(The above was noted as a Flora of China post-publication correction at: http://www.eflora.cn/foc/pdf/FOCV1/FOC-01-06-Corrigenda.pdf which is now not accessible).

As a consequence of its invalidation, this taxon described from Vietnam and its later combination *Michelia gioi* (A. Chev.) Sima & Hong Yu is not considered here in relation to the other two taxa. *Michelia hedyosperma* may be seen to be an independent species from *M. hypolampra* by their presently known differentiating features recorded in Table 14.

**Table 14.** The **15 presently known** differentiating features between *Michelia hedyosperma* Law and *Michelia hypolampra* Dandy.

Plant feature	***Michelia hedyosperma***	***Michelia hypolampra***
**life form**	**tree to 32 m (Yunnan paratype labels)**	**tree to 21 m^3^**
leaf shape	obovate or elliptic-obovate	obovate-oblong or elliptic
maximum leaf size	13 × 5.5 cm	12 × 6 cm
leaf apex	cuspidate.	acuminate.
leaf indumentum	glabrous.	glabrous, or initially minutely grey pubescent at midrib margins near base
**lateral leaf vein pairs**	**8–10**	**10–12**
petiole indumentum	densely appressed short sericeous, later glabrous**^1^**	initially minutely pubescent, later glabrescent
**flower peduncle length**	**ca. 10 mm**	**ca. 5 mm**
flower peduncle indumentum	densely appressed short sericeous**^1^**	short appressed pubescent
**gynoecium indumentum**	**glabrous^1^**	**appressed minutely shiny grey tomentose**
immature carpels	ca. 10	ca. 8–10
**ovules each carpel**	**6–8**	**ca. 10 or more**
**seeds in mature carpels**	**1–4**	**ca. 1–2^4^**
flowering period	March–April	Oct.–Dec.**^2^** (?) [see note 3 herein]
distribution.	China: Hainan, SW Guangxi and southern Yunnan**^1^**	Vietnam: Nghệ An Province (possibly also Tuyên Quang Province?)

Note: The listed features of *Michelia hedyosperma* are cited from Law (1985: 123), supplemented by Liu et al. (2004: 274)**^1^**; those of *Michelia hypolampra* are from Dandy (1928a: 321), to whom only immature fruit was available, Sima and Lu (2009)^2^, Xia et al. 2008)^3^. Photos at Vietnam Plant Database show max. 2 mature seeds per carpel**^4^**: https://www.botanyvn.com/cnt.asp?param=edir&v=~~~PROTECTED_TN_664~~~%20hypolampra&list=species&lg=en [acc: 30 Oct. 2022]

##### Note 2.

As shown in the illustration in Magnolias of China, the mature long-stalked, 2–4.5 cm ellipsoid carpels of the fruit aggregates of *Michelia hedyosperma* are subtended by a long scale-leaf appendage extending beyond the carpels that is not, to our knowledge, known for other *Michelia* species, including *M. hypolampra*. *Michelia hedyosperma* is native to China in the provinces of Hainan, SW Guangxi and southern Yunnan, while *M. hypolampra* apparently occurs only in Vietnam’s northern-central Nghệ An Province, where the type was collected, and possibly in the more northern Tuyên Quang Province. Therefore, the present authors follow the treatment in Fu and Jin (1992: 442), Law (1996: 173), Liu et al. (2004: 274) and Yang et al. (2016: 282) in recognising *Michelia hedyosperma* as a distinct species which is reinstated and transferred to *Magnolia*.

##### Note 3.

Inexplicably, the flowering of *Michelia gioi*, under which *M. hedyosperma* and *M. hypolampra* are listed in synonymy in Xia et al. (2008: 89) and Sima and Lu (2009: 58), is recorded in the first publication for Mar.–Apr. and in the second for Oct.–Dec., when the flowering of *M. hedyosperma* (*sensu stricto*) is noted for Oct.–Feb. in Fu and Jin (1992), yet noted for Mar.–Apr. in both Law (1996) and Liu et al. (2004). Also in the same year, Guan and Zhang (2004), record the flowering period for cultivated plants studied at Xishuangbanna Dai Autonomous Prefecture located in southwestern Yunnan Province as November to February of the next year.

##### Note 4.

According to the literature searched by the present authors in Australia and overseas, *Michelia hedyosperma* is recorded in China for 15 nature reserves, as well as being recorded as occurring in *ex situ* conservation at five forest research institutions, 11 botanical gardens or arboreta plus other places, indicating it to be well-established in cultivation and an accepted species by the botanical authorities at these gardens and arboreta.

##### Conservation status.

The *Michelia hedyosperma* of Hainan Island were found to be in dire need of prioritisation for conservation due to their small population size and lack of suitable habitats (Chen et al. 2014). *M. hedyosperma* is also recorded as an endangered species under State (i.e. National) Protection Category II (Liu et al. 2004), having been previously listed as a level 3 provincial key protected plant on the Yunnan Key Protected Wild Plants List published in 1989 (Li and Li 1999: 4), yet is not mentioned in a 2003 paper concerning the 90 species of national priority protected wild plants in Yunnan (Li et al. 2003: 184). The 2003 authors mention that during a comprehensive four-year survey by the Yunnan Forestry Department, researchers could only find 87 of the total 90 species listed in their Table of Priority Protection Plants in Yunnan, so it may be that *Michelia hedyosperma* was already extinct in the wild in at least some of its Yunnan habitats by 2003 (?). A 2012 report of the United Nations Food and Agricultural Organisation listing 307 of the major Chinese rare and endangered plant species that were included in previous Chinese government publications, indicates that *Michelia hedyosperma* was assessed with a medium threat level due to unsustainable logging and habitat fragmentation (Anon 2012: appendix 28, 33). However, *M. hedyosperma* had been previously recorded early this century as endangered in Hainan, southwest Guangxi and southern Yunnan due to overcutting, general habitat destruction and/or competitive effect (Wang and Jiang 2001: 45).

##### Recommendation.

Subsequent to *Michelia hedyosperma* being listed as a level 3 provincial key protected plant on the Yunnan Key Protected Wild Plants List (Li and Li 1999), it has been assessed by Chinese authorities using IUCN criteria as Endangered EN A2c (Wang and Xie 2004: 328). Therefore, it is highly desirable that an evaluation of its present situation in the wild as *Magnolia hedyosperma* (syn. *Michelia hedyosperma*) is undertaken for the IUCN Red List of Threatened Species where, as *Michelia hedyosperma*, it is currently noted as one of a number of synonyms of *Magnolia hypolampra* which was last assessed as far back as April 2007 as “Data Deficient” (IUCN 2026). Coincidentally, that same year *Magnolia hypolampra* was assessed by the China Expert Workshop as Vulnerable VU C1, as is recorded in The Red List of Magnoliaceae (Cicuzza et al. 2007: 26).

#### Magnolia kerrii

Taxon classificationPlantaeMagnolialesMagnoliaceae

(Craib) C.B. Callaghan & S.K. Png comb. nov.

63B06D44-F436-5802-9072-65B1790D51B2

urn:lsid:ipni.org:names:77383206-1

Table 15

Michelia kerrii Craib, Kew Bulletin of Miscellaneous Information 1922: 166 (Craib 1922). **[Basionym]**.
Michelia floribunda Finet & Gagnep., in Annals of Missouri Botanical Garden 80(4): 1064 (Chen and Nooteboom 1993), World Checklist and Bibliography of Magnoliaceae: 57 (Frodin and Govaerts 1996), Acta Botanica Yunnanica 19(2): 135 (Li 1997), Flora Yunnanica. Tomus 6 (Spermatophyta): 43 (Wu and Chen 2006), Flora of China Vol. 7: 81 (Xia et al. 2008), Vouchered Flora of Southeast Yunnan, Vol. 1: 54 (Sima and Lu 2009), A Taxonomic Revision of the Magnoliaceae from China: 198 (Sima 2011) and Magnoliaceae Plants of Guizhou: 133 (Deng and Yang 2015), each p.p. quoad syn. Michelia kerrii Craib.
Magnolia floribunda (Finet & Gagnep.) Figlar, in Yunnan Forestry Science and Technology 2001(2): 31 (Sima 2001), online at World Checklist of Magnoliaceae (Govaerts 2003), and in Thai Forest Bulletin (Botany) 37: 123 (Nooteboom and Chalermglin 2009), each p.p. quoad syn. Michelia kerrii Craib.

##### Type.

Northern Thailand – **Chiang Mai Province** • Doi Suthep (mountain 15 km northwest of Chiang Mai city), alt. ca. 1650 m, tree ca. 10 m, evergreen jungle, 22 Dec. 1920, fl, fr, *Arthur Francis G. Kerr 4679*. holotype: K 000681513! isotypes: BM 000574773! K 000681512! P 00205306! TCD 0009723! (via JSTOR), UC n.v.

##### Topotype.

**Chiang Mai Province** • Doi Suthep, alt. ca. 1676 m, no date, *Rodolphe Meyer d’Schauensee s.n*. PH 00017355**! (**via JSTOR).

Specimen images below accessed 20 Mar. 2019:

holotype (K): http://apps.kew.org/herbcat/getImage.do?imageBarcode=K000681513

isotype (BM): http://data.nhm.ac.uk/object/8647a147-d1a2-4fca-a71d-3ab841a40736

isotype (K): http://apps.kew.org/herbcat/getImage.do?imageBarcode=K000681512

isotype (P): http://coldb.mnhn.fr/catalognumber/mnhn/p/p00205306

isotype (TCD): http://plants.jstor.org/stable/10.5555/al.ap.specimen.tcd0009723

topotype (PH): http://plants.jstor.org/stable/10.5555/al.ap.specimen.ph00017355

##### Note 1.

Both holotype specimen sheets of *Michelia kerrii* Craib at Kew have labels affixed by Dandy in 1926 determining them to be *M. floribunda* Finet & Gagnep. under which *M. kerrii* is recorded as a synonym by the authors previously listed. However, *M. kerrii* can be readily distinguished from *M. floribunda* by the known differentiating features compiled in Table 15.

**Table 15.** The **26 presently known** differentiating features between *Michelia kerrii* Craib and *Michelia floribunda* Finet & Gagnep.

**Plant feature**	***Michelia kerrii***	***Michelia floribunda***
**life form**	**small tree to ca. 10 m high**	**medium-sized tree to 20 m high^5^**
**bark colour**	**dark brown**	**grey^4^**
branchlet indumentum	appressed ferrugineus-pubescent or subsericeous, soon glabrescent	greyish-white appressed hairs when young**^4^** (appressed-puberulous yellowish hairs**^5^**)
leaf shape	lanceolate, oblong-lanceolate or broadly lanceolate	lanceolate, narrowly ovate-elliptic or narrowly obovate elliptic**^4^**
leaf dimensions	to 15 × 4.3 cm	to 12 × 4 cm**^4^**
leaf apex	acuminate or gradually narrowed	acuminate or caudate acuminate**^4^**
leaf base	very often with unequal sides, cuneate, or almost rounded-cuneate	attenuate with equal sides (broadly cuneate or rounded**^4^**)
leaf texture	rigidly papery	leathery.
leaf indumentum adaxially	initially sparsely sericeous	initially appressed pubescent with straight hairs**^5^**, then glabrous
leaf indumentum abaxially	short dense white hairs, soon glabrous or nearly glabrous	appressed pubescent with yellowish straight hairs**^5^** (slightly silky pubescent**^3^**)
petiole length	1.3–2 cm [topotype at PH]	1–1.5(–2.5) cm**^2^**
stipule indumentum	ferrugineous appressed pubescent externally	yellowish appressed puberulous**^5^**
**flower bud length**	**0.6–1.5 cm [type specimens]**	**2–4.4 cm**
flower bud shape	narrowly ovoid or oblong-ovoid, acuminate	ovoid to cylindric**^1^**, or ellipsoid, slightly curved**^4^**
flower bud indumentum	ferrugineus-pubescent	golden appressed pilose**^4^**
tepal number	12	11–13
tepal shape	slightly acute	spathulate or oblanceolate
**tepal dimensions**	**to 25 × 4 mm**	**to 35 × 7 mm^4^**
**stamen length**	**6.5 mm**	**10–14 mm^2^**
connective.	excurrent apiculate	narrowly triangular**^5^**
**gynoecium**.	**not exceeding androecium *Kerr 4679* in flower (K: sheet 2)]**	**exceeding androecium^1^**
**gynophore length**	**ca. 3 mm**	**12 mm**
carpel hairs	short pubescent	densely sericeous-villose
style length	1.25 mm	ca. 2 mm**^6^**
**flowering period**	**December–January [Thailand] [*Kerr 4679* with flowers (K) (P)]**	**February–March [Thailand]^5^ February–April [China]^4^**
**fruiting period**	**Dec.–Jan. of next year [Thailand] [*Kerr 4679* with fruit (K) (P)]**	**April–November [Thailand]^5^ September–October [China]^4^**

Note: The listed features of *Michelia kerrii* are cited from Craib (1922: 166); with those of *Michelia floribunda* from Finet and Gagnepain (1906a: 46), supplemented by Chen and Nooteboom (1993: 1065)**^1^**, Law (1996: 60)**^2^**, Lee (1935)**^3^**. Liu et al. (2004: 254)**^4^**, Nooteboom and Chalermglin (2009: 123)**^5^** and Xia et al. (2008: 81)**^6^**.

##### Note 2.

Interestingly, apart from the initial collection, the only other record found for *Michelia kerrii* is a specimen collected at ca. 1676 m from the summit of Doi Suthep either between 11 December 1928 and January 1929 or from late December 1932 to early January 1933 by Rodolphe M. de Schauensee. This specimen includes an apparent detached flower bud bract, which would indicate that its flowering in December, a little over a decade earlier for the previous specimens, was not an isolated occurrence.

##### Note 3.

*Michelia kerrii* also does not key out with the original validating description for *Michelia microtricha* Hand.-Mazz. (Handel-Mazzetti 1921: 145) from western Yunnan, with which it shares synonymy under *M. floribunda* Dunn in Flora of China. Since *Michelia kerrii* and *M. floribunda* are obviously two distinct species, *M. kerrii* is resurrected and transferred to *Magnolia*, as has previously occurred for *Michelia floribunda*.

##### Conservation status.

*Michelia kerrii* was originally found in December 1920 at ca. 1650 m near the summit of Doi Suthep in Siam (Thailand), where it may be (or have been) endemic. As previously mentioned, the only other recorded collection for this species was by de Schauensee in the late 1920's or early 1930's from Doi Suthep’s summit. Hopefully, it still survives there or somewhere in the surrounding region after the passing of almost 100 years since it was last recorded and has not become one of several species mentioned by Elliott (1994), as being lost from Doi Suthep’s diverse flora and fauna due to the destruction of the mountain’s forest and wildlife in the rush to profit from tourism. This may be a forlorn hope in view of the statement by Putiyanan and Maxwell (2006: 175) that plant species listed for the summit in 1908 have now been “completely and shamefully removed”. Therefore, the conservation status of *Michelia kerrii* is unfortunately likely to be either critically endangered or extinct in the wild, with none known to be in *ex situ* cultivation.

##### Recommendation.

A field survey of the surrounding regions of Doi Suthep, the only recorded locality for *Magnolia kerrii* (syn. *Michelia kerrii*) in Thailand, should be undertaken as soon as possible to determine the fate of this species since its last known collection.

#### Magnolia linyaoensis

Taxon classificationPlantaeMagnolialesMagnoliaceae

(D.C. Zhang & S.B. Zhou) C.B. Callaghan & S.K. Png comb. nov.

2A403022-222D-522D-8BB6-978EDB72EB07

urn:lsid:ipni.org:names:77383217-1

Tables 16, 17

Michelia linyaoensis D.C. Zhang & S.B. Zhou, Bulletin of Botanical Research 22(2): 129, fig. 1 (Zhou and Zhang 2002). **[Basionym]**.
Michelia skinneriana Dunn, in Flora of China Vol. 7: 87 (Xia et al. 2008), p.p. quoad syn. Michelia linyaoensis D.C. Zhang & S.B. Zhou.
Michelia figo (Lour.) Spreng., in A Taxonomic Revision of the Magnoliaceae from China: 233 (Sima 2011), p.p. quoad syn. Michelia linyaoensis D.C. Zhang & S.B. Zhou.
Magnolia figo var. skinneriana (Dunn) Noot., online at World Checklist of Selected Plant Families (Govaerts 2022), p.p. quoad syn. Michelia linyaoensis D.C. Zhang & S.B. Zhou.

##### Present Chinese name.

鳞药含笑 meaning “Linyao michelia”.

##### Proposed Chinese name.

鳞药木兰 meaning “Linyao magnolia”.

##### Type.

China. **Fujian Province** • Nanping County, Laizhou town, alt. 800 m, in forest, 11 May 1998, *Zhou Shou-Biao 95544*. holotype: ANUB n.v.

Holotype specimen not found on the internet up to 22 Nov. 2022 and requests to ANUB have gone unanswered.

##### Note 1.

The present authors were unable to ascertain the location of Linyao, in the region of Laizhou of Fujian Province, after which this species is named. Also, Linyao was not recorded in the location information for the type specimen, which is unusual, as is the fact that nobody apparently returned during the autumn to collect specimens in fruit and seed for descriptive purposes and also *ex situ* conservation.

##### Note 2.

Despite Zhang and Zhou noting six features in their diagnosis distinguishing *Michelia linyaoensis* from *M. skinneriana*, it is listed as a synonym of that species in Flora of China (Xia et al. 2008) and is made a new synonym of *Michelia figo* in Sima (2011). From these species it is readily distinguished by their differentiating morphological features compiled in Tables 16, 17. *Michelia linyaoensis* also does not key out with the original validating description for *M. amoena* Q.F. Zheng & M.M. Lin, with which it shares synonymy under *M. skinneriana* Dunn in Flora of China. As an obviously distinct species, *Michelia linyaoensis* is reinstated and transferred to *Magnolia*.

**Table 16.** The **22 presently known** differentiating features between *Michelia linyaoensis* D.C. Zhang & S.B. Zhou and *Michelia skinneriana* Dunn.

**Plant feature**	***Michelia linyaoensis***	***Michelia skinneriana***
**life form**	**shrub to 2 m high**	**small tree to 15 m high^2^**
twig indumentum	densely cinnamon tomentose	densely brown villose **^2^**
bud indumentum	densely cinnamon tomentose	densely brown villose **^2^**
leaf shape	oblong or obovate-elliptic	lanceolate (narrowly obovate-elliptic, narrowly elliptic or oblanceolate**^2^**)
**leaf dimensions**	**4–7 × 1.2–2 cm**	**5–11 × 1.5–3.5 cm^2^**
leaf apex	acuminate or obtuse	long caudate-acuminate**^2^**
**leaf indumentum below**	**glabrous**.	**finely sericeous**
**lateral leaf vein pairs**	**6–8**	**8–10**
petiole indumentum	densely cinnamon tomentose	densely brown villose**^2^**
**stipules to petioles**	**not joined**	**adnate^2^**
**pedicel (peduncle) length**	**3–5 mm**	**6–8 mm**
pedicel (peduncle) indumentum	densely cinnamon tomentose	red-sericeous
**flower colour**	**white**.	**pale yellow**
tepal shape	outer: elliptic, inner: narrowly elliptic	ovate.
**tepal size**	**outer: ca. 8 × 5–6 mm inner: 3–4 mm wide**	**18–20 mm**
**tepal indumentum**	**glabrous (no hairs)**	**densely sericeous**
**stamen length**	**ca. 2 mm**	**5–6 mm**
**anther length**	**0.3 mm**	**4–5 mm^1^**
gynoecium length	ca. 5 mm	ca. 6 mm**^3^**
**gynophore length**	**ca. 2 mm**	**4–7 mm^1^**
**gynophore indumentum**	**glabrous**.	**densely brown pubescent^2^**
**distribution**.	**Nanping County, Fujian Province (endemic)**	**Zhejiang, Jiangxi, Fujian, Hunan, Guangdong, Guangxi Provinces^2^**

Note: The listed features of *Michelia linyaoensis* are cited from Zhou and Zhang (2002: 129), who record the fruit as unknown. Those of *Michelia skinneriana* are from Dunn (1908: 354), supplemented by Law (1996: 165)^1^, Liu et al. (2004: 316)^2^ and Xia et al. (2008: 87)^3^.

**Table 17.** The **21 presently known** differentiating features between *Michelia linyaoensis* D.C. Zhang & S.B. Zhou and *Michelia figo* (Lour.) Spreng.

**Plant feature**	***Michelia linyaoensis***	***Michelia figo***
**height**	**to 2 m**	**to 5 m^1^**
indumentum of twigs	densely cinnamon-coloured tomentose	densely brown tomentose (young twigs)**^1^** (densely yellow-brown pubescent**^3^**)
bud indumentum	densely cinnamon-coloured tomentose	densely yellow-brown pubescent**^3^**
leaf texture	thinly leathery	leathery**^1^**
leaf shape	oblong or obovate-elliptic	narrowly elliptic or obovate-elliptic**^1^** (elliptic to oblanceolate or obovate**^7^**)
**leaf dimensions**	**4–7 × 1.2–2 cm**	**4–10 × 1.8–4.5 cm^1,4^**
leaf apex	acuminate or obtuse	obtusely short-acute**^1^**
**leaf indumentum abaxially**	**glabrous**.	**residual appressed brown hairs along midribs^1^**
**lateral leaf vein pairs**	**6–8**	**7–12 (9–12^6^)**
petiole indumentum	densely cinnamon-coloured tomentose	densely yellow-brown pubescent**^3^**
**stipules to petioles**	**not joined**	**adnate^1^**
**pedicel length**	**3–5 mm**	**12.7 mm**
pedicel indumentum	densely cinnamon tomentose	downy**^2^**
**flower colour**	**white**.	**pale yellow or creamy white, base or margins occasionally purplish-red^1^ (shaded purple on both sides^5^)**
tepal shape	outer: elliptic. inner: narrowly elliptic	elliptic**^1^** (oblong-ovate**^7^**)
**tepal dimensions**	**outer: ca. 8 × 5–6 mm inner: 3–4 mm wide**	**12–20 × 6–11 mm^1,4^**
**stamen length**	**ca. 2 mm**	**7–8 mm^4^ (7–10 mm^7^)**
filaments.	short.	long (fig. 4**^1^**)
gynoecium length	ca. 5 mm	ca. 7 mm**^8^**
gynophore length	ca. 2 mm	3–4 mm
**gynophore indumentum**	**glabrous**.	**pale yellow tomentose^1^**

Note: The listed features of *Michelia linyaoensis* are cited from Zhou and Zhang (2002: 129), Those of *Michelia figo* are from Liu et al. (2004: 250)**^1^**, supplemented by Bean (1973: 738)**^2^**, Krüssmann (1985: 307)**^3^**, Law (1996: 165)**^4^**, Lee (1935: 484)**^5^**, Nooteboom (1985: 121)**^6^**, Spongberg (1998: 134)**^7^** and Xia et al. (2008: 87)**^8^**.

##### Conservation status.

Presently unknown, but may be critically endangered if still surviving (?). Apart from its initial publication in 2002, the only other reference to this species found by the present authors is Liu et al. (2008), wherein these Chinese authors briefly note *Michelia linyaoensis* as an endemic occurring in the subtropical evergreen broad-leaved forest(s) that occur in the northern subtropical mountainous area of China’s Zhejiang and Fujian Provinces.

##### Recommendation.

*Michelia linyaoensis*, which was collected 28 years ago and has no recorded subsequent collections, is therefore apparently known only from the type locality in Nanping County, Fujian Province, where it hopefully still survives(?). Now resurrected and disengaged from *M. skinneriana* and *M. figo*, an opportunity arises for an evaluation of its IUCN conservation status in the wild as *Magnolia linyaoensis*, which ideally should be undertaken in the not-too-distant future!

#### Magnolia longistamina

Taxon classificationPlantaeMagnolialesMagnoliaceae

(Law) C.B. Callaghan & S.K. Png comb. nov.

089DF381-CE3C-5024-BC86-73EE6F43E86E

urn:lsid:ipni.org:names:77383242-1

Table 18

Michelia longistamina Law, Bulletin of Botanical Research (Harbin) 5(3): 122, 128: fig. 2 (Law 1985). **[Basionym]**.
Michelia martini (H. Lév.) Finet & Gagnep. ex H. Lév., in Annals of Missouri Botanical Garden 80(4): 1083 (Chen and Nooteboom 1993), p.p. quoad syn. Michelia longistaminata [sic] Law plus specimen *Ko S.P. 52762* (meant as *53762*)—World Checklist and Bibliography of Magnoliaceae: 58 (Frodin and Govaerts 1996), Flora Yunnanica. Tomus 6 (Spermatophyta): 47 (Wu and Chen 2006), Flora of China Vol. 7: 88 (Xia et al. 2008), Vouchered Flora of Southeast Yunnan, Vol. 1: 62 (Sima and Lu 2009), A Taxonomic Revision of the Magnoliaceae from China: 242 (Sima 2011) and Magnoliaceae Plants of Guizhou: 172 (Deng and Yang 2015), each p.p. quoad syn. Michelia longistamina Law.
Magnolia martini H. Lév., online at World Checklist of Magnoliaceae (Govaerts 2003), p.p. quoad syn. Michelia longistamina Law.

##### Present Chinese name.

长蕊含笑 meaning “long-stamened michelia”.

##### Proposed Chinese name.

乳源木兰 meaning “Ruyuan magnolia’. (长蕊 exists in magnolia).

##### Type.

China. **Guangdong Province** • Ruyuan County, Xi Shan, Qingjidong, alt. 1300 m, tree 8m, in woodlands, 1 May 1934, *S.P. Ko* (*Gao Xi-peng*) *80358*. holotype: IBSC 0017474 n.v. isotype: BM 000574755!

**Paratypes**. China. **Guangdong Province** • Ruyuan County, Apr. 1934, *S.P. Ko* (*Gao Xi-peng*) *54122*. IBSC 0053869 n.v. **ibid**. • Nov. 1933; *Gao Xi-peng 53762*. IBSC 0053867 n.v. IBSC 0053870 n.v.

holotype and paratypes (IBSC): specimen images not uploaded to CVH at 18 Mar. 2026.

Specimen image below accessed 20 Mar. 2019:

isotype (BM): http://data.nhm.ac.uk/object/14302c91-2f98-4d41-b2a5-0d40edb0077b

##### Note 1.

Y.W. Law, in describing his new species *Michelia longistamina*, recorded in his diagnosis a number of features differentiating it from *Michelia martini* (H. Lév.) Finet & Gagnep. ex H. Lév. under which the new species (misspelt as *M. longistaminata*) was however subsequently reduced in Chen and Nooteboom (1993), where its type specimen and the type specimen of *M. bodinieri* were both noted to “strikingly resemble those of *Michelia martinii*”[sic]. Subsequent to Chen & Nooteboom, *Michelia longistamina* is recognised in Law (1996: 170) and in Liu et al. (2004: 282), but is again listed as a synonym of *M. martini* by the authors in the previously listed synonymy. However, the present authors feel that *Michelia longistamina* can be easily distinguished from *M. martini* by their presently known differentiating features compiled in Table 18.

**Table 18.** The **30 presently known** differentiating features between *Michelia longistamina* Law and *Michelia martini* (H. Lév.) Finet & Gagnep. ex H. Lév.

**Plant feature**	***Michelia longistamina***	***Michelia martini***
**life form**	**medium-sized tree to 15 m high**	**large-sized tree to 30 m high^5^**
branchlet colour	yellowish-brown when young	olive when young**^2^**
leaf bud indumentum	pale yellow patulous villose**^1^**	greyish-yellow or rufous erect long hairs**^2^**
leaf shape	elliptic, obovate-elliptic, or ovate-elliptic	oblanceolate or narrowly obovate-elliptic**^2^**
**leaf dimensions (max)**	**7–14 × 2.5–5 cm**	**12–18 × 3–5 cm^2,5^**
leaf apex	acute or short acuminate	acute or short-caudate**^2^**
leaf texture	lightly leathery	leathery**^2^**
**lateral leaf vein pairs**	**9–13**	**11–17^2,5^**
petiole length	1.3–2.5 cm	1.5–2 cm**^2,5^**
flower bud shape	elliptic.	ovoid to long ovoid**^4^**
bract indumentum	yellow villose	rufo-velutinus**^3^**
**peduncle length**	**4–14 mm**	**ca. 7 mm**
peduncle indumentum	yellow villose	densely yellowish-brown tomentose**^2^**
**tepal number**	**6**	**6–8**
**tepal colour**	**white**.	**pale yellow**
tepal shape	obovate.	obovate-oblong
**size of outer 3 tepals**	**6–7 × 4–5.5 cm**	**4–4.5 × 2–2.4 cm^2,5^**
**outer tepal apex**	**notched (figs 1, 2)^1^**	**not notched**
**stamen length**	**3–4 cm**	**1.3–1.8 cm^6^**
**anther length**	**2–2.5 cm**	**1–1.2 cm^8^**
immature carpel shape	narrowly ovate	ellipsoid-ovoid
**immature carpel length**	**ca. 4 mm**	**ca. 10 mm^6^**
ovules each carpel	8–10	8–12**^6^** (4–6**^9^**)
fruit aggregate length	8–11 cm	9–15 cm**^2,5^**
mature carpels shape	broadly ovate or suborbicular	obovoid or ellipsoid-ovoid**^2^**
mature carpels size	1.5 × 1.2 cm	1–2 cm**^6^** (1.3 × 0.8 cm**^9^**)
**flowering period**	**April–May^1^**	**February–March^2^**
**fruiting period**	**November^1^**	**August–September^2^**
altitude.	ca. 1300 m	1000–2000 m (China)**^2^** 1000–2000 (–2300) m (Vietnam)**^7^**
distribution and altitude	N Guangdong**^1^**	Guizhou, NE Yunnan, C & S Sichuan, W Hubei and S Henan**^2^** Ha Giang Province, NE Vietnam^7^

Note: The listed features of *Michelia longistamina* are cited from Law (1985: 122), supplemented by Liu et al. (2004: 282)**^1^**; with those of *Michelia martini* from Liu et al. (2004: 288)**^2^**, supplemented by Chen (1987: 89)**^3^**, Chen and Nooteboom (1993: 1083)**^4^**, Deng and Yang (2015: 172)**^5^**, Law (1996: 168)**^6^**, Nguyễn et al. (2015: 134)**^7^**, and from its description as *M. bodinieri* in Finet and Gagnepain (1906b: 574)**^9^**.

##### Note 2.

Due to the differentiating features compiled in Table 18 which show *Michelia longistamina* as an irrefutable, unambiguous species, as is also undeniably obvious from a comparison of the illustrations of the various morphological features of *Michelia longistamina* with the equivalent illustrations of *Michelia martini* in Magnolias of China (Liu et al. 2004: 282; 288), *Michelia longistamina* is here reinstated and transferred to *Magnolia*.

##### Note 3.

Accompanying the figure of a flowering branch of *Michelia martini* (H. Lév.) Finet & Gagnep. ex H. Lév. on page 103 of Flora of China Illustrations Vol. 7 (Deng 2009; fig. 103), is the obviously different flower and aggregate fruit that previously accompanied the original description of *Michelia longistamina* (Law 1985: 128, fig. 2). Both are in the 2009 publication captioned to represent *M. martini* under which *M. longistamina* is a synonym in the text volume of Flora of China.

##### Note 4.

A 3–4 cm long stamen, from whose length *Michelia longistamina* derives its name, is illustrated in Magnolias of China (Liu et al. 2004: 282 fig. 5). This is in stark contrast to the much shorter 1.3–1.8 cm long stamens of *Michelia martini* illustrated in Magnolias of China (Liu et al. 2004: 288 fig. 4 [stamen length for *M. martini* is from Law 1996: 168].

##### Note 5.

A UPGMA cluster map of genetic relationships between 48 species of *Michelia* (Li J.J. et al. 2020: 416, fig. 2), shows the closest relative of *Michelia longistamina* to be *M. xanthantha* in a sub-branch of branch V, with *M. martini* a distant relative in a sub-branch of branch I on the opposite side of the cluster map.

##### Note 6.

According to the literature searched by the present authors in Australia and overseas in relation to a future paper, *Michelia longistamina* is recorded in China for two nature reserves, as well as being noted as occurring in *ex situ* conservation at four research institutions and three botanical gardens, including South China Botanical Garden, Guangzhou, Guangdong, where M. martini* is also cultivated. [*correct ending for this Latin derived name: ICN Madrid Code, Recommendation 60C1 (Turland et al. 2025)].

##### Conservation status.

The conservation status of *Magnolia martini* H. Lév., including *Michelia longistamina* in synonymy, is noted as “Least Concern with a decreasing population trend” on the IUCN Red List of Threatened Species (IUCN 2026), based on an August 2012 assessment by S. Khela. Vietnamese researchers subsequently recommended that *Michelia martini*, occurring in at least 10 locations in China and Vietnam, should be classified as endangered EN B2b (i–v) (Nguyễn et al. 2015: 135). Two years later, Chinese authorities, utilising IUCN criteria, determined *Michelia martini* as Vulnerable, VU B1ab (i, iii) (Qin et al. 2017: 725). Obviously, the IUCN conservation status of *Magnolia longistamina* (syn. *Michelia longistamina*), recorded for only two localities as mentioned in the previous note, would be at a higher level of endangerment than was recommended by the Vietnamese researchers, but an IUCN assessment of its true situation has been obscured through being hidden as a synonym of *Magnolia martini*.

##### Recommendation.

Since it has been found that *Michelia longistamina* was assessed more than two decades ago by Chinese authorities using IUCN criteria as Critically Endangered, CR B1ab(ii,iv) + 2ab (ii,iv) (Wang and Xie 2004: 328), a re-evaluation of the current IUCN conservation status in the wild of both *Michelia longistamina* and *M. martini* independent of each other is recommended to be undertaken as soon as possible!

#### Magnolia polyneura

Taxon classificationPlantaeMagnolialesMagnoliaceae

(C.Y. Wu ex Law & Y.F. Wu) C.B. Callaghan & S.K. Png comb. nov.

770F650E-1EF6-51A2-BDBF-016564710D69

urn:lsid:ipni.org:names:77383365-1

Tables 19, 20

Michelia polyneura C.Y. Wu ex Law & Y.F. Wu, Acta Botanica Yunnanica 10(3): 340; 339 fig. 5: 1–8 (Liu & Wu [Law & Wu] 1988). **[Basionym]**.
Michelia coriacea Hung T. Chang & B.L. Chen, Annals of Missouri Botanical Garden 80(4): 1060, 1062 (Chen and Nooteboom 1993), p.p. quoad syn. Michelia polyneura C.Y. Wu ex Law & Y.F. Wu plus specimens *C.W. Wang 86915*; *88023*—World Checklist and Bibliography of Magnoliaceae: 56 (Frodin and Govaerts 1996), Flora Yunnanica. Tomus 6 (Spermatophyta): 51 (Wu and Chen 2006), Flora of China Vol. 7: 86 (Xia et al. 2008), Vouchered Flora of Southeast Yunnan, Vol. 1: 52 (Sima and Lu 2009) and A Taxonomic Revision of the Magnoliaceae from China: 244 (Sima 2011), each p.p. quoad syn. Michelia polyneura C.Y. Wu ex Law & Y.F. Wu.
Magnolia coriacea (Hung T. Chang & B.L. Chen) Figlar, online at World Checklist of Magnoliaceae (Govaerts 2003), and Yunnan Forestry Science and Technology 2001(2): 30 (Sima 2001), both p.p. quoad syn. Michelia polyneura C.Y. Wu ex Law & Y.F. Wu.

##### Present Chinese name.

多脉含笑 meaning “many-nerved michelia”.

##### Proposed Chinese name.

多脉木兰 meaning “many-nerved magnolia”.

##### Type.

China. **Yunnan Province** • Xichou County, Fadou, alt. 1500 m, in mixed forests on rocky hill, 6 m tree, 25 Sept. 1947, fr, *K.M. Feng* (*Feng Kuo-mei*) *12030*. holotype: KUN 41501 n.v. isotypes: A 0003 9060! PE 00126380! PE 00126728!

holotype (KUN): specimen image not uploaded to CVH at 18 Mar. 2026.

Specimen image below accessed 20 Mar. 2019:

isotype (A): http://kiki.huh.harvard.edu/databases/image.php?id=304834

Specimen images below accessed 20 Mar. 2019; reaccessed with new URLs 22 Oct. 2022:

isotype (PE): https://www.cvh.ac.cn/spms/detail.php?id=0073b2e4

isotype (PE): https://www.cvh.ac.cn/spms/detail.php?id=007307f9

**Paratypes** (superscript numbers at barcodes indicate appropriate URL in the subsequent list):

**Yunnan Province** • Sichour (=Xichou) County, Ma-Chia, common, alt. 1300–1500 m, 9 m tree, in mixed forests, 14 Oct. 1947, *K.M. Feng 12464*. KUN 41502 n.v. PE 00934138!^2^PE 00126379!^3^ • Guangnan County, 15 m tree, on rocky hill, 21 Mar. 1940, fl, *C.W. Wang, Chang & Y. Liu 88023*. IBK 00358058!^1^IBSC 0054436 n.v. KUN 41504 n.v. PE 00126383!^4^WUK 0268239!^9^ • Malipo County, Kwan-kao, alt. 1000 m, 12 m tree, dense woods and open slopes, 14 Feb. 1940, *C.W. Wang 86800*. KUN 41510 n.v. PE 00126382!^5^PE 00934137!^6^ • Malipo County, Guan-gaw, alt. 1200 m, small 10 m tree, occasionally amongst dense woods, 15 Feb. 1940, fl, *C.W. Wang 86915*. IBSC 0054437 n.v. KUN 41505 n.v. PE 00126385!^7^PE 00126386!^8^WUK 0268744!^10^.

Superscript numbers against URLs of paratypes match the equivalent barcodes listed previously:

paratype (IBK): https://www.cvh.ac.cn/spms/detail.php?id=f1d89f28**^1^**

paratype (PE): https://www.cvh.ac.cn/spms/detail.php?id=f00fcf6a**^2^**

paratype (PE): https://www.cvh.ac.cn/spms/detail.php?id=0073b36f**^3^**

paratype (PE): https://www.cvh.ac.cn/spms/detail.php?id=0073b1cf**^4^**

paratype (PE): https://www.cvh.ac.cn/spms/detail.php?id=0073b13d^**5**^

paratype (PE): https://www.cvh.ac.cn/spms/detail.php?id=f00fd08c^**6**^

paratype (PE): https://www.cvh.ac.cn/spms/detail.php?id=0073b022**^7^**

paratype (PE): https://www.cvh.ac.cn/spms/detail.php?id=0073af96^**8**^

paratype (WUK): https://www.cvh.ac.cn/spms/detail.php?id=bfa411ad**^9^**

paratype (WUK): https://www.cvh.ac.cn/spms/detail.php?id=bfa41249^**10**^

paratypes (IBSC, KUN): specimen images not uploaded to CVH at 18 Mar. 2026.

##### Additional material.

Specimen image not uploaded to CVH at 18 Mar. 2026.

**Yunnan Province** • alt. 1600 m; 9 Sept. 1986; *B.L. Chen GS9036*; [SYS sys00051220] n.v.

##### Note 1.

*Michelia polyneura* is noted without any given reason as a synonym of *Michelia coriacea* in Chen and Nooteboom (1993), then by the authors listed above, and as a synonym under *Magnolia coriacea* Figlar by Sima (2001). *Michelia polyneura* is a recognised species in Law (1996: 175) and Liu et al. (2004: 309) where it and *M. coriacea* are listed in different sections of subgenus *Metamichelia* Law et Y.F. Wu, with *M. polyneura* in section *Anisochlamys* Dandy and *M. coriacea* in section *Dichlamys* Dandy (Liu et al. 2004: 386; 387). According to their original descriptions, *Michelia polyneura* may be distinguished from *M. coriacea* by the presently known differentiating features compiled in Table 19 and is consequently reinstated and transferred to *Magnolia*.

**Table 19.** The **24 presently known** differentiating features between *Michelia polyneura* C.Y. Wu ex Law & Y.F. Wu and *Michelia coriacea* Hung T. Chang & B.L. Chen.

**Plant feature**	***Michelia polyneura***	***Michelia coriacea***
**life form**	**medium-sized trees to 20 m high**	**small trees to 10 m high**
bud shape indumentum	cylindric	ovoid**^3^**
bud indumentum	densely pubescent	densely tomentellous
indumentum of twigs	sparsely puberulous	dense or lax appressed puberulous when young, thence glabrous
**leaf texture**	**thinly leathery**	**thickly leathery as alluded to by epithet**
leaf shape	elliptic or elliptic-oblong	ovate, elliptic or obovate-elliptic
leaf dimensions	11–15 × 4–6 cm	12.5–14 × 2–3.5 cm
leaf apex shape	acuminate.	acute or acuminate
leaf base shape	cuneate or rounded	cuneate.
**lateral leaf vein pairs**	**17–20**	**11–14**
**petiole length**	**1–1.5 cm**	**1.8–2.3 cm**
**tepal number**	**9**	**6–7** (ex cicatricibus) **6 in photo^5^**
tepal shape	oblong or obovate-oblong	lanceolate**^5^**
**gynophore length**	**2–7 mm**	**15–18 mm**
**fruiting peduncle length**	**8–25 mm**	**9–13 mm**
fruit peduncle indumentum	yellowish pubescent**^1^** (ref. for *M. coriacea* but must refer to *M. polyneura* as listed synonym)	greyish-white appressed short pilose
fruit aggregate length	3–4 cm	to 6 cm**^3^**
**mature carpels in fruit**	**ca. 20–22**	**ca. 2–3 (–4) as most of the original 7–13 aborted**
**mature carpels colour**	**yellowish-green**	**bright red in photo^5^ or dark brown^3^**
mature carpels surface	densely lenticellate	moderately lenticellate
fragrance	very fragrant	fragrant.
flowering period	February–March**^2^**	(late February –) March–April**^5^**
**known altitudes**	**ca. 1200 m^2^ [type specimens are noted as collected 1200–1500 m]**	**1500–1700 m^5^ [1450 m for specimens collected by B.L. Chen October 1987]**
**distribution**.	**SE Yunnan**	**SE Yunnan^5^ and Bat Dai Son Nature Reserve, Ha Giang Prov., NE Vietnam^4^**

Note: *Michelia polyneura* features are from Liu and Wu (1988: 340) supplemented by Chen and Nooteboom 1993: 1063)^1^ and Liu et al. (2004: 309)**^2^**. Those of *Michelia coriacea* are from Chen (1987: 89), who records the flowers as then unknown, supplemented by Chen and Nooteboom (1993: 1063)**^3^**, Hà et al. (2020)^4^ and Liu et al. (2004: flowers in photo 238; mature carpels in photo 239)**^5^**.

##### Note 2.

The description of *Michelia polyneura* replaces the entire validating description of the earlier-named *Michelia coriacea* in Flora of China, under which it is inappropriately listed as a synonym (see Table 20).

**Table 20.** Description of *Michelia polyneura* C.Y. Wu ex Law & Y.F. Wu (1988) largely replaces description for *M. coriacea* Hung T. Chang & B.L.Chen (1987) in FOC (Flora of China).

**Plant feature**	**Original descriptions**		**Description in FOC**
	***Michelia polyneura***	***Michelia coriacea***	***Michelia coriacea***
height.	10 to 20 m	ca. 10 m	10 to 20 m
branchlet indumentum	sparsely puberulous, with greyish-yellow lenticels	densely appressed white pubescent (with greyish-white lenticels)	sparsely puberulous, with greyish-yellow lenticels
leaf shape	elliptic or elliptic-oblong	elliptic, ovate or obovate-elliptic	oblong to elliptic-oblong
leaf dimensions	11–15 × 4–6 cm	12.5–14 × 2.3–5 cm	11–15 × 4–6 cm
leaf texture	thinly leathery	leathery to thickly leathery	thinly leathery
secondary veins	17–20 pairs	11–14 pairs	17–20 pairs
petiole length	1–1.5 cm	1.8–2.3 cm	1–1.5 cm
tepal number	9	6–7	9
tepal shape	oblong or obovate-oblong	shape not noted	oblong to obovate-oblong
gynoecium length	1–1.2 cm	not noted	1–1.2 cm
gynophore length	2–7 mm	15–18 mm	2–7 mm
fruit aggregates	3–4 cm long	not noted	3–4 cm long
mature carpels	yellowish green, many	dark, almost black, few	yellowish green, many
flowering period	February–March	March–April	February–April

Note: As shown in the above table, many of the morphological features of *Michelia polyneura* have been transposed in Flora of China (Xia et al. 2008: 86) under *M. coriacea*, with none of the validating description of *M. coriacea* retained. The circumscription of *M. coriacea* in Flora of China should have been expanded by inclusion of the features of *M. polyneura* as a supposed synonym, although both have been determined as genuine independent species in the present 2-part research monograph.

##### Note 3.

The confused status of *Michelia polyneura* is as a result of the following statement by H.P. Nooteboom (Chen and Nooteboom 1993: 1063): “The type of *Michelia coriacea* bears fruit. In the absence of flowers, we misrepresented the tepals as 6–7 in number based on the faint scars of the perianth on the torus. The collections with flowers collected from the same tree one year later showed that the flower has 9 tepals”. *Chen Bao-liang & Li Biao Gs9035*, the type of *M. coriacea* without flowers, was collected at 1600 metres at Xiaoshishan in Xichou County, SE Yunnan, on 30 September 1986, with the following holotype and two isotype specimens accessed at the CVH website on 25 Mar. 2026:

holotype SYS sys00050910! https://www.cvh.ac.cn/spms/detail.php?id=7bb32632

isotype SYS: sys00095542! https://www.cvh.ac.cn/spms/detail.php?id=08f40b00

isotype SYS: sys00095543! https://www.cvh.ac.cn/spms/detail.php?id=08f40bfc

Another six specimens of *Michelia coriacea* collected the following year in October 1987 with the collection details *Chen Bao-liang 87F-177* and *Chen Bao-liang 87F-178* (5 specimen sheets) are held at the SYS herbarium, but their images are not yet posted to CVH. However, none of these would be in flower during October and they were collected from tree(s) at 1450 metres and therefore NOT from the type tree of *Michelia coriacea* located at 1600 metres. A final specimen, *Chen Bao-liang* s.n., without collection date or image, SYS: sys00050916, is noted in Chinese at CVH “without flower or fruit”. Since there are no specimens of *M. coriacea* posted to CVH for the flowering season of 1987, the question that needs to be asked is why didn’t Nooteboom give the collection details and the herbarium for these later “collections with flowers collected from the same tree” as for the type of *M. coriacea*, to rule out the distinct possibility that these 1987 specimens with 9-tepalled flowers are actually from *M. polyneura* trees which also occurs in Xichou County of Yunnan. In any case, the following three specimen sheets of *M. coriacea* that were collected on 20 February 2003 by Li Qiao-ming and Li Da-xiao from a tree in Wenshan Prefecture, Yunnan, each display flowers with 6–7 tepals:

SZG00009104! https://www.cvh.ac.cn/spms/detail.php?id=fbbf95c0

SZG00009105! https://www.cvh.ac.cn/spms/detail.php?id=fbbf965e

SZG00009106! https://www.cvh.ac.cn/spms/detail.php?id=fbbf96fa [accessed 26 Mar. 2026].

##### Note 4.

It is highly probable that the *Michelia coriacea* mentioned as “a big tree up to 30m high in the primitive patched forest in Malipo” (Sun et al. 2007: 4–5), and possibly at least some of the populations of smaller trees of ‘*Michelia coriacea*’ that are noted by these authors as occurring in the surrounding districts, are in fact *M. polyneura*, since they are considered one and the same as a result of unsubstantiated synonymy. It is evident from Table 19 that these are definitely two distinct species!

##### Note 5.

*Michelia coriacea* was noted 14 years ago as a critically endangered species occurring as scattered individuals “distributed across only three extant populations: Daping and Shipen, Dongma and Tiechang, and Yang-Kai-Ping” in SE Yunnan, with all individuals in these populations existing in degraded and fragmented habitats as a result of heavy logging and vegetation destruction (Zhao et al. 2012). Since none of the localities noted for these three remaining populations of *Michelia coriacea* are the same as the five locations where the type specimens of *Michelia polyneura* were collected in February / March 1940 and September / October 1947, this is further evidence that these are two different species.

##### Note 6.

*Michelia polyneura* is recorded under its Chinese name for the 26867 ha Wenshan National Nature Reserve, southeastern Yunnan, in two scientific surveys, but with the Latin name *Michelia coriacea* (Lu and Shui 2002: 113; Yang et al. 2008: 483), so it is not certain which of these species occurs there. (The Chinese name for *Michelia coriacea* is applied to *Michelia nitida* in both surveys).

##### Conservation status.

From an April 2007 assessment by the Global Tree Specialist Group, the conservation status of *Magnolia coriacea*, including *Michelia coriacea* and *Michelia polyneura* in synonymy, is recorded for the IUCN Red List of Threatened Species as “Endangered B1ab(iii, v) ver. 3.1 with a decreasing population trend” and as occurring in southeast Yunnan and northern Vietnam (IUCN 2026). It is therefore obvious that an independent *Magnolia polyneura* (syn. *Michelia polyneura*), which is known only to occur in nature in the three neighbouring Xichou, Guangnan and Malipo counties of SE Yunnan, would be at least as endangered. In fact, *M. polyneura* was assessed more than two decades ago by Chinese authorities using IUCN criteria as Endangered, EN B1ab(ii,iv) compared to their assessment of Endangered EN B2ab(ii,iii) for *M. coriacea* (Wang and Xie 2004: 328)., the latter assessment upgraded within 3 years to Critically Endangered CR B2ab(i,ii,iii,v) according to Sun et al. (2007). One can only guess at what their current status is now when they are no longer synonymous with each other, but it is obviously going to be much worse.

##### Recommendation.

In view of the above endangered assessments, a later assessment of *Michelia coriacea* as Vulnerable VU A2acd, B1ab(i,iii,v) by Qin et al. (2017: 725), apparently with *M. polyneura* in synonymy, is debatable. Hence an IUCN re-evaluation of the current conservation status in the wild of *Magnolia polyneura* and *M. coriacea* independent from each other should be undertaken expeditiously!

#### Magnolia skinneriana

Taxon classificationPlantaeMagnolialesMagnoliaceae

(Dunn) C.B. Callaghan & S.K. Png comb. nov.

7F8E59BE-326D-592C-B302-526F4DD9EAB0

urn:lsid:ipni.org:names:77383420-1

Michelia skinneriana Dunn, Journal of the Linnean Society of London (Botany) 38(267): 354 (Dunn 1908). **[Basionym]**.
Michelia figo (Lour.) Spreng., in Annals of Missouri Botanical Garden 80(4): 1084 (Chen and Nooteboom 1993), p.p. quoad syn. Michelia skinneriana Dunn.
Michelia figo (Lour.) Spreng. var. figo, in World Checklist and Bibliography of Magnoliaceae: 56 (Frodin and Govaerts 1996), p.p. quoad syn. Michelia skinneriana Dunn.
Michelia skinneriana Dunn, in Flora of China Vol. 7: 87 (Xia et al. 2008), p.p. excl. syns. Michelia amoena Q.F. Zheng & M.M. Lin and M. linyaoensis D.C. Zhang & S.B. Zhao.
Magnolia figo var. skinneriana (Dunn) Noot., in Flora of China Vol. 7: 49 (Xia et al. 2008).
Michelia skinneriana Dunn, in A Taxonomic Revision of the Magnoliaceae from China: 231 (Sima 2011) and Magnoliaceae Plants of Guizhou: (Deng and Yang 2015), both p.p. excl. syn. Michelia amoena Q.F. Zheng & M.M. Lin.

##### Present Chinese name.

野含笑 meaning “wild michelia”, alluding to the related *M. figo* being a cultivated plant, whereas *M. skinneriana* was found in the wild subsequent to its initial collection and botanical naming from a cultivated plant.

##### Proposed Chinese name.

野木兰 meaning “wild magnolia”.

##### Type.

China. **Fukien (= Fujian) Province** • Yenping (= Yanping District), large tree, cultivated, garden of Chinese Literary Mandarin, either May or June 1905, fl, *Stephan Troyte Dunn 2448*. holotype: HK 0000844! isotype: K 000681447! A 00039061!

The following specimen images were accessed 20 Mar. 2019:

holotype (HK): https://www.herbarium.gov.hk/en/type-specimens/search-result/index.html?quick_search=michelia_skinneriana

isotype (K: ex HK): http://apps.kew.org/herbcat/getImage.do?imageBarcode=K000681447

isotype (A: ex HK): http://kiki.huh.harvard.edu/databases/image.php?id=304835

unspecified type (SYS sys00051222): specimen image not uploaded to CVH at 18 Mar. 2026.

##### Note 1.

*Michelia skinneriana* is listed as a synonym of *Michelia figo* without any given reason in Chen and Nooteboom (1993), but subsequently is an accepted species in Law (1996: 165), Xia et al. (2008: 87), Xing et al. (2009: 211), Deng and Yang (2015: 163), Yang et al. (2016: 315) and has also been accorded specific status by Sima (2011: 231) in his dissertation revising the Chinese Magnoliaceae.

##### Note 2.

In a thesis utilising randomly amplified polymorphic DNA (RAPD) and inter-simple sequence repeat (ISSR) markers to analyse the genetic diversity and relationships of 20 Magnoliaceae species, the independence of *M. skinneriana* and *M. figo* was determined, as illustrated in RAPD and ISSR fingerprint dendrograms (Huang 2007: 35, 51; figs 14, 24).

Also, in a study which utilised AFLP and ISSR markers to determine the genetic relationship of some Magnoliaceae evergreen plants occurring in China, it was found that there was a large genetic diversity among the 21 taxa analysed, including between *M. skinneriana* and *M. figo*, and that they were completely distinguishable as independent species (Li 2013). An earlier phylogenetic analysis had also determined that *M. skinneriana* and *M. figo* could not be combined (Wang et al. 2003: 247). *Michelia skinneriana* is therefore reinstated and transferred to *Magnolia*.

##### Note 3.

According to the literature searched by the present authors in Australia and overseas, *Michelia skinneriana* is recorded in China for 57 nature reserves in nine provinces, including Anhui, Guizhou and Yunnan provinces for which this species is not mentioned as occurring in Flora of China. *Michelia skinneriana* is recorded as occurring in *ex situ* conservation at eight research institutions plus 11 botanical gardens / arboreta in China.

##### Conservation status.

From the above *Michelia skinneriana* would appear to be adequately protected in its area of distribution, with its conservation status having been listed as of Least Concern (LC) by Chinese researchers (Ministry of Environmental Protection & Chinese Academy of Sciences 2014). The present authors agree with this assessment.

#### Magnolia sublanea

Taxon classificationPlantaeMagnolialesMagnoliaceae

(Dandy) C.B. Callaghan & S.K. Png comb. et stat. nov.

A0360271-5710-5B85-B92D-447AD1330D85

urn:lsid:ipni.org:names:77383421-1

Fig. 8

Michelia macclurei var. sublanea Dandy, The Journal of Botany: British and Foreign 68(7): 212 (Dandy 1930). **[Basionym]**.
Michelia macclurei Dandy, in Annals of Missouri Botanical Garden 80(4): 1071 (Chen and Nooteboom 1993), World Checklist and Bibliography of Magnoliaceae: 58 (Frodin and Govaerts 1996), Flora Yunnanica. Tomus 6 (Spermatophyta): 50 (Wu and Chen 2006), Flora of China Vol. 7: 85 (Xia et al. 2008), A Taxonomic Revision of the Magnoliaceae from China: 210 (Sima 2011) and Magnoliaceae Plants of Guizhou: 140 (Deng and Yang 2015), each p.p. quoad syn. Michelia macclurei var. sublanea Dandy.
Magnolia mediocris (Dandy) Figlar, online at World Checklist of Magnoliaceae (Govaerts 2003), p.p. quoad syn. Michelia macclurei var. sublanea Dandy.

##### Present Chinese name.

展毛含笑 meaning “fair-haired michelia”.

**Figure 8.**
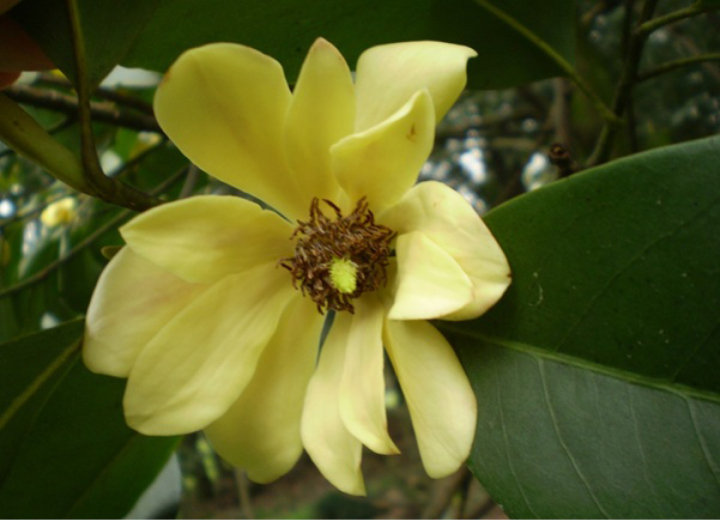
*Magnolia sublanea* (formerly Michelia macclurei var. sublanea) – photo taken by the authors at Hangzhou Botanical Garden, Yuquan, Zhejiang on 31 March 2017 **©** Callaghan & Png (ABA).

##### Proposed Chinese name.

展毛木兰 meaning “fair-haired magnolia”.

##### Type.

China. **Kwangtung (= Guangdong) Province** • Sunyi (= Xinyi County), Kyingtung (= Jindong), alt. 900 m, in the open, ground sloping west, 15 m tree, 27 July 1929, fr, *Y. Tsiang* (*Tsiang Ying*) *2609*. holotyp e: NY 00320712! isotypes: A 00039052! (ex SYS), BM 000554539! BM 000554540! IBK 00190087! IBSC 0006059 n.v. IBSC 0006060 n.v. IBSC 0006067 n.v. P 00205392! PE 00934136! SYS sys00051047 n.v.

Specimen images below accessed 20 Mar. 2019:

holotype (NY ex SYS): http://sweetgum.nybg.org/science/vh/specimen_details.php?irn=528467

isotype (A): https://s3.amazonaws.com/huhwebimages/78D742B75A914A7/type/full/39052.jpg

isotype (BM): http://data.nhm.ac.uk/object/57422918-eeb6-4472-8faf-5057ebb73929

isotype (BM): http://data.nhm.ac.uk/object/69435a3d-d4b2-4581-919d-e63fa7e08c3d

isotype (P): http://coldb.mnhn.fr/catalognumber/mnhn/p/p00205392

Specimen images below accessed 20 Mar. 2019, re-accessed with new URLs 22 Oct. 2022:

isotype (IBK): https://www.cvh.ac.cn/spms/detail.php?id=c1cdd346

isotype (PE): https://www.cvh.ac.cn/spms/detail.php?id=f00fd119

isotypes (IBSC, SYS): specimen images not uploaded to CVH at 18 Mar. 2026.

##### Note 1.

Michelia macclurei var. sublanea was reduced to *M. macclurei* in Chen and Nooteboom (1993) on the basis that the “indument varies in this species”. This treatment was followed by the authors listed above, but the variety was retained in Law (1996), Liu et al. (2004), Xing et al. (2009) and Yang et al. (2016). The present authors agree with these authors in not recognising the above synonymy. However, M. macclurei var. sublanea Dandy is raised to specific rank in recognition of the points outlined in Notes 2 and 3 below and subsequently transferred to *Magnolia* as *Magnolia sublanea* (Dandy) C.B. Callaghan & S.K. Png comb. et stat. nov.

##### Note 2.

A UPGMA cluster map of genetic relationships between 48 species of *Michelia* (Li J.J. et al. 2020: 416, fig. 2), shows the closest relative of Michelia macclurei var. sublanea to be *M. balansae* in a sub-branch of branch IV, with *M. macclurei* a distant relative in a sub-branch of branch I on the opposite side of the cluster map and consequently not a variety of the earlier-named species.

##### Note 3.

**Figure 9.**
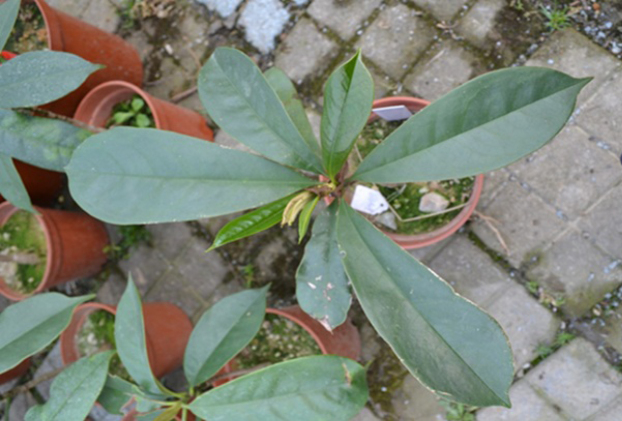
A *Magnolia calcarea* (ex *Manglietia*) rooted cutting. *Ex situ* conservation starts in the nursery! Photo taken at Hangzhou Botanical Garden, Yuquan, Zhejiang on 31 March 2017 © Callaghan & Png (ABA).

According to its brief description in Magnolias of China (Liu et al. 2004: 286), Michelia macclurei var. sublanea is noted to differ from *Michelia macclurei* in its young twigs, petioles, stipules, bracts and peduncles being densely patulous (not appressed) tomentellous or villose and with is leaves and flowers slightly larger. Overlooked in these differences is the variety’s 10–14 tepals shown at fig. 1 on page 286 of Magnolias of China (13 tepals at Fig. 8, page 130 of this paper), its white stamens with red pollen sacs shown at Figs 8, 9 on the same page (which apparently turn brown after pollen release) and its pendulous fruit aggregates shown in the lower L-H photo on page 287. These are in stark contrast to the 9 tepals mentioned in the text and shown at fig. 1 on page 284 for *Michelia macclurei*, as well as its red stamens with red pollen sacs shown at figs 6, 7 on the same page, plus the upright fruit aggregates shown in the upper R-H photo on page 285 of Magnolias of China. Also not mentioned are the 5–6 ovules in respect of M. macclurei var. sublanea (Dandy 1928b: 360) and the 6–9 ovules in respect *M. macclurei* (Dandy 1930: 212). Specimens posted to the CVH website show the flowering months of the variety to range from February to April and its fruiting months to range from June to November compared to February to March and October to November for the flowering and fruiting months noted in Magnolias of China for *M. macclurei*.

##### Conservation status.

The conservation status of *Magnolia macclurei*, including Michelia macclurei var. sublanea as a synonym, is presently noted as “Least Concern with an unknown population trend” on the IUCN Red List of Threatened Species, based on an August 2012 assessment (IUCN 2026).

##### Recommendation.

A field survey at the type locality of Michelia macclurei var. sublanea, plus the nature reserves in which it occurs, plus any other confirmed wild locations for this now former variety, such as Maoming, Guangdong and Yulin, Guangxi (Law 1996), should be undertaken to ascertain its current IUCN Red List status as *Magnolia sublanea*.

#### Magnolia tenuipes

Taxon classificationPlantaeMagnolialesMagnoliaceae

(Dandy) C.B. Callaghan & S.K. Png comb. nov.

50B019A6-B656-530D-A90F-C427B90B6435

urn:lsid:ipni.org:names:77383475-1

Tables 21, 22

Manglietia tenuipes Dandy, The Journal of Botany: British and Foreign 69(9): 232 (Dandy 1931). **[Basionym]**.
Manglietia conifera Dandy, in Annals of Missouri Botanical Garden 80(4): 1034 (Chen and Nooteboom 1993), World Checklist and Bibliography of Magnoliaceae: 50 (Frodin and Govaerts 1996), online at World Checklist of Magnoliaceae (Govaerts 2003), in Flora Yunnanica. Tomus 6 (Spermatophyta): 6 (Wu and Chen 2006), Flora of China Vol. 7: 60 (Xia et al. 2008), Vouchered Flora of Southeast Yunnan, Vol. 1: 20 (Sima and Lu 2009), A Taxonomic Revision of the Magnoliaceae from China: 78 (Sima 2011) and Magnoliaceae Plants of Guizhou: 19 (Deng and Yang 2015), each p.p. quoad syn. Manglietia tenuipes Dandy.
Manglietia chingii Dandy, in Flora Reipublicae Popularis Sinicae 30(1): 92 (Law 1996), p.p. quoad syn. Manglietia tenuipes Dandy.
Manglietia conifera subsp. chingii (Dandy) J. Li, in Acta Botanica Yunnanica 19(2): 131 (Li 1997), p.p. quoad syn. Manglietia tenuipes Dandy.

##### Present Chinese names.

广西木莲 meaning “Guangxi manglietia” and 细柄木莲 meaning “thin-stalked manglietia”.

##### Proposed Chinese name.

细柄木兰 meaning “thin-stalked magnolia”.

##### Type.

China. **Kwangsi (= Guangxi Zhuang Autonomous Region)** • Yeo Mar Shan (= Yaoma Shan), north of Lin Yen (= Lingyun), alt. ca. 1460 m, in woods, common, tree 12.2 m high, 25 Aug. 1928, fr, *Ching Ren-chang 7117*. holotype: BM 000946043! isotypes: IBSC 0006058! (incorrectly noted as Nov. 3, 1928 on annotation label), NY 00320705! NAS 00319372! NAS 00070747! PE 00934127! SYS sys00051814 n.v.

##### Note.

Yaoma Shan (瑶马山) is about 7.5 km west of the current day county seat of Lingyun.

Specimen image below accessed 1 Apr. 2020:

holotype (BM): http://plants.jstor.org/stable/10.5555/al.ap.specimen.bm000946043

Specimen image below accessed 20 Mar. 2019:

isotype (NY): http://sweetgum.nybg.org/science/vh/specimen_details.php?irn=403451

Specimen images below accessed 20 Mar. 2019; re-accessed with new URLs 22 Oct. 2022:

isotype (IBSC): https://www.cvh.ac.cn/spms/detail.php?id=c7677f91

isotype (NAS): https://www.cvh.ac.cn/spms/detail.php?id=dbe4a4ba

isotype (NAS): https://www.cvh.ac.cn/spms/detail.php?id=da7e2b07

isotype (PE): https://www.cvh.ac.cn/spms/detail.php?id=f00fd5fc

isotype (SYS): specimen image not uploaded to CVH at 18 Mar. 2026.

##### Note 1.

An identification label attached to the isotype specimen at the New York Botanical Garden herbarium by Chen Bao-liang in 1990 makes the determination as *Manglietia conifera* Dandy. *Manglietia tenuipes* is listed as a synonym of *M. conifera* in Chen and Nooteboom (1993), being subsequently recorded as such by the authors noted above. It is also recorded as a synonym of *M. chingii* in Law (1996) and of M. conifera subsp. chingii by Li (1997). *M. tenuipes* is, however, recognisable as a distinct species when taking into account all the presently known differentiating features of these three species compiled in Tables 21, 22. *Manglietia tenuipes* also does not key out with the original validating descriptions of *M. glaucifolia* Law & Y.F. Wu, or *M. ovoidea* Hung T. Chang & B.L. Chen with which it shares synonymy under *M. conifera* in Chen & Nooteboom, and with the exception of *M. glaucifolia*, also in Flora of China.

**Table 21.** The **21 presently known** differentiating features between *Manglietia tenuipes* Dandy and *Manglietia conifera* Dandy.

**Plant feature**	***Manglietia tenuipes***	***Manglietia conifera***
**life form**	**small tree to ca. 12 m high^2^**	**tree to 30 m high^5^, cultivated trees to mean height of 24.7 m in ca. 16 years^3^**
twig indumentum	appressed coppery tomentelli initially, then more or less glabrescent	densely reddish pubescent when young
leaf dimensions	to ca. 20 × 4.5 cm	to ca. 20 × 7 cm
leaf apex	gradually acuminate, near to acute	more or less acuminate or obtuse
lateral leaf vein pairs	ca. 15–22	ca. 14–18
**petiole length**	**1.4–3.5 cm**	**1.1–2.6 cm**
**petiole indumentum**	**glabrous^2^**	**hairy^4^**
stipular scars on petiole	3–7 mm**^2^**	5–8 mm**^4^**
tepal number	11	10–11
**tepal length**	**3–3.6 cm^2^**	**2.5–3 cm^4^**
**staminal scars on central axis**	**3–5 mm long region of attachment**	**6–8 mm long region of attachment**
**stamen length**	**10–11 mm^2^**	**8.5–10 mm^4^**
**immature carpel number**	**26–38**	**15–25**
**carpel dimensions**	**3–3.4 × 2–3 mm**	**2.2–3 × 2.2–3 mm^4^**
ovules each carpel	4–10**^2^**	4–12**^4^**
**fruit peduncle length**	**ca. 4.5–5.5 cm (ca. 6 cm^1^)**	**up to 4 cm**
**fruit peduncle indumentum**	**glabrous**.	**appressed-pubescent at least when young**
fruit aggregate shape	ovoid-globose to oblong-globose	broadly ovoid
**fruit dimensions**	**ca. 6 × 4.5 cm^1^**	**3.2–3.5 × 2.8–3.4 cm^4^**
**altitude**.	**1460 m**	**700–1300 m^4^**
**distribution**.	**Yaoma Shan, Lingyun County, NW Guangxi Province, China**	**Bavi, Dam Ha, Ha Coi, Son Tay and Tam Đảo, northern Vietnam^4^**

Note: The morphological features of *Manglietia tenuipes* are cited from Dandy (1931: 232), supplemented by Lee (1935: 479)**^1^** and Tiêṕ (1980: 557)**^2^**, with those of *Manglietia conifera* cited from Dandy (1930: 205), supplemented by Hao et al. (2019: 3)**^3^**, Tiêṕ (1980: 559–560)**^4^** and Vu (2017: 1190)**^5^**.

**Table 22.** The **20 presently known** differentiating features between *Manglietia tenuipes* Dandy and *Manglietia chingii* Dandy.

**Plant feature**	***Manglietia tenuipes***	***Manglietia chingii***
**life form**	**small-sized tree to ca. 12 m high^2^**	**medium-sized tree to 20 m high^4^**
twig indumentum	appressed coppery tomentelli initially, then more or less glabrescent	initially appressed coppery red pubescent, then glabrous
**leaf dimensions**	**to ca. 20 × 4.5 cm**	**to ca. 15 × 5 cm^4^**
leaf apex	gradually acuminate, near to acute	short-acuminate or obtuse**^4^**
lateral leaf vein pairs	ca. 15–22	14–20
venation pattern	indistinct abaxially	distinct abaxially
**petiole length**	**1.4–3.5 cm**	**1.6–2.2 cm**
petiole indumentum	glabrous**^2^**	glabrescent, then glabrous**^5^**
**stipular scar length on petiole**	**3–7 mm^2^**	**2–4 mm^5^**
tepal number	11	9–11
**tepal length**	**3–3.6 cm^2^**	**5–5.5 cm^4^**
**staminal scars on central axis**	**3–5 mm long region of attachment**	**7–9 mm long region of attachment**
**stamen length**	**10–11 mm^2^**	**13–15 mm^3^**
**immature carpels**	**26–38**	**28–30**
number of ovules	4–10**^2^**	8–10 **^5^**
**fruit peduncle length**	**ca. 4.5–5.5 cm (ca. 6 cm^1^)**	**6.5–7 cm**
fruit aggregates size	ca. 4.5–5 cm long (ca. 6 × 4.5 cm**^1^**)	ca. 4–5 cm long (ca. 5 × 3.3 cm**^5^**)
fruit aggregates shape	ovoid-globose to oblong-globose	ovoid to ellipsoid-oblong
**altitude**.	**1460 m**	**790 m**
**distribution**.	**Yaoma Shan, Lingyun County, NW Guangxi Province, China**	**Shiwandashan, S. Guangxi Province (and Fu-ming, Yunnan Province^5^), China**

Note: The morphological features of *Manglietia tenuipes* are cited from Dandy (1931: 232), supplemented by Lee (1935: 479)**^1^** and Tiêṕ (1980: 557)**^2^**. Those of *Manglietia chingii* are from Dandy (1931), supplemented by Law (1996: 92)**^3^**, Liu et al. (2004: 130)**^4^** and Tiêṕ (1980: 559)**^5^**.

##### Note 2.

In research published five years ago in which a UPGMA cluster analysis supported the division of Magnoliaceae into the two monogeneric subfamilies Magnolioideae and Liriodendroideae, there is a UPGMA clustering diagram of genomic relationships between 135 species of Magnoliaceae (Li 2021: 30, fig. 3.6). This diagram reveals the closest relative to *Manglietia tenuipes* to be *M. ovoidea*, with *M. conifera* and *M. chingii* distant relatives on the genome map. Previously published research utilising ISSR molecular markers to investigate the genetic diversity of 48 wild species of Magnoliaceae in Yunnan Province showed *Manglietia tenuipes* to be not closely related to *Manglietia chingii* on the accompanying UPGMA cluster map (Wang et al. 2020: 281, fig. 2). Recognised as a genuine species, *Manglietia tenuipes* is reinstated and transferred to *Magnolia*.

##### Note 3.

While there are cultivated plants of *Manglietia conifera* growing in the Magnolia Garden at South China Botanical Garden (from which herbarium specimens are noted but not uploaded at the Chinese Virtual Herbarium website) and planted elsewhere in China, this species does not occur in the wild in China, but is confined to Vietnam (Hao et al. 2019: 1; 2 fig. 1), where for example it was recorded in 1997 for the Na Hang Nature Reserve in northeastern Tuyen Quang Province (Hill and Hallam 1997: appendix 1 Plants), whose establishment and area of 41930 ha was first proposed in October 1993 (Anon 2004). The majority of the specimens presently at the CVH website bearing the name *Manglietia conifera* have the Chinese name for *M. chingii* under which they were presumably posted and some are neither. Also, research of the genetic diversity of the germplasm resources of *M. conifera* required 12 wild provenances of this species being introduced to China from Vietnam (Wen 2017). The naming of the superficially similar but different species *M. sinoconifera* (Wei 1993), would appear to be further evidence that *M. conifera*, which is conspicuous for its absence in Flora Reipublicae Popularis Sinicae and Magnolias of China, is not native to China, whereas *M. tenuipes* and perhaps also *M. chingii* apparently occur only in China.

##### Note 4.

According to the literature searched by the present authors in Australia and overseas for a future paper, *Manglietia tenuipes* is recorded in China for 18 nature reserves, two at least in which one of its supposed synonyms *M. chingii* is also noted to occur. *M. tenuipes* is also recorded as occurring in *ex situ* conservation at two research institutions plus eight botanical gardens and arboreta, four in which *M. chingii* is also noted to occur, including South China Botanical Garden, Guangzhou, Guangdong, where in addition to these two species, *M. conifera* is also cultivated.

##### Conservation status.

*Manglietia tenuipes* is noted on the IUCN Red List of Threatened Species as a synonym of *Magnolia conifera* (Dandy) V.S. Kumar which has a September 2012 assessment of “Least Concern” (IUCN 2026). However, *Manglietia tenuipes* is recorded by Li and Li (1999: 33) as being listed as a level 3 provincial key protected plant on the Yunnan Key Protected Wild Plants List published in 1989, and in Li et al. (2015: 72) it is noted as a national level 3 protected rare and endangered plant.

##### Recommendation.

In view of the above, an IUCN assessment of the present conservation status in the wild of *Magnolia tenuipes* (syn. *Manglietia tenuipes*) independent of *Magnolia conifera* and Magnolia conifera var. chingii (Dandy) V.S. Kumar (syn. *Manglietia chingii*), needs to be undertaken at the earliest opportunity! Included in the IUCN assessment should be populations in Funing and Malipo counties of Wenshan Zhuang and Miao Autonomous Prefecture, Yunnan (from where unsighted specimens of *M. tenuipes* were collected by Chen Bao-liang), plus at Leigong Shan and elsewhere in Guizhou, as well as a number of isolated Guangxi populations, including at the type locality of Yaoma Shan in Lingyun County.

#### Magnolia wardii

Taxon classificationPlantaeMagnolialesMagnoliaceae

(Dandy) C.B. Callaghan & S.K. Png comb. nov.

9915F2F7-4000-5CFA-972A-05B47D1B3102

urn:lsid:ipni.org:names:77383523-1

Table 23

Michelia wardii Dandy, Kew Bulletin of Miscellaneous Information 1929(7): 222–223 (Dandy 1929a). **[Basionym]**.
Michelia doltsopa Buch.-Ham. ex DC., in Annals of Missouri Botanical Garden 80(4): 1063 (Chen and Nooteboom 1993), World Checklist and Bibliography of Magnoliaceae: 56 (Frodin and Govaerts 1996), Flora Yunnanica. Tomus 6 (Spermatophyta): 41 (Wu and Chen 2006), Flora of China Vol. 7: 81 (Xia et al. 2008), Vouchered Flora of Southeast Yunnan, Vol. 1: 53 (Sima and Lu 2009), A Taxonomic Revision of the Magnoliaceae from China: 197 (Sima 2011) and Magnoliaceae Plants of Guizhou: 131 (Deng and Yang 2015), each p.p. quoad syn. Michelia wardii Dandy.
Magnolia doltsopa (Buch.-Ham. ex DC) Figlar, online at World Checklist of Magnoliaceae (Govaerts 2003), and in Phytotaxonomy 15: 109 (Khuraijam and Goel 2015), both p.p. quoad syn. Michelia wardii Dandy.

##### Type.

Ne India – **Arunachal Pradesh** • Chibaon, Delei Valley, 28°10'N, 96°30'E, alt. 2100–2400 m, 12 Apr. 1928, fl, *Francis Kingdon-Ward 8060*. holotype: K 000681496! isotypes: BM n.v. E n.v. NY n.v.

holotype specimen below accessed 20 March 2019:

holotype (K): http://apps.kew.org/herbcat/getImage.do?imageBarcode=K000681496

##### Note 1.

*Michelia wardii* Dandy is noted as a synonym of *Michelia doltsopa* Buch.-Ham. ex DC. in Chen and Nooteboom (1993) and then by the other authors listed above. However, it can be distinguished from that species by the presently known differentiating features compiled in Table 23.

**Table 23.** The **26 presently known** differentiating features between *Michelia wardii* Dandy and *Michelia doltsopa* Buch.-Ham. ex DC.

**Plant feature**	***Michelia wardii***	***Michelia doltsopa***
size	large trees (“magna” - K.W.)	medium to large trees (to 30 m**^6^**)
**seasonal nature**	**semi-deciduous**	**evergreen**.
indumentum of twigs	grey pubescent at nodes when young, otherwise glabrous	rufous or greyish-white appressed pubescent when young**^6^**, eventually glabrescent**^10^**
leaf shape	oblanceolate, narrowly oblong, or narrowly elliptic-oblong	elliptic, narrowly ovate-elliptic or oblong-elliptic**^6^**
**leaf size**	**up to ca. 16 × 4 cm maximum**	**up to 22.5 × 7.5 cm^1^ (up to 22 × 7 cm^6^)**
leaf apex	acuminate or occasionally acute	short-acute or long-acute**^6^**
leaf base	decurrent tapering on to petiole	broadly cuneate or obtuse**^6^**
lateral leaf vein pairs	ca. 9–14	mostly more than 12**^9^**
leaf texture	papery.	thinly leathery**^6^** (thick, leathery**^5^**) (+/– leathery**^10^**)
leaf indumentum abaxially	glabrous or sparsely grey appressed pubescent mainly on midrib towards the apex	rufous or greyish-white appressed pubescent when young**^6^**, with orange pubescent veins**^7^**
**petiole length**	**ca. 1.5 cm**	**2–2.4 cm^1^**
petiole indumentum	grey appressed pubescent near base when young	rufous or greyish-white appressed pubescent**^6^**
indumentum of stipules externally	greyish appressed tomentose or appressed pubescent	rufous or tawny pubescent**^3^** dark brown velvety**^1^**
indumentum of spathoid bracts	densely greyish silky tomentose	rufous or tawny pubescent**^3^** orange-rusty hairs on pale green scales**^7^**
**flower peduncle length**	**ca. 5–7 mm**	**4–13(–23) mm^2^**
flower peduncle indumentum	densely grey tomentose	densely dark brown-cinnamon coloured tomentum**^1^** densely rufous**^10^** appressed villose**^6^**
**tepal number**	**ca. 9–12**	**12–16^6,8^**
**tepal colour**	**cream**.	**white**.
tepal shape	obovate or oblanceolate-oblong	ovate-oblong**^1^** (narrowly obovate-spathulate**^6^**)
tepal length	ca. 4.5–6.5 cm (exterior tepals)	5–7 cm**^6^** (exterior tepals)
**tepal indumentum**	**pubescent on outside near base**	**glabrous^1^**
stamen length	ca 10–15 mm	12–17 mm**^4^**
gynoecium shape	subcylindric.	narrowly ovoid**^6^**
fruiting peduncle indumentum	dense greyish tomentose (Frank Kingdon-Ward’s field notes)	rufous or tawny appressed pubescent**^3^**
**altitude within China**	**2100–2400 m**	**1500–2300 m^6^**
distribution within China	28°10'N, 96°30'E, Delei Valley, Xizang (Tibet) and NE India	Gongshan, Yunnan and Xizang (Tibet)^6^

Note: The differentiating morphological features of *Michelia wardii* are cited from Dandy (1929a: 222) and those of *Michelia doltsopa* from de Candolle (1818: 448)^1^, supplemented by Chen and Nooteboom (1993: 1063)^2^; Dandy (1929a: 222)^3^, Law (1996: 159)^4^, Lee (1935)^5^, Liu et al. (2004: 242)^6^, Mitchell and Coombes (1998: 181)^7^, Polunin and Stainton (1999: 19)^8^, Spencer (1997: 23)^9^ and Spongberg (1998: 135)^10^.

##### Note 2.

*Michelia wardii* also does not key out with the original validating descriptions for *M. opipara* Hung T. Chang & B.L. Chen or the at least semi-deciduous *M. excelsa* (Wall.) Blume, occurring in the Himalayas from Nepal eastward (Brandis 1906: 7), with which it shares synonymy under *M. doltsopa* in Chen & Nooteboom and in Flora of China. *Michelia wardii* is therefore resurrected and transferred to *Magnolia*.

##### Note 3.

*Michelia wardii* Dandy is native to the northeast Indian state of Arunachal Pradesh and also adjacent Xizang (Tibet) in far-western China. No records of it in any nature reserve in these regions or in cultivation have been located.

##### Conservation status.

Due to being regarded as a synonym of *Magnolia doltsopa* since 1993, no information on the conservation status of *M. wardii* has been found in the literature.

##### Recommendation.

Utilising the co-ordinates noted by Kingdon-Ward as a starting point, an IUCN assessment of any existing wild populations of *Magnolia wardii* (syn. *Michelia wardii*) surviving in the Delai Valley, Mishmi Hills region, Chibaon, Arunachal Pradesh, India should be undertaken to ascertain its conservation status now that is has been shown to be independent of *Magnolia doltsopa*.

## Supplementary Material

XML Treatment for Magnolia balansae var. appressipubescens

XML Treatment for Magnolia brevipes

XML Treatment for Magnolia calcarea

XML Treatment for Magnolia calcicola

XML Treatment for Magnolia chingii

XML Treatment for Magnolia congjiangensis

XML Treatment for Magnolia floribunda var. lanea

XML Treatment for Magnolia forrestii

XML Treatment for Magnolia fulgens

XML Treatment for Magnolia glaberrima

XML Treatment for Magnolia hainanensis

XML Treatment for Magnolia hedyosperma

XML Treatment for Magnolia kerrii

XML Treatment for Magnolia linyaoensis

XML Treatment for Magnolia longistamina

XML Treatment for Magnolia polyneura

XML Treatment for Magnolia skinneriana

XML Treatment for Magnolia sublanea

XML Treatment for Magnolia tenuipes

XML Treatment for Magnolia wardii

## Appendix 1 - Institutional herbarium acronyms

The acronyms of herbaria where types and other specimens are deposited are as follows:

**A** Herbarium of the Arnold Arboretum (Harvard University Herbaria), Harvard University, Cambridge, Massachusetts, U.S.A.

**ANUB** Department of Biology Herbarium, Anhui Normal University, Wuhu, Anhui, China.

**AU** Herbarium, Biology Department, Xiamen University, Xiamen, Fujian, China.

**BM** Department of Botany Herbarium, British Museum of Natural History, London, England, U.K.

**CAF** Dendrological Herbarium, Chinese Academy of Forestry, Haidian, Beijing, China.

**CCC** Herbarium, Canton Christian College, Lingnan University, Canton (Guangzhou), Guangdong, China. (incorporated into Sun Yat-sen University in 1952 – see SYS).

**CQNM** Herbarium, Chongqing Natural History Museum, Chongqing City, Chongqing Municipality, China.

**E** Royal Botanic Garden Herbarium, Inverleith Row, Inverleith, Edinburgh, Scotland, U.K.

**FJFC** Dendrological Herbarium, Fujian Forestry College, Nanping, Fujian, China. (now College of Forestry, Fujian Agriculture and Forestry University, Fuzhou, Fujian).

**GAC** Forestry Herbarium, Forestry Department, Guangxi Agricultural University, Nanning, Guangxi, China.

**GFS** Dendrological Herbarium, Guizhou Forestry School, Xiuwen, Guiyang, Guizhou, China.

**GNUG** Herbarium, Geography Department, Guizhou Normal University, Guiyang, Guizhou, China.

**GZAC** Dendrological Herbarium, Forestry Department, Guizhou Agricultural College, Huaxi, Guiyang, Guizhou, China.

**H** Botanical Herbarium, Finnish Museum of Natural History, University of Helsinki, Helsinki, Finland.

**HEAC** Henan Agricultural University Herbarium, Zhengzhou, Henan, China.

**HEBI** Institute of Biology Herbarium, Henan Academy of Sciences, Zhengzhou, Henan, China.

**HGAS** Institute of Biology Herbarium, Guizhou Academy of Sciences, Xiaohe, Yunyang District, Guiyang, Guizhou, China.

**HIB** Herbarium, Wuhan Institute of Botany, Chinese Academy of Sciences, Moshan, Wuchang District, Wuhan, Hubei, China.

**HITBC** Xishuangbanna Tropical Botanical Garden Herbarium, Chinese Academy of Sciences, Menglun, Mengla County, Yunnan, China

**HK** Hong Kong Botanical Garden Herbarium, Kowloon, Hong Kong, China.

**HSBL** Herbarium of Biological Laboratory of the Science Society of China, Nanking (= Nanjing), Jiangsu, China. (transferred to NAS Herbarium)

**HTC** Herbarium, Department of Biology, Hangzhou Normal University, Hangzhou, Zhejiang, China.

**IBK** Herbarium, Guangxi Institute of Botany, Guangxi Academy of Sciences, Yanshan District, Guilin, Guangxi Zhuang Autonomous Region, China.

**IBSC** Department of Taxonomy Herbarium, South China Institute of Botany (SCBI), Chinese Academy of Sciences, Tianhe District, Guangzhou, Guangdong, China.

**JXAU** Dendrological Herbarium, Department of Forestry, Jiangxi Agricultural University, Meiling, Nanchang, Jiangxi, China.

**K** Royal Botanic Gardens Herbarium, Kew, Surrey, London, England. U.K.

**KUN** Herbarium, Kunming Institute of Botany, Chinese Academy of Sciences, Heilongtan, Panlong District, Kunming, Yunnan, China.

**LBG** Herbarium, Lu Shan Botanical Garden, Hanpokou, Lu Shan, Jiangxi, China.

**LE** Vascular Plants Herbarium, Komarov Botanical Institute of the Russian Academy of Sciences, Saint Petersburg, Russia.

**LU** Herbarium of Lingnan Natural History Survey and Museum, Lingnan University, Canton (Guangzhou), Guangdong, China. (transferred to SYS).

**MO** Herbarium, Missouri Botanical Garden, Saint Louis, Missouri, USA.

**NAS** Herbarium, Phytotaxonomy Department, Jiangsu Institute of Botany, Qianhuhou, Zhongshanmenwai, Nanjing, Jiangsu, China.

**NF** Dendrological Herbarium, College of Forest Resources and Environment, Nanjing Forestry University, Nanjing, Jiangsu, China. (ex-Nanjing Forestry College)

**NHN/L** National Herbarium of the Netherlands, Rijksherbarium, Leiden University, Leiden, Netherlands.

**NKU** Herbarium, Plant Biology and Ecolgy Department, Nankai University, Nankai District, Tianjin, China.

**NY** William and Lynda Steere Herbarium, New York Botanical Garden, The Bronx, New York, U.S.A.

**P** Herbarium National de Paris, Muséum National d’Histoire Naturelle, Paris, France.

**PE** Herbarium, Laboratory of Systematic and Evolutionary Botany, Institute of Botany, Chinese Academy of Sciences, Nanxin, Xiangshan, Beijing, China.

**PH** Herbarium, Department of Botany, Academy of Natural Sciences, Drexel University, Philadelphia, Pennsylvania, U.S.A.

**PL** Botanické Herbarium, Západoceské Muzeum, Plzeň-City (Pilsen), Czech Republic.

**SHM** Herbarium, Department of Botany, Shanghai Museum of Natural History, Jing’an District, Shanghai, China.

**SYS** Botanical Division Herbarium, Biology Department, Zhongshan University (Sun Yat-sen University), Haizhu District, Guangzhou, Guangdong, China.

**SZ** Herbarium, Department of Biology, Sichuan University, Jiuyanqiao, Chengdu, Sichuan, China.

**SZG** Herbarium, Fairy Lake Botanical Garden, Shenzhen, Guangdong, China.

**TCD** Herbarium, School of Botany, Trinity College, Dublin, Ireland.

**UC** Herbarium, University of California, Berkeley, California, U.S.A.

**US** National Herbarium, Department of Botany, National Museum of Natural History, Smithsonian Institution, Washington, DC, U.S.A.

**WUK** Herbarium, Northwestern Institute of Botany, Yangling, Xianyang, Shaanxi, China.

**YAF** Herbarium, Yunnan Academy of Forestry, Heilongtan, Kunming, Yunnan, China.

Herbaria references

The above herbarium acronyms and their institutions were located in the following publications:

Chen SC, Li JL, Zhu XY, Zhang ZY (eds) 1990. Chinese Herbaria and also Herbarium Abbreviations. In: Bibliography of Chinese Systematic Botany (1949–1990): 667–684; 685–698. Guangdong Science & Technology Press, Guangzhou. (In Chinese and English).

Fu LK, Zhang XC, Qin HN, Ma JS (eds) 1993. Index Herbariorum Sinicorum. China Science and Technology Press, Beijing. (In Chinese and English).

Holmgren PK, Holmgren NH, Barnett LC (eds) 1990. Index Herbariorum. Part 1. The Herbaria of the World. Eighth Edition. New York Botanical Garden, The Bronx, New York, on behalf of the International Association for Plant Taxonomy, Bratislava, Slovakia.

Jin SY, Chen YL (eds) 1994. A Catalogue of Type Specimens (Cormophyta) in the Herbaria of China. Science Press, Beijing. (In Chinese: Magnoliaceae 453–457). Two supplements published in 1999 and 2007 have not been seen.

Thiers BM (ed.) Index Herbariorum. A searchable online facility of the world’s herbaria utilising the required herbarium’s acronym to provide a collections summary and also lists of the herbarium’s correspondent(s), plus their staff and research associates. http://sweetgum.nybg.org/science/ih/ [accessed as required from 27 Apr. 2016].

## Additional information

### Artificial Intelligence (AI) use

The authors accept full responsibility for the content of the manuscript, including the disclosure of any use of AI.

No AI tools were used in the preparation of this manuscript.

### Author ORCIDs

C.B. Callaghan https://orcid.org/0009-0001-3866-7412

S.K. Png https://orcid.org/0009-0005-4744-8296

### Data availability

All of the data that support the findings of this study are available in the main text.

## References

[B1] Andrews HC (1802) The Botanist’s Repository, Comprising Colour’d Engravings of New and Rare Plants Only. Vol. IV. Published by the author. T. Bensley (printers), London. [*Magnolia fuscata* Andr., plate 229, with Latin description]. https://biodiversitylibrary.org/page/35238599 [accessed 30 Mar. 2020].

[B2] Anon (2004) Sourcebook of Existing and Proposed Protected Areas in Vietnam, Second Edition. Birdlife International in Indochina and the Forest Inventory and Planning Institute of the Vietnamese Ministry of Agriculture and Rural Development (MARD). Ba Na Nui Chua Nature Reserve: https://thiennhienviet.org.vn/sourcebook/pdf/1%20South%20Central%20Coast/Ba%20Na-Nui%20Chua.pdf Na Hang Proposed Nature Reserve: https://thiennhienviet.org.vn/sourcebook/pdf/2%20north%20east/Na%20Hang.pdf [both accessed 23 Mar. 2026]

[B3] Anon (2012) The State of China’s Forest Genetic Resources. Main Report. Beijing, China. [prepared under the auspices of the U.N. Food and Agriculture Organisation]. http://www.fao.org/3/i3825e/i3825e13.pdf [accessed 7 Apr. 2020]

[B4] Anon (2022) *Magnolia laevifolia* syn. *Michelia yunnanensis*. (Culture and Noteworthy Characteristics). Missouri Botanical Garden Plant List. https://www.missouribotanicalgarden.org/PlantFinder/PlantFinderDetails.aspx?taxonid=445852&isprofile=0&cv [accessed 12 Oct. 2022]

[B5] Bean WJ (1973) Trees and Shrubs Hardy in the British Isles. Vol. II, D–M. John Murray (publishers) Ltd., London. http://www.beanstreesandshrubs.org/ [accessed 30 Mar. 2020]

[B6] Brandis D (1906) Indian Trees. Archibald Constable & Co. Ltd., London. [*Manglietia caveana* p. 6, *Michelia excelsa* pp. 7–8]. https://www.biodiversitylibrary.org/page/34178947 [accessed 30 Mar. 2020]

[B7] Callaghan C (2012) *Cathaya argyrophylla*, some little-known facts. International Dendrology Society Yearbook 2011: 94–106. http://www.dendrology.org/publications/dendrology/cathaya-argyrophylla-some-little-known-facts/ [accessed 17 Oct. 2022]

[B8] Callaghan C, Png SK (2013) A new name and seventeen new combinations in the *Magnolia* (Magnoliaceae) of China and Vietnam. Botanical Studies 2013 54: 53/1–5. 10.1186/1999-3110-54-53 PMC5432815 28510891

[B9] Callaghan CB, Png SK (2020) Twenty-six additional new combinations in the *Magnolia* (Magnoliaceae) of China and Vietnam. PhytoKeys 146: 1–35. 10.3897/phytokeys.146.52114 PMC7205859 32405244

[B10] Callaghan CB, Png SK (2026a) Preservation or decimation for these reinstated species? An analysis of heterotypic synonyms contributes to 19 new names and 20 new combinations in *Magnolia* (Magnoliaceae). Part 1: New names. PhytoKeys (submitted 1 April 2026). 10.3897/phytokeys.277.195893 PMC13379712 42471902

[B11] Callaghan CB, Png SK (2026b) Unravelling the ill-conceived *Magnolia lacei* synonymy complex reveals the negative consequences of unsubstantiated synonymy to Magnoliaceae species. PhytoKeys (submitted 8 April 2026).

[B13] Chang HT (1961) Notulae Plantarum Austro-Sinicarum III. Acta Scientiarum Naturalium Universitatis Sunyatseni 1961(1): 53–56. [Chinese and Latin descriptions of new taxa]

[B14] Chen BL (1987) Four new species of *Michelia* from Yunnan. Acta Scientiarum Naturalium Universitatis Sunyatseni 1987(3): 86–91. [In Chinese] http://xuebao.sysu.edu.cn/Jweb_zrb/EN/Y1987/V26/I3/86 [accessed 25 Oct. 2022]

[B15] Chen BL, Nooteboom HP (1993) Notes on Magnoliaceae III: The Magnoliaceae of China. Annals of Missouri Botanical Garden 80(4): 999–1104. 10.2307/2399942

[B16] Chen BL, Yang SC (1988) New taxa of Magnoliaceae from Yunnan, China. Acta Scientiarum Naturalium Universitatis Sunyatseni 1988(3): 94–99. [In Chinese] http://xuebao.sysu.edu.cn/Jweb_zrb/EN/Y1988/V27/I3/94 [accessed 26 Oct. 2022]

[B17] Chen YK, Yang Q, Mo YN, Yang X, Dong H, Hong XJ (2014) A study on the niches of the state’s key protected plants in Bawangling, Hainan Island. Chinese Journal of Plant Ecology 38(6): 576–584. [In Chinese, English subtitle / abstract] 10.3724/SP.J.1258.2014.00053

[B18] Chien PD, Werger MJA, Nghia NH (2006) Chapter 2: Vietnamese forestry, biodiversity and threatened tree species. In: Chien PD (Ed.) Demography of Threatened Tree Species in Vietnam. Thesis, Utrecht University, Utrecht, Netherlands, 19–54. [Vietnamese subtitle/summary] https://dspace.library.uu.nl/bitstream/handle/1874/15051/full.pdf?sequence=5&isAllowed=y [accessed 30 Mar. 2020]

[B19] Chien SS, Cheng WC, Pei C (1936) An enumeration of vascular plants from Chekiang IV. Contributions from the Biological Laboratory of the Science Society of China, Botanical Series 10(2): 93–155.

[B20] Cicuzza D, Newton A, Oldfield S (2007) The Red List of Magnoliaceae. Fauna & Flora International, Cambridge, UK. https://portals.iucn.org/library/sites/library/files/documents/RL-2007-001.pdf [accessed 30 Mar. 2020 – see updated version at Rivers et al. 2016]

[B21] Craib WG (1922) Contributions to the flora of Siam. Additions XII. Bulletin of Miscellaneous Information, Royal Botanic Gardens, Kew 1922: 165–174. [*Michelia kerrii* p. 166] 10.2307/4113362

[B22] Dandy JE (1927) The genera of Magnolieae. Bulletin of Miscellaneous Information, Royal Botanic Gardens, Kew 1927(7): 257–264. 10.2307/4107601

[B23] Dandy JE (1928a) *Michelia montana* and two allied new species. The Journal of Botany: British and Foreign 66(11): 319–322. http://archive.bsbi.org.uk/Journal_of_Botany_1928.pdf [accessed 17 Apr. 2020]

[B24] Dandy JE (1928b) Two new michelias from Kwangtung. The Journal of Botany: British and Foreign 66(12): 359–361. http://archive.bsbi.org.uk/Journal_of_Botany_1928.pdf [accessed 17 Apr. 2020]

[B25] Dandy JE (1928c) New or noteworthy Chinese Magnolieae. Notes from the Royal Botanic Garden, Edinburgh 16(77): 123–133. 10.2307/4107183

[B26] Dandy JE (1929a) A new *Michelia* from the borders of Tibet and Assam. Bulletin of Miscellaneous Information, Royal Botanic Gardens, Kew 1929(7): 222–223. [*Michelia wardii*] 10.2307/4113536

[B27] Dandy JE (1929b) Three new michelias from Indo-China. The Journal of Botany: British and Foreign 67(8): 222–224. http://archive.bsbi.org.uk/Journal_of_Botany_1929.pdf [accessed 17 Apr. 2020]

[B28] Dandy JE (1930) New Magnolieae from China and Indo-China. The Journal of Botany: British and Foreign 68(7): 204–214.

[B29] Dandy JE (1931) Four new Magnolieae from Kwangsi. The Journal of Botany: British and Foreign 69(9): 231–233.

[B30] de Candolle AP (1818) [not 1817 as often noted] Regni Vegetabilis Systema Naturale, sive Ordines, Genera et Species Plantarum Secundum Methodi Naturalis Normus Digestarum et Descriptarum Vol. 1. Treuttel and Würtz, Paris. [In Latin]. [*Michelia doltsopa* Buch.-Ham. ex DC., p. 448] 10.5962/bhl.title.59874

[B31] Deng LX, Yang XY, Yang CH, Zhou JW, Chen ZP, Shen JM (2015) Magnoliaceae Plants of Guizhou. Guizhou Science and Technology Publishing House, Guiyang. [In Chinese]

[B32] Deng YF (2009) Magnoliaceae. In: Wu ZY, Raven PH, Hong DY (Eds) Flora of China Illustrations Vol. 7, Menispermaceae through Capparaceae: 54–107. Science Press, Beijing and Missouri Botanical Garden Press, St. Louis. *Michelia sphaerantha*^1^, p. 92; *Michelia martini*^2^, p. 103]. http://www.efloras.org/object_page.aspx?object_id=112192&flora_id=2^**1**^http://www.efloras.org/object_page.aspx?object_id=112532&flora_id=2^**2**^ [both accessed 10 Mar. 2020]

[B33] Dunn ST (1908) A botanical expedition to central Fokien. Journal of the Linnean Society of London (Botany) 38, Issue 267: 350–373. [*Michelia skinneriana*, p. 354]. 10.1111/j.1095-8339.1908.tb00858.x

[B34] Elliott S (1994) The effects of urbanization on Doi Suthep-Pui National Park. In: Proceedings of the International Symposium on Urbanization and Forests, 14–15 December 1994, Chiang Mai University, Thailand: 1–12. Chiang Mai University, Chiang Mai, Thailand. https://www.forru.org/sites/default/files/public/publications/resources/forru-0000234-0003-en.pdf [accessed 17 Oct. 2022]

[B35] Figlar RB (2000) Proleptic branch initiation in *Michelia* and Magnolia subgenus Yulania provides basis for combinations in subfamily Magnolioideae. In: Liu YH, Fan HM, Chen ZY, Wu QG, Zeng QW (Eds) Proceedings of the International Symposium on the Family Magnoliaceae, Guangzhou, China, 18–22 May 1998. Science Press, Beijing, 14–25.

[B36] Finet AE, Gagnepain F (1906a) Contributions à l’étude de la flore de l’Asie orientale. Bulletin de la Société Botanique de France Memoires 1905. 4(52): 1–170 + 20 pl. [In Latin and French] [*Michelia yunnanensis* Franch.^1^ mss. pp. 43–44, 47, pl. 6; *Michelia floribunda* Finet & Gagnep.^2^ p. 46.]. https://biodiversitylibrary.org/page/508451^1^ Note: Plate 6 not found at this URL. https://biodiversitylibrary.org/page/508454 **^2^** [both accessed 30 Mar. 2020]

[B37] Finet AE, Gagnepain F (1906b) Espèces nouvelles de l’Asie orientale. Bulletin de la Société Botanique de France 53: 573–576. [In Latin and French] [*Michelia cavaleriei*, p. 573; *M. bodinieri*, p. 574]. 10.1080/00378941.1906.10831204

[B38] Frodin DG, Govaerts R (1996) World Checklist and Bibliography of Magnoliaceae. Information Services Dept., Royal Botanic Gardens, Kew.

[B39] Fu LG, Jin JM [Eds] (1992) China Plant Red Data Book. Rare and Endangered Plants, Vol. 1. Science Press, Beijing, New York. [accounts of 388 threatened taxa – unusual that Vol. 2 has never been compiled and published!]

[B40] Govaerts R (2003) World Checklist of Magnoliaceae (online). Facilitated by the Royal Botanic Gardens, Kew. https://wcsp.science.kew.org/ [accessed 12 Oct. 2022]

[B41] Govaerts R (2022) World Checklist of Selected Plant Families (WCSP online: Magnoliaceae). Facilitated by the Royal Botanic Gardens, Kew. https://wcsp.science.kew.org/ [accessed 12 Oct. 2022]

[B42] Govaerts R, Nooteboom HP (2010) *Talauma gioi* A Chev. In: Brummitt R (Ed.) Report of the Nomenclature Committee for Vascular Plants. Taxon 59(4): 1276. [Recommendation of committee, p. 1277]. 10.1002/tax.594025

[B43] Grimshaw J, Bayton R (2009) New Trees–Recent Introductions to Cultivation. International Dendrology Society, Kew Publishing, Royal Botanic Gardens Kew. Also frequently updated version Trees and Shrubs Online. https://www.treesandshrubsonline.org/

[B44] Guan ZB, Zhang LX (2004) The study on conservation and utilization of endangered plant *Michelia hedyosperma*. Chinese Wild Plant Resources 23(4): 11–14. [In Chinese, English subtitle/abstract] https://www.docin.com/p-5595116.html [accessed 3 Feb. 2026]

[B45] Hà CTT, Thành BV, Thùy ĐTT (2020) Chemical composition and antimicrobial activity of the essential oil from leaves of *Magnolia coriacea* (Hung T Chang & BL Chen) Figlar growing in Vietnam. Academia Journal of Biology 42(3): 135. 10.15625/2615-9023/v42n3.14739

[B46] Handel-Mazzetti H (1921) Plantae novae sinenses, diagnosibus brevibus descriptae a D^re^. Heinr. Handel-Mazzetti. Anzeiger der Akademie der Wissenschaften in Wien. Mathematische-Naturwissenschaftliche Klasse 58(12 cont’d): 145–154. [In German and Latin] [*Michelia microtricha* p. 145]. https://www.biodiversitylibrary.org/page/27808730#page/173/mode/1up [accessed 18 Dec. 2022]

[B47] Hao J, Pan LQ, Jia HY, Jiang QB, Pan QL, Pinyopusarerk K, Kalinganir A (2019) Floral structure and breeding systems of *Manglietia conifera* Dandy (Magnoliaceae). Forests 2019, 10, 756. 10.3390/f10090756

[B48] Hill M, Hallam D [Eds] (1997) Na Hang Nature Reserve, Tat Ke Sector. Site Description and Conservation Evaluation. Frontier-Vietnam Environmental Research Report 9. Ministry of Agriculture and Rural Development (Forest Protection Department), Frontier-Vietnam, Institute of Ecology and Biological Resources, Society for Environmental Exploration, Hanoi, Vietnam. https://citeseerx.ist.psu.edu/document?repid=rep1&type=pdf&doi=c3f5cedcacbf7e4a825ee558f141b87123d17c8c [accessed 30 Mar. 2020]

[B49] Huang CM, Zou TC (2002) Study on peculiar ornamental wild species germ plasma and its effective utilization from Magnoliaceae and Ericaceae in Guizhou. Journal of Guizhou University (Natural Science) 19(2): 169–176. [In Chinese, English end title / abstract] http://www.doc88.com/p-1814671463792.html [accessed 30 Mar. 2020]

[B50] Huang LF (2007) RAPD and ISSR Analysis of 20 Species from 6 Genera of Magnoliaceae. Master’s Thesis (Biochemistry and Molecular Biology). Fujian Normal University, Fuzhou. ii + 73 pp. [In Chinese, English abstract] https://www.doc88.com/p-74561296823607.html [accessed 6 Feb. 2026]

[B51] IUCN (2026) The IUCN Red List of Threatened Species. Version 2025-2. International Union for the Conservation of Nature and Natural Resources. Gland, Switzerland and Cambridge, UK. https://www.iucnredlist.org/species/193957/2292193 [accessed 5 Feb. 2026]

[B52] Jin JM et al. (1992) *Manglietia aromatica*, p. 430. In: Fu LK, Jin JM (Eds) China Plant Red Data Book: Rare and Endangered Plants. Vol. 1. Science Press, Beijing, New York. 741 pp.

[B53] Khuraijam JS, Goel AK (2015) Enumeration of the genus *Magnolia* L. in India with its conservation status. Phytotaxonomy 15: 107–113.

[B54] Krüssmann G [Translator: Epp ME] (1985) Manual of Cultivated Broad-leaved Trees and Shrubs Vol. II, E–PRO. Timber Press, Portland, Oregon. [English translation of previous entry: *Manglietia* p. 298; *Michelia* 306–307].

[B55] Kumar VS (2006) New combinations and new names in Asian Magnoliaceae. Kew Bulletin 61(2): 183–186. [*Magnolia tibetica* p.185]. https://www.jstor.org/stable/20443258?seq=1 [accessed 20 Mar. 2020 (partial)]

[B56] Law YW (1985) Taxa nova Magnoliacearum. Bulletin of Botanical Research (Harbin) 5(3): 121–131. [In Chinese with Latin abstract] http://bbr.nefu.edu.cn/EN/Y1985/V5/I3/121 [accessed 30 Mar. 2020]

[B57] Law YW (1996) Magnoliaceae. In: Law YW, Lo HS, Wu YF, Chang BN (Eds) Flora Reipublicae Popularis Sinicae 30(1): Angiospermae, Dicotyledoneae: Menispermaceae, Magnoliaceae: 82–198. Science Press, Beijing. [In Chinese] http://www.iplant.cn/info/Trib.%20MAGNOLIEAE?t=z [accessed from 28 Mar. 2020]

[B58] Law YW, Wu YF (1988) Two new species of *Michelia*. Bulletin of Botanical Research (*Harbin*) 8(3): 71–75. [In Chinese, English subtitle] http://bbr.nefu.edu.cn/EN/Y1988/V8/I3/71 [accessed 30 Mar. 2020]

[B59] Lee SC (1935) Forest Botany of China. Commercial Press Ltd., Shanghai.

[B60] Léveillé H (1911) Decades plantarum novarum LIX–LXX. Feddes Repertorium Specierum Novarum Regni Vegetabilis 9(27–31): 441–463 [*Michelia cavaleriei*, p. 459]. 10.1002/fedr.19110092706

[B61] Léveillé H (1914–1915) Flore du Kouy-Tchéou. (=Flora of Guizhou), Le Mans, France. Lithographed from handwritten manuscript. [In French]. [*Magnolia*, *Manglietia*, *Michelia*, pp. 269–270]. https://www.biodiversitylibrary.org/item/9613#page/258/mode/1up [accessed 5 Jan. 2023]

[B62] Li H, Wei XQ, Zhang JC, Kong DJ, Yang CH (2022) Five new records of spermatophyte in Guizhou. Guizhou Forestry Science and Technology 50(2): 62–64. [In Chinese, English subtitle plus abstract] https://www.doc88.com/p-70799745777161.html [accessed 3 Feb. 2026]

[B63] Li J (1997) Some notes on Magnoliaceae from China. Acta Botanica Yunnanica 19(2): 131–138. [In Chinese, English subtitle / abstract] https://journal.kib.ac.cn/EN/abstract/abstract31371.shtml [accessed 25 Oct. 2022]

[B64] Li J (2013) Analysis of Genetic Diversity of 21 Kinds of Magnoliaceae Evergreen Plants. Master’s Thesis (Garden Plants and Ornamental Horticulture), Henan Agricultural University, Zhengzhou. [In Chinese, English end title / abstract] http://www.doc88.com/p-6863728673512.html [accessed 30 Mar. 2020]

[B65] Li JJ (2021) Analysis of Genetic Relationship of Magnoliaceae Based on ISSR. Master’s Thesis (Agronomy), Central South University of Forestry and Technology, Changsha, Hunan. [In Chinese, English subtitle / abstract] https://www.doc88.com/p-14787848196166.html [accessed 15 Nov. 2022]

[B66] Li JJ, Peng JQ, Wu Y, Wang YL, Liao TZ, Ma B, Xue C, Yu LQ, Xie QY, Wang HY, Cao JW (2020) Genetic diversity among the species of *Michelia* in China using ISSR. International Journal of Agriculture and Biology 413–419. 10.17957/IJAB/15.1453

[B67] Li SF (2008) Community Ecology of Meso-humid Evergreen Broad-leaved Forest in the East Slope of Ailao Mountain, Honghe Nature Reserve—Also on the Geographical Barrier Effect of the Ailao Mountains. Master’s Thesis (Botany), Southwest Forestry College, Kunming, Yunnan, China. [In Chinese, English abstract] http://www.doc88.com/p-7863342645046.html [accessed 30 Mar. 2020]

[B68] Li SH, Qin DW, Wei ZM, Qin WM, Nie ZZ (2015) Research on the photosynthesis of *Manglietia tenuipes* under drought stress. Northern Horticulture 2015(3): 72–76. [In Chinese, English end title / abstract] 10.11937/bfyy.201503023

[B69] Li TZ, Zhang SY, Deng YF, Li YL (2024) Comparative analysis of chloroplast genomes for the genus *Manglietia* Blume (Magnoliaceae): Molecular structure and phylogenetic evolution. Genes 15(4): 406. [included in special issue Molecular Mechanisms of Adaptive Evolution in Trees]. 10.3390/genes15040406 PMC11048997 38674341

[B70] Li X, Li XL, Ma TY, Wang YB, Chen FY (2010) Analyses of the genetic relationships of 22 species of *Manglietia* plant using ISSR markers. pp. 193–194. In: Proceedings of the 3^rd^ International Conference of Plant Molecular Breeding, Sept. 5–9, 2010, Beijing, China. https://www.docin.com/p-1333915371.html [accessed 23 Mar. 2026]

[B71] Li YK, Wang XM (1983) Three new species of trees in Guizhou. Guizhou Science 1983(2): 18–24. [In Chinese]. [incl. *Michelia chongjiangensis*] https://oversea.cnki.net/kcms/detail/detail.aspx?dbcode=CJFD&filename=GZKX198302003&dbname=CJFD7984 [accessed 30 Mar. 2020]

[B72] Li YK, Wang XM (1987) A new species of *Michelia* from Guizhou. Acta Phytotaxonomica Sinica 25(5): 408–409. [In Chinese and Latin, English subtitle] https://www.jse.ac.cn/EN/Y1987/V25/I5/408 [accessed 27 Mar. 2020]

[B73] Li YY, Li DX (1999) Conservation value and exploitation foreground of the Magnoliaceae plants in Yunnan. Journal of Beijing Forestry University 21(3): 29–35. [In Chinese, English subtitle / abstract] https://www.docin.com/p-475559447.html [accessed 30 Mar. 2020]

[B74] Li YY, Sima YK, Fang B, Guo LQ, Jiang H, Zhao WS (2003) Current situation and evaluation of natural resources of the priority protection wild plants in Yunnan Province of China. Acta Botanica Yunnanica 25(2): 181–191. [In Chinese, English subtitle / abstract] https://journal.kib.ac.cn/EN/abstract/abstract32721.shtml [accessed 30 Mar. 2020]

[B75] Lian CZ, Chen GD, Yang WF (2013) Describes[sic] the *Manglietia hainanensis* is reviewed. Tropical Forestry 41(3): 34–36. [In Chinese, English subtitle / abstract] http://www.doc88.com/p-8995397945380.html [accessed 30 Mar. 2020]

[B76] Linghu KH, Qin LJ, Chen ZG, Liu SF, Xiong ZB, Wang CS (2012) The status and protection of rare and endangered plant *Manglietia calcarea* X. H. Song in Maolan National Nature Reserve. Chinese Agricultural Science Bulletin 28(28): 100–103. [In Chinese, English subtitle / abstract] https://www.casb.org.cn/EN/abstract/abstract13757.shtml [accessed 26 Oct. 2022]

[B77] Liu H, Xu QY, He PC, Santiago LS, Yang KM, Ye Q (2015) Strong phylogenetic signals and phylogenetic niche conservatism in ecophysiological traits across divergent lineages of Magnoliaceae. Nature / Scientific Reports 5(12246): 1–12. 10.1038/srep12246 PMC4503962 26179320

[B78] Liu HY (2007) Magnoliaceae. In: Studies on Introduction, Propagation and Cultivation Techniques of Important Wild Ornamental Plants from Guizhou: 46–48. Master’s Thesis (Botany) Guizhou Normal University, Guiyang, China. [In Chinese, English subtitle / abstract] http://cdmd.cnki.com.cn/Article/CDMD-10663-2007214763.htm [accessed 30 Mar. 2020]

[B79] Liu XJ, Tang SJ, Sun QM, Yao Z (2008) Geographic distribution of Magnoliaceae plants in East China Plant Zone. Forest Inventory and Planning 33(5): 40–43. [In Chinese, English subtitle / abstract] https://www.docin.com/p-705256228.html [accessed 2 Feb. 2026]

[B80] Liu YH, Wu RF (1988) Some new taxa of *Michelia* from China. Acta Botanica Yunnanica 10(3): 335–342. [In Chinese and Latin, English subtitle] https://journal.kib.ac.cn/EN/abstract/abstract32082.shtml [accessed 25 Oct. 2022]

[B81] Liu YH, Zeng QW, Zhou RZ, Xing FW [Eds] (2004) Magnolias of China. Baitong Group. Beijing Science & Technology Press, Beijing. [In Chinese and English]

[B82] Lu SG, Shui YM (2002) Magnoliaceae. In: A Comprehensive Scientific Survey Report of Wenshan Nature Reserve in Yunnan, 113–121 + 328–?. Southwest Forestry College, Kunming, Yunnan, China. [In Chinese] https://www.docin.com/p-134534714.html [accessed to p. 328 on 30 Mar. 2020]

[B83] Luo Y, Luo W, Zhao TX, Yang Y, Yuan L, Zhang PZ, Gong ZX, Li HZ, Sima YK, Xu T (2025) The complete chloroplast genomes of three *Manglietia* species and phylogenetic insight into the genus *Manglietia* Blume. Current Issues in Molecular Biology 47(9): 737 10.3390/cimb47090737 PMC12468266 41020858

[B84] Ministry of Environmental Protection & Chinese Academy of Sciences (2014) Magnoliaceae. In: The Chinese Red List of Biodiversity—Higher Plants Volume. [aka: Red List of China Higher Plants]: 376–378.

[B85] Mitchell A, Coombes A (1998) The Garden Tree. An Illustrated Guide to Choosing, Planting and Caring for 500 Garden Trees. George Weidenfeld and Nicolson Ltd., Orion Publishing Group, London.

[B86] Nguyễn QH, Nguyễn TH, Từ BN, Nguyễn SK (2015) Preliminary studies on the diversity of magnolias of Ha Giang Province and the assessment of their conservation status. In: Long KD (Ed.) Proceedings of the Sixth National Scientific Conference on the Ecology and Biological Resources, Hanoi, Vietnam, 21 October 2015: 130–136 Institute of Ecology and Biological Resources, Hanoi, Vietnam. [In Vietnamese, English end title / summary] http://iebr.ac.vn/database/HNTQ6/130.pdf [accessed 30 Mar. 2020]

[B87] Nooteboom HP (1985) Notes on Magnoliaceae, with a revision of *Pachylarnax* and *Elmerrillia* and the Malesian species of *Manglietia* and *Michelia*. Blumea 31: 65–121. [*Magnolia yunnanensis* (Cheng & Hu) Noot. *comb*. nov. p. 88; *Michelia figo* (Lour.) Spreng., pp. 120–121]. Note: the published author for the basionym provided by *Parakmeria yunnanensis* is Hu only. http://www.repository.naturalis.nl/document/565414 [accessed 30 Mar. 2020]

[B88] Nooteboom HP, Chalermglin P (2009) The Magnoliaceae of Thailand. Thai Forest Bulletin (Botany) 37: 111–138. http://www.tci-thaijo.org/index.php/ThaiForestBulletin/article/viewFile/24341/20711 [accessed 3 Mar. 2020]

[B89] Oliver D [Ed.] (1891) Hooker’s Icones Plantarum: or Figures with Descriptive Characters and Remarks, of New and Rare Plants, Selected from the Kew Herbarium. Third Series. Williams and Norgate, London and Edinburgh / R. Friedländer und Sohn, 11, Carlstrasse, Berlin. [*Manglietia fordiana* Oliver, pl. 1953]. https://biodiversitylibrary.org/page/16235020 [accessed 30 Mar. 2020]

[B90] Peng RC (2013) Studies on Vascular Plants of Mulun National Nature Reserve, Guangxi, China. Master’s Thesis (Botany), Guangxi Normal University, Guilin, Guangxi, China. [In Chinese, English subtitle / abstract] http://cdmd.cnki.com.cn/Article/CDMD-10602-1013246063.htm [accessed 7 Apr. 2020]

[B91] Peng ZH (2008) Urban and Rural Arbores in China. China Forestry Publishing House, Beijing. [*Manglietia fordiana*, pp. 84–85; *Michelia macclurei*, pp. 86–87]

[B92] Polunin O, Stainton S (1999) Flowers of the Himalaya. Oxford University Press, Dehli. (Third Oxford India impression). [*Michelia doltsopa*, p. 19, plate 10, No. 74]

[B93] Primack RB (1988) Forestry in Fujian Province, People’s Republic of China, during the Cultural Revolution. Arnoldia 48(2): 26–29. 10.5962/p.258585

[B94] Putiyanan S, Maxwell JF (2006) Survey and herbarium specimens of medicinal vascular flora of Doi Suthep-Pui. Chiang Mai University Journal of Natural Sciences 5(2): 169–178. http://cmuj.cmu.ac.th/uploads/journal_list_index/814893439.pdf [accessed 30 Mar. 2020]

[B95] Qin HN, Yang Y, Dong SY, He Q, Jia Y, Zhao LN, Yu SX, Liu HY, Liu B, Yan YC, Xiang JY, Xia NH, Peng H, Li ZY, Zhang ZX, He XJ (2017) Magnoliaceae. In: Threatened species list of China’s higher plants: 725. Biodiversity Science 25(7): 696–744. [In Chinese, English subtitle / abstract] [lists species considered Critically Endangered (CR), Endangered (EN) and Vulnerable (VU)].

[B96] Rivers M, Beech E, Murphy L, Oldfield S (2016) The Red List of Magnoliaceae: Revised and Extended. Botanic Gardens Conservation International, Richmond, Surrey. U.K. http://globaltrees.org/wp-content/uploads/2016/03/Magnoliaceae_RedList2016_LowRes.pdf [accessed 30 Mar. 2020]

[B97] Sang ZY, Zhang DC, Wang YB, Ma LY (2016) Taxonomy significance based on pollen morphology observation of 19 species of *Manglietia*. Bulletin of Botanical Research 36(1): 43–51. [In Chinese, English abstract] https://www.docin.com/p-1482933733.html [accessed 15 Apr. 2026]

[B98] Shui YM [Ed.] (2003) Seed Plants of Honghe Region in SE Yunnan, China. Yunnan Science and Technology Press. Kunming. [In Chinese and English]

[B99] Shui YM, Mao LH, et al. (2016) Seed Plants of Mount Xilong, the Highest Mountain in South Yunnan, China. Science Press, Beijing. [Michelia floribunda var. lanea p. 130]

[B100] Sima YK (2001) Some notes on Magnolia subgenus Michelia from China. Yunnan Forestry Science and Technology 2001(2): 29–35, 40. [In Chinese, English end title / abstract] https://www.doc88.com/p-7744202802175.html [accessed 17 Oct. 2022]

[B101] Sima YK (2011) A Taxonomic Revision of the Magnoliaceae from China. PhD. Dissertation (Botany). School of Life Science, Yunnan University, Kunming, China. [In Chinese, English subtitle / abstract] https://www.doc88.com/p-0408321349844.html [accessed 27 Mar. 2020]

[B102] Sima YK, Lu SG (2009) Magnoliaceae. In: Shui YM, Sima YK, Wen J, Chen WH (Eds) Vouchered Flora of Southeast Yunnan, Vol. 1: 16–79. Yunnan Science & Technology Press, Kunming. http://www.doc88.com/p-9455111883790.html [accessed 30 Mar. 2020]

[B103] Sima YK, Lu SG, Hao JB, Xu T, Han MY, Xu L, Li D, Ma HF, Chen SY (2014) Notes on Manglietia aromatica var. calcarea (Magnoliaceae), a variety endemic to south-western China. Journal of West China Forestry Science 43(1): 96–98. [In English, Chinese abstract, unrelated Chinese subtitle] http://xblykx.paperopen.com/OA/pdfdow.aspx?Sid=17%BA%C5 [accessed 25 Oct. 2022]

[B104] Song XH (1984) New taxa and new distributions of woody plants from Guizhou Province. Journal of Nanjing Institute of Forestry 1984(4): 46–52. [In Chinese and Latin, English subtitle] 10.1002/he.36919844505

[B105] Spencer R (1997) Magnoliaceae. In: Horticultural Flora of South-eastern Australia Volume 2. Flowering Plants, Dicotyledons Part 1: 7–24. University of New South Wales Press Ltd., Sydney, Australia.

[B106] Spongberg SA (1998) Magnoliaceae Hardy in Cooler Temperate Climates. In: Hunt D (Ed.) Magnolias and their Allies. Proceedings of an International Symposium, Royal Holloway, University of London, Egham, Surrey, U.K., 12–13 April, 1996: 81–144. International Dendrology Society and The Magnolia Society. David Hunt, Milborne Port, Sherborne, U.K.

[B107] Sun WB, Yan L, Magin G, Zhou Y (2007) Conservation of Magnoliaceae in China: Five flagship species in Yunnan, SW China. In: Proceedings of the 3^rd^ Global Botanic Gardens Congress, 15–20 April 2007, Wuhan, China: 1–5. https://www.bgci.org/files/Wuhan/PapersConserving/SunMagnolia.pdf [accessed 21 Nov. 2022]

[B108] Sutton J (2022) *Manglietia fordiana*. Trees and Shrubs Online. https://www.treesandshrubsonline.org/ [accessed 21 Mar. 2026]

[B109] Tang ZZ (1982) Natural tropical forests in Hainan Island. Journal of South China Normal University (Natural Science) 1982(2): 114–122. [In Chinese, English end title / abstract] http://journal-n.scnu.edu.cn/article/id/2474 [accessed 25 Oct. 2022]

[B110] Tiêṕ NV (1980) Beiträge zur sippenstruktur der gattung *Manglietia* Bl. (Magnoliaceae). Feddes Repertorium 91(9–10): 497–576. [In German with English summary] 10.1002/fedr.19800910902

[B111] Turland NJ, Wiersema J, Barrie FR, Gandhi KN, Gravendyck J, Greuter W, Hawkesworth DL, Herendeen PS, Klopper RR, Knapp S, Kusber W-H, Li D-Z, May TW, Monro AM, Prado J, Price MJ, Smith GF, Señoret JCZ (2025) International Code of Nomenclature for algae, fungi, and plants (Madrid Code) accepted by the Twentieth International Botanical Congress, Madrid, Spain, July 2024. University of Chicago Press, Chicago and London. https://www.iaptglobal.org/_functions/code/madrid [accessed 5 January 2026]

[B112] Vũ QN, Xia NH (2010) *Michelia fulva* Chang et BL Chen (Magnoliaceae Juss.) A new record for the flora of Vietnam. Journal of Biology 32(2): 63–67. [In Vietnamese with English end title /summary] http://vjs.ac.vn/index.php/vjbio/article/viewFile/689/1192 [accessed 30 Mar.2020]

[B113] Vũ QN, Xia NH (2011) Some data on *Manglietia ventii* and *M. forrestii* (Magnoliaceae), little-known species of flora of Vietnam. Science and Technology Journal of Agriculture and Rural Development (Unspecified volume, pp. 182–185 [In Vietnamese]. https://agris.fao.org/search/en/providers/122620/records/64736643e17b74d222546d60 [accessed 18 Mar. 2026]

[B114] Vietnam Plant Database (2022) *Michelia fulva*. https://www.botanyvn.com/cnt.asp?param=news&newsid=1369&lg=en [accessed 30 Oct. 2022]

[B115] Vu NA (2017) Grassroots environmental activism in an authoritarium context: The trees movement in Vietnam. Voluntas: The International Journal of Voluntary and Nonprofit Organisations 28: 1180–1208 [E]. 10.1007/s11266-017-9829-1

[B116] Walter KS, Gillett HJ [Eds] (1998) Magnoliaceae. In: 1997 IUCN Red List of Threatened Plants: 387–388. IUCN, Gland, Switzerland and Cambridge, UK. (compiled by World Conservation Monitoring Centre for the IUCN Species Survival Commission). https://archive.org/stream/1997iucnredlisto97walt#page/n9/mode/2up [accessed 30 Mar. 2020]

[B117] Wang M, Peng JQ, Cao JW, Cao FX, Li JJ, Xue C, Wu Y (2020) Studies on genetic diversity of 48 wild species of Magnoliaceae in Yunnan. Journal of Tropical and Subtropical Botany 28(3): 277–284. [In Chinese, English subtitle / abstract] 10.11926/jtsb.4161

[B118] Wang S, Xie Y [Eds] (2004) China Species Red List. Vol. 1: Joint publication under the auspices of Chinese National Environmental Protection Agency and Chinese Endangered Species Scientific Commission. Higher Education Press, Beijing. [In Chinese and English, Latin plant names] [Determines the conservation status of each listed Chinese species in accordance with IUCN criteria]

[B119] Wang XP, Jiang GM (2001) The threatened status and protected measures of Magnoliaceae species in China. Journal of Plant Resources and Environment 10(4): 43–47. [In Chinese, English subtitle / abstract] http://zwzy.cnbg.net/Contribution/PeriodicalDirectoryDetails_86b8542d-116f-4bce-b597-8720422203cc.html [accessed 25 Oct. 2022]

[B120] Wang YL, Li Y, Zhang SZ, Cui TC, Wu XL (2003) The crossing result of Magnoliaceae. Journal of Wuhan Botanical Research 21(6): 508–514. [In Chinese, English subtitle / abstract] http://plantscience.cn/CN/article/downloadArticleFile.do?attachType=PDF&id=1908 [accessed 25 Oct. 2022]

[B121] Wei FN (1993) A new species of *Manglietia* Bl. from Guangxi. Guihaia 13(1): 5–6. [In Chinese, English subtitle] http://www.guihaia-journal.com/gxzw/ch/reader/create_pdf.aspx?file_no=199301003 [accessed 9 Mar. 2020]

[B122] Wei XL, Cao FX, Chen J (2010) Extraction of genomic DNA and optimization of ISSR reaction system of *Manglietia hainanensis*. Journal of Central South University of Forestry and Technology 30(5): 91–96. [In Chinese, English subtitle / abstract] http://www.docin.com/p-1594098144.html [accessed 2 Mar. 2020]

[B123] Wei XL, Cao FX, Chen J (2013) Studies on the genetic diversity and relationship of *Manglietia hainanensis* Dandy by ISSR. Biotechnology Bulletin 2013(8): 74–77. https://www.doc88.com/p-70287238603741.html [accessed 12 Mar. 2026]

[B124] Wen SN (2017) Genetic Diversity of Germplasm Resources of Manglietia conifera Dandy. PhD. Dissertation (Forest Genetics and Breeding), Chinese Academy of Forestry, China. [In Chinese, English subtitle / abstract] https://www.doc88.com/p-3177361844041.html [accessed 17 Oct. 2022]

[B125] Wu ZY, Chen SK (2006) Magnoliaceae. In: Flora Yunnanica. Tomus 6 (Spermatophyta): 1–62. Science Press, Beijing. [In Chinese] https://www.biodiversitylibrary.org/page/36698160#page/1 [accessed 28 Mar. 2020]

[B126] Xia NH, Liu YH, Nooteboom HP (2008) Magnoliaceae. In: Wu ZY, Raven PH, Hong DY (Eds) Flora of China Vol. 7, Menispermaceae through Capparaceae: 48–91. Science Press, Beijing and Missouri Botanical Garden Press, St. Louis. http://flora.huh.harvard.edu/china/mss/volume07/Magnoliaceae.pdf [accessed 20 Mar. 2020]

[B127] Xiao L, Li XL, Wang YB, Wang XQ, Chen FJ (2011a) Analysis of the genetic relationships among 22 species of *Manglietia* plants using ISSR markers. Bulletin of Botanical Research 31(4): 489–494. [In Chinese, English subtitle / abstract] 10.7525/j.issn.1673-5102.2011.04.017

[B128] Xiao L, Ma TY, Li XL, Chen FJ (2011b) Analyses of the genetic relationships among 22 species of *Manglietia* plants by SRAP markers. Acta Botanica Boreali-Occidentalia Sinica 31(11): 2178–2184. [In English with preceding Chinese title / abstract] http://www.doc88.com/p-902234543118.html [accessed 2 Mar. 2020]

[B129] Xing FW, Zeng QW, Chen HF, Wang FG [Eds] (2009) Magnoliaceae. In: Landscape Plants of China. Vol. 1: 177–217. Huazhong University of Science and Technology Press, Wuhan. [In Chinese]

[B130] Xiu XJ, He XY, Lin JX, Jiang CN, Huang YF (2013) Growth performance of 20 species of *Michelia* in Zhongshan Arboretum from Guangdong, China. Subtropical Plant Science 42(4): 342–344. [In Chinese]. [*Michelia fulgens*, Table 1 page 343]. 10.3969/j.issn.1009-7791.2013.04.014

[B131] Yan JA (2006) The Study on Evolutionary History of Hainan’s Ecological Environment—From the Point of Animal and Plant. PhD. Dissertation (History of Science and Technology), Nanjing Agricultural Uni., Jiangsu Province, China. [In Chinese, English subtitle / abstract] http://www.doc88.com/p-8456062244950.html [accessed 2 Mar. 2020]

[B132] Yang CH, Fang XP (2002) Landscape utilization prospects of Guizhou native Magnoliaceae plant resources. Guizhou Forestry Science and Technology 30(1): 20–25. [In Chinese]. http://www.doc88.com/p-9042659903302.html [accessed 20 Mar. 2020]

[B133] Yang KM, Chen XL, Gong X, Wang YL [Eds] (2016) Ex Situ Cultivated Flora of China (Magnoliaceae). Science Press, Beijing. [In Chinese, English subtitle]

[B134] Yang XY, Jiang H, Lin SG, Deng LX, Yang CH (2016) Community characteristics of *Michelia chongjiangensis* in Congjiang County, Guizhou. Guizhou Agricultural Sciences 2016(9): 144–147. [In Chinese, English subtitle / abstract] https://www.docin.com/p-4673038387.html [accessed 15 Apr. 2026]

[B135] Yang YM, Tian K, He SJ [Eds] (2008) Magnoliaceae. In: Study on the Scientific Survey of Wenshan National Nature Reserve in China: 481–484. Science Press, Beijing. [In Chinese, English subtitle / abstract, Chinese and Latin plant names]

[B136] Zhai DL, Xu JC, Dai ZC, Cannon CH, Grumbine RE (2014) Increasing tree cover while losing diverse natural forests in tropical Hainan, China. Regional Environmental Change 14(2): 611–621. 10.1007/s10113-013-0512-9

[B137] Zhang Z, Shen JM, Chen R, Li M (2022) Study on native *Manglietia* resources and landscape application in Guizhou. Guizhou Forestry Science and Technology 50(2): 47–50. [In Chinese, English subtitle / abstract] https://www.doc88.com/p-67187932006373.html [accessed 3 Feb. 2026].

[B138] Zhao XF, Ma YP, Sun WB, Wen XY, Milne R (2012) High genetic diversity and low differentiation of *Michelia coriacea* (Magnoliaceae), a Critically Endangered endemic in southeast Yunnan, China. International Journal of Molecular Sciences 2012, 13: 4396–4411. 10.3390/ijms13044396 PMC3344221 22605985

[B139] Zhou SB, Zhang DC (2002) A new species of the genus *Michelia* L. (Magnoliaceae) from China. Bulletin of Botanical Research 22(2): 129–130. [In Chinese with Latin description, English subtitle] http://bbr.nefu.edu.cn/EN/Y2002/V22/I2/129 [accessed 30 Mar. 2020]

[B140] Zou TC (2001) Study on germplasma resources and utilization evaluation for Guizhou endemic spermatophyte. Scientia Silvae Sinicae 37(3): 46–57. [In Chinese, English subtitle / abstract] http://www.linyekexue.net/CN/article/downloadArticleFile.do?attachType=PDF&id=3458 [accessed 17 Oct. 2022]

